# Propofol Protects Myocardium From Ischemia/Reperfusion Injury by Inhibiting Ferroptosis Through the AKT/p53 Signaling Pathway

**DOI:** 10.3389/fphar.2022.841410

**Published:** 2022-03-16

**Authors:** Shengqiang Li, Zhen Lei, Xiaomei Yang, Meng Zhao, Yonghao Hou, Di Wang, Shuhai Tang, Jingxin Li, Jingui Yu

**Affiliations:** ^1^ Department of Anesthesiology, Qilu Hospital, Cheeloo College of Medicine, Shandong University, Jinan, China; ^2^ Department of Anesthesiology, The Second Hospital, Cheeloo College of Medicine, Shandong University, Jinan, China; ^3^ Department of Physiology, School of Basic Medical Science, Cheeloo College of Medicine, Shandong University, Jinan, Shandong, China

**Keywords:** Akt, ferroptosis, heart, ischemia/reperfusion injury, propofol, p53

## Abstract

The molecular mechanism underlying the protective role of propofol against myocardial ischemia/reperfusion (I/R) injury remains poorly understood. Previous studies have shown that ferroptosis is an imperative pathological process in myocardial I/R injury. We hypothesized that propofol prevents myocardial I/R injury by inhibiting ferroptosis via the AKT/p53 signaling pathway. The ferroptosis-inducing agent erastin (E) and AKT inhibitor MK2206 (MK) were used to investigate the role of propofol in myocardial I/R injury. H9C2 cells treated without any reagents, erastin for 24 h, propofol for 1 h before adding erastin were assigned as the control (C), E, and E + P group, respectively. Cell viability, reactive oxygen species (ROS), and the expression of antioxidant enzymes, including ferritin heavy chain 1 (FTH1), cysteine/glutamate transporter (XCT), and glutathione peroxidase 4 (GPX4) in H9C2 cells. Rat hearts from the I/R + P or I/R groups were treated with or without propofol for 20 min before stopping perfusion for 30 min and reperfusion for 60 min. Rat hearts from the I/R + P + MK or I/R + MK groups were treated with or without propofol for 20 min, with a 10-min treatment of MK2206 before stopping perfusion. Myocardial histopathology, mitochondrial structure, iron levels, and antioxidant enzymes expression were assessed. Our results demonstrated that erastin increased H9C2 cell mortality and reduced the expression of antioxidant enzymes. I/R, which reduced the expression of antioxidant enzymes and increased iron or p53 (*p* < 0.05), boosted myocardium pathological and mitochondrion damage. Propofol inhibited these changes; however, the effects of propofol on I/R injury were antagonized by MK (*p* < 0.05). In addition, AKT siRNA inhibited the propofol-induced expression of antioxidant enzymes (*p* < 0.05). Our findings confirm that propofol protects myocardium from I/R injury by inhibiting ferroptosis *via* the AKT/p53 signal pathway.

## Introduction

Propofol (2,6-diisopropyl phenol), a popular intravenous anesthetic, is widely used in the induction and maintenance of general anesthesia (Bovill, 2006). Emerging data have shown that propofol exerts profound protective effects on myocardial ischemia/reperfusion (I/R) injury (Suleiman et al., 2015) by scavenging ROS (Zhao et al., 2019), reducing calcium overload (Zhou et al., 1997), and inhibiting polymorphonuclear neutrophils (PMN) adhesion (Szekely et al., 2000). Nevertheless, the specific underlying mechanism remains unclear.

Ferroptosis is a newly recognized form of cell death (Chen et al., 2020) that is distinct from apoptosis, necrosis, and autophagy (Macías-Rodríguez et al., 2020), characterized by cell membrane rupture and vesiculation, mitochondrial atrophy, ridge reduction, and nuclear-lacking chromatin aggregation (Yagoda et al., 2007; Yang and Stockwell, 2008). Under the action of divalent iron (Dixon et al., 2012) or iron-containing enzymes (Yang et al., 2016), unsaturated fatty acids of the cell membrane undergo lipid peroxidation to induce ferroptosis. The expression level of glutathione peroxidase 4 (GPX4) (Zhang et al., 2021), as the core enzyme of the antioxidant system, decreases during ferroptosis.

Ferroptosis is associated with a range of diseases, such as myocardial I/R injury, cancer, degenerative disease, and acute kidney injury (Han et al., 2020). As a critical role in the pathological process of myocardial injury (Stockwell et al., 2017), ferroptosis is closely related to p53 (Kruse and Gu, 2009). When cells are hypoxic, the stability and activity of p53 are enhanced. Subsequently, p53 enters the nucleus to regulate the expression of its downstream targets and promote the development of ferroptosis (Jiang et al., 2015). The activity of p53 is negatively regulated by AKT (also known as protein kinase B, PKB), a serine/threonine-specific protein kinase that is critical for regulating myocardial I/R injury (Yin et al., 2013). Phosphorylation of AKT promotes the combination of murine double minute 2 (MDM2) and p53, which leads to the degradation of p53 to inhibit ferroptosis (Freedman et al., 1999). A previous study has shown that propofol protects hepatic I/R injury by activating AKT phosphorylation (Wei et al., 2021) and p53 expression. Nevertheless, the effect of propofol on myocardial I/R injury remains unclear. Thus, we hypothesized that propofol may protect the myocardium by inhibiting myocardial ferroptosis through the AKT/p53 signaling pathway.

To test the above hypothesis, a series of *in vitro* and *in vivo* experiments were performed to investigate the effect and underlying mechanism of propofol on ferroptosis. This study demonstrates that propofol inhibits ferroptosis of both H9C2 cells and rat myocardium via the AKT/p53 signaling pathway, and it protects the myocardium from I/R injury.

## Materials and Methods

### Cell Culture

H9C2 cells were obtained from KeyGEN BioTECH (KG444, Nanjing, China), and cultured in high-glucose Dulbecco’s modified Eagle’s medium (DMEM, 0030034DJ, GIBCO, New York, USA) supplemented with 10% fetal bovine serum (FBS, 16140071, GIBCO, New York, USA) and 1% penicillin/streptomycin (15140122, GIBCO, New York, USA). The cells were incubated in a tri-gas incubator with 5% CO_2_ at 37°C.

### Cell Viability Assay

Cells were inoculated at 3,000–4,000/well in 96-well plates. After treatment, 10% cell counting kit-8 (CCK-8, HY-K0301, MCE, New Jersey, United States) reagent was added and incubated for 1–3 h. The absorbance was measured using a Microplate Reader (Thermo Fisher Scientific, Massachusetts, United States) at 450 nm.

### Experimental Grouping and Corresponding Treatment

**FIGURE 1:**
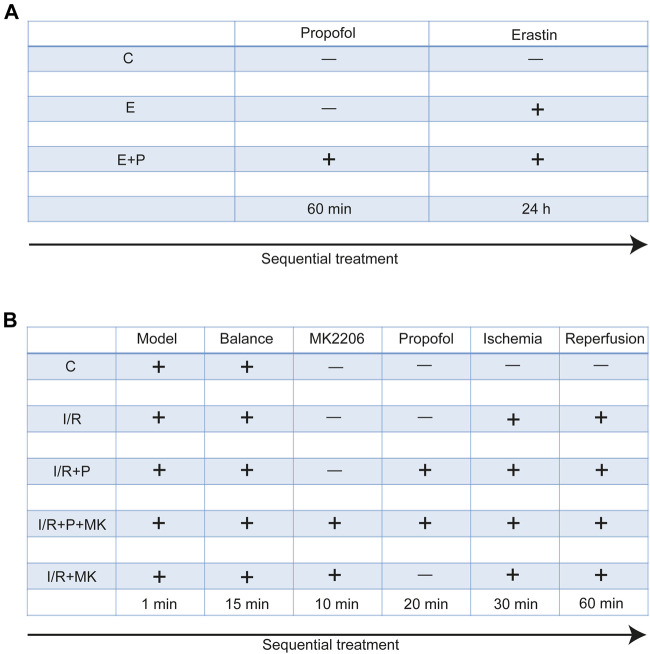
Experimental grouping and corresponding treatment. Experimental grouping and corresponding treatment on H9C2 cells **(A)** and rat hearts **(B)**.

H9C2 cells were treated with no reagents (C), erastin (5 μM, GC16630, California, GLPBIO, United States) for 24 h (E), and propofol (50 μM, D126608, Sigma, Missouri, United States) for 1 h before erastin (E + P). Each experiment was repeated three times (Figure 1A).

### Trypan Blue Assay

H9C2 cells were inoculated in 12-well plates and treated as described above. Next, 0.4% trypan blue solution (T8070, Solarbio, Beijing, China) was added to the cells for 3 min. Cell viability was calculated by a cell counter (Countstar^®^ BioTech, ALIT Life Science, Shanghai, China).

### Assessment of ROS Production

H9C2 cells were inoculated into 12-well plates overnight and treated as described above, then 10 µM dihydroethidium solution (DHE, S0063, Beyotime, Shanghai, China) was added to each well and the plates were incubated in the dark for 30 min at 37°C. The cells were observed under a fluorescence microscope (Nikon, Tokyo, Japan) and analyzed with Image J software (National Institutes of Health, Maryland, United States).

### Malondialdehyde, Iron Levels, and Superoxide Dismutase Activity Measurement

H9C2 cells from each group were collected and used to generate a 10% homogenate, which was placed at −20°C and repeatedly thawed on ice 3 times. The supernatant was centrifuged at 2,500 r/min for 10 min at 4°C. The MDA levels and SOD activity were determined according to the kit instruction (A003-1-2 and A001-3-2, Nanjing Jiancheng Bioengineering Institute, Nanjing, China). The iron levels in H9C2 cells were detected using a kit (DIFE-250, BioAssay Systems, California, USA) and in the myocardium were determined according to the kit instructions (A039-2-1, Nanjing Jiancheng Bioengineering Institute, Nanjing, China). The protein concentration was measured with a BCA kit (P0012S, Beyotime, Shanghai, China).

### Detection of Antioxidant Expressions in AKT siRNA-Transfecting H9C2 Cells

After incubation in 12-well plates for 24 h, the H9C2 cell medium was replaced with a serum-free medium (Opti-MEM I, Thermo Fisher Scientific, Massachusetts, United States). The siRNA (Gene Pharma, Shanghai, China) was diluted in serum-free medium and mixed with diluted Lipofectamine 2000 (Lipo 2000, Thermo Fisher Scientific, Massachusetts, USA) for 20 min at room temperature. Then, the mixture was added into 12-well plates and incubated for 6–8 h, replacing the medium with a complete medium once. After being incubated for 24 h, the cells were treated for follow-up experiments as the design above. Proteins were extracted from H9C2 cells and handled as western blot analysis. The siRNA sequence of AKT was as follows: F: 5′-CCA​AGC​ACC​GUG​UGA​CCA​UTT-3′, R: 5′-AUG​GUC​ACA​CGG​UGC​UUG​GTT-3′.

### Animals

Adult male rats (Wistar, weight 180–240 g), purchased from the Experimental Animal Center of Shandong University, were housed in a constant temperature (25 ± 2°C) room, fed with standard water and laboratory diet.

### Langendorff Heart Preparation

Rats were acclimatized to the laboratory environment for 3 days, anesthetized by 10% chloral hydrate, and sequentially injected with heparin (250 U/kg, H8060, Solarbio, Beijing, China). The hearts were rapidly resected and dipped into 40 ml ice-cold KH solution (pH 7.4, containing in mM: NaCl 115, MgSO_4_ 1.2, KCl 4.7, KH_2_PO_4_ 1.2, CaCl_2_ 1.8, glucose 11, and NaHCO_3_ 25). Finally, the hearts were cannulated using a retrofitted Langendorff apparatus and perfused with KH at a velocity of 10 ml/min, which was gassed with 5% CO_2_ and 95% O_2_ at 37°C. Myocardial ischemia was caused by stopping perfusion for 30 min, and reperfusion resulted from recanalization for another 60 min.

### Animal Grouping and Treatment

All rats were randomly divided into five groups. After stabilizing for 15 min, rat hearts were treated with KH for 120 min (C), with (I/R + P) or without (I/R) propofol for 20 min before stopping perfusion for 30 min and reperfusion for 60 min, with (I/R + P + MK) or without propofol (I/R + MK) after MK2206 (15 nM, GC16304, New York, GLPBIO, USA) for 10 min before stopping perfusion (N = 6, Figure 1B).

### MDA, Iron Levels, and Glutathione/Oxidized Glutathione (GSH/GSSG) Ratio Determination

After recanalization, the rat heart left ventricle anterior wall was cut. MDA, iron levels (A039-2-1, A039-2-1, Nanjing Jiancheng Bioengineering Institute, Nanjing, China), and the GSH/GSSG ratio (S0053, Beyotime, Shanghai, China) were assessed using their respective assay kits. The concentration of protein was detected by a BCA kit and the absorbance was determined with a microplate reader.

### Myocardial Fiber Assessment

Hearts were fixed overnight in 4% paraformaldehyde, dehydrated with alcohol, and made transparent with xylene. They were then fixed in paraffin and finally cut into 3–4-µm-thick slices, which were placed in xylene and alcohol and then stained with hematoxylin-eosin (HE) (C0105S, Beyotime, Shanghai, China). Finally, the sections were sealed with neutral resin and imaged under a microscope (Nikon, Tokyo, Japan).

### Mitochondrial Morphology Assessment

After reperfusion as described above, the left ventricle anterior wall was cut into a size of 2 mm × 5 mm × 10 mm and fixed overnight in electron microscope fixative (G1102, Servicebio, Wuhan, China). The sections were then embedded in phosphate buffer and baked in the oven. Sequentially, they were cut to a size 50–60 nm and stained with 2% uranyl acetate lead. Images were acquired under a transmission electron microscope (HITACHI, Tokyo, Japan).

### Western Blot Analysis

Proteins were extracted from H9C2 cells and myocardium lysates of the left ventricle anterior wall. Protein was separated by 12% SDS-PAGE, transferred to polyvinylidene fluoride (PVDF) membranes, blocked with 5% non-fatty milk for 60 min, and incubated with primary antibodies overnight at 4°C. Then, the membranes were incubated with secondary antibodies for 60 min and detected with enhanced chemiluminescence (ECL) detection system. Images were analyzed by ImageJ software.

### Immunofluorescence Assessment

After dewaxing and rehydrating according to a previous description, paraffin sections were repaired in citrate solution (P0083, Beyotime, Shanghai, China) for 30 min, blocked with 5% bovine serum albumin (BSA) for 60 min, and incubated with primary antibodies overnight at 4°C. Subsequently, they were incubated with secondary antibodies for 60 min and DAPI (C1002, Beyotime, Shanghai, China) for 15 min, followed by visualization using an immunofluorescence microscope and analyzed with ImageJ software.

### Antibodies

The following antibodies were used: anti-AKT (ab179463, Abcam, Cambridgeshire, UK), anti-AKT (phospho Ser473, 4060, CST, Boston, United States), anti-P53 (2,524, CST, Boston, United States), anti-P53 (phospho S392, ab33889, Abcam, Cambridgeshire, UK), anti-Ferritin (ab75973, Abcam, Cambridgeshire, UK), anti-GPX4 (ab125066, Abcam, Cambridgeshire, UK), anti-XCT (ab175186, Abcam, Cambridgeshire), anti-α-tubulin (ab7291,Abcam, Cambridgeshire, UK). HRP-conjugated secondary antibodies (ZB-2301 or ZB-2305, ZSGB-BIO, Beijing, China) and fluorochrome conjugated secondary antibody (ab150064, Abcam, Cambridgeshire).

### Statistical Analysis

Data are expressed as mean ± standard deviation (SD) and were analyzed by one-way ANOVA with Tukey’s post hoc test or the *t*-test. GraphPad Prism version 9.0 (San Diego, California, United States) was used for analysis, and a *p* value < 0.05 was considered to indicate a significant difference.

## Results

### Effect of Propofol on Erastin-Induced H9C2 Cells Ferroptosis

**FIGURE 2:**
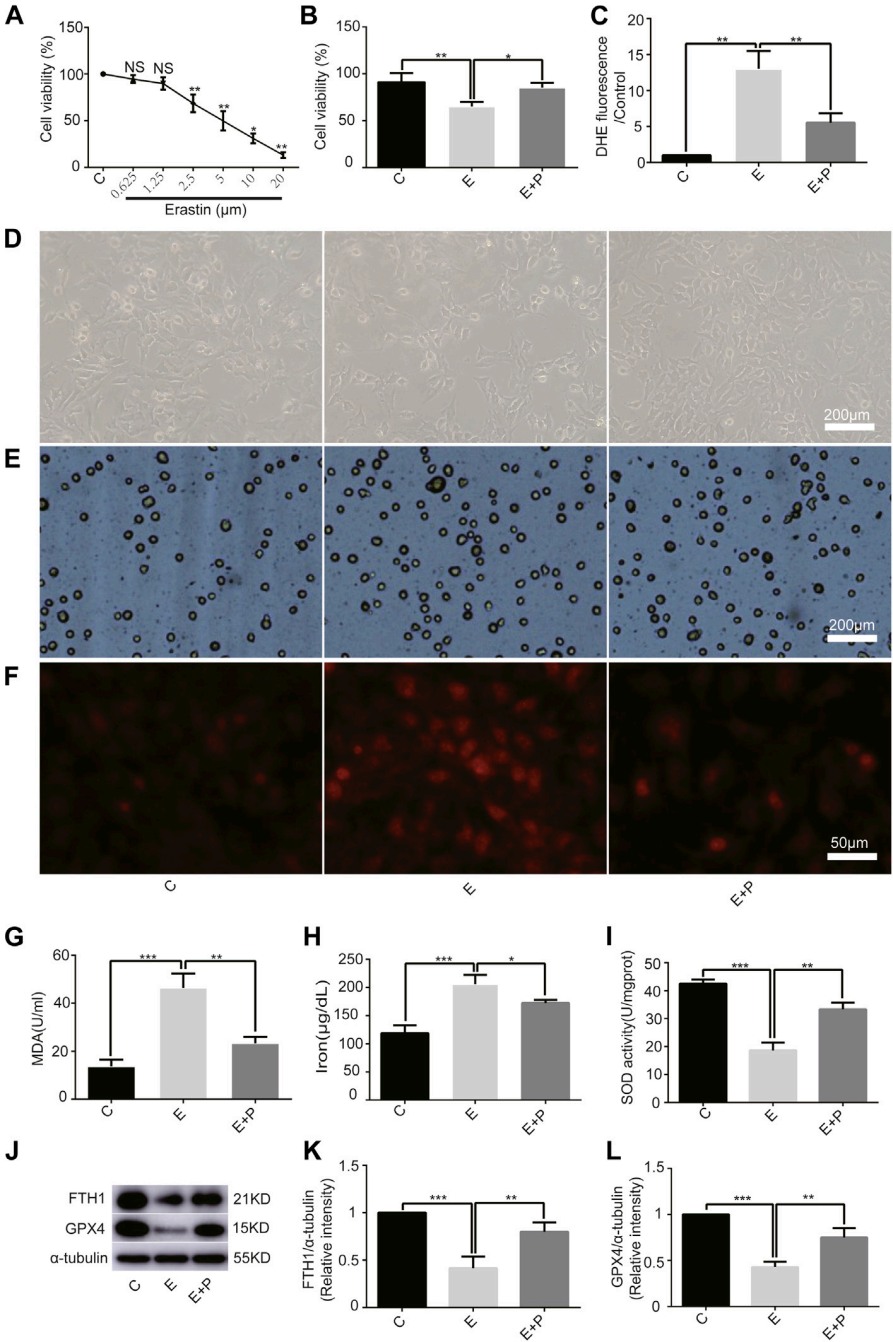
Effect of propofol on erastin-induced H9C2 cell ferroptosis. **(A)**. Cell viability of H9C2 cells treated with erastin (0–20 μM). **(B,D,E)**: Effect of propofol on erastin-induced cell death. Dead cells were stained with trypan blue. Scale bar: 200 μm. **(C**,**F)**: Effect of propofol on reactive oxygen species (ROS) production. Quantification of ROS is expressed as DHE. Scale bar: 50 μm. Effect of propofol on MDA **(G)**, iron **(H)**, and SOD **(I)** production. **(J–L)**: Influence of propofol on antioxidant enzymes (FTH1 and GPX4). N = 3. Data are expressed as the mean ± SD. Significance was calculated using one-way ANOVA with Tukey’s post hoc test or the *t*-test. *p*-values < 0.05 were considered statistically significant. *p < 0.05, **p < 0.01, ***p < 0.001.

First, we examined the effect of erastin on H9C2 cells and found a linear inhibition of cell viability with increasing erastin concentrations using the CCK-8 assay. We found that 2.5 µM erastin markedly decreased cell viability; hence it was selected for follow-up experiments (Figure 2A). The cell viability was lower in Group E than in Group C, as revealed by the optical microscope and the trypan blue assay, which was increased by propofol pretreatment (Figures 2B,D,E). Similarly, the ROS, MDA, and iron levels were higher in Group E than in Group C as revealed by DHE staining, MDA, and iron test. All of these parameters were also reduced by propofol pretreatment (Figures 2C,F–H). The change of SOD activity was similar to the trypan blue results (Figure 2I). In addition, western bolt analysis showed a significant decrease in the expression of anti-ferroptosis enzymes (FTH1 and GPX4) in Group E compared with Group C, which was again reduced by propofol pretreatment (Figures 2J–L). Taken together, these results demonstrated that erastin induced ferroptosis in H9C2 cells and propofol pretreatment alleviates this form of death, suggesting an important protective role of propofol in the H9C2 cells ferroptosis.

### Effect of Propofol on AKT Knockdown in H9C2 Cells

**FIGURE 3:**
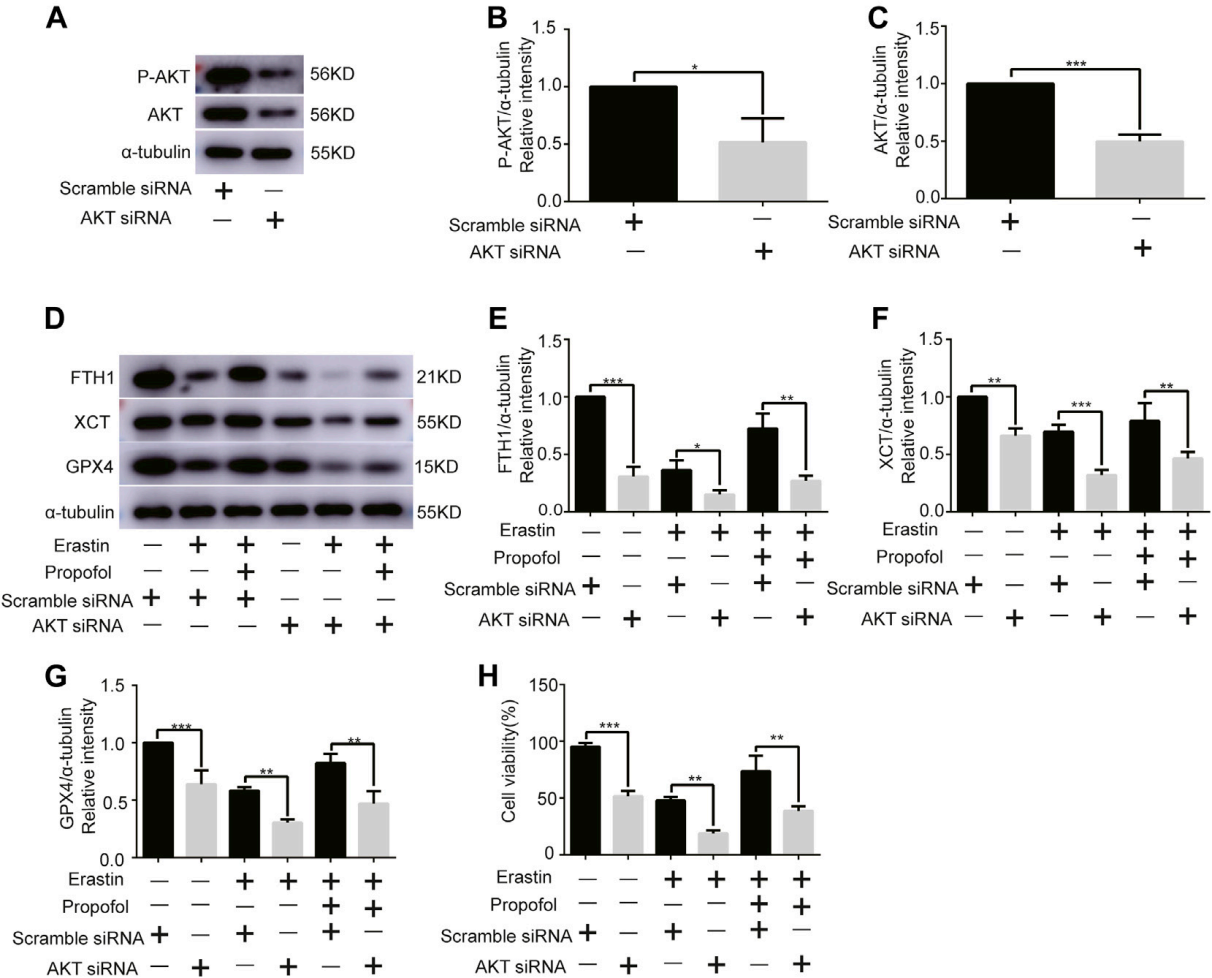
Effect of AKT knockdown on H9C2 cell ferroptosis with propofol pre-treatment. **(A–C)**. Expression of AKT and p-AKT in H9C2 cells transfected with AKT and scramble siRNA. **(D–G)**. Levels of FTH1, XCT, and GPX4 in H9C2 cells transfected with AKT and scramble siRNA with propofol pre-treatment. **(H)**. Change in the viability of H9C2 cells transfected with AKT and scramble siRNA with propofol pre-treatment. N = 3. Data are expressed as the mean ± SD. Significance was calculated using one-way ANOVA with Tukey’s post hoc test or the *t*-test. *p*-values < 0.05 were considered statistically significant. *p < 0.05, **p < 0.01, ***p < 0.001.

Given the central role of AKT in myocardial protection, we investigated whether AKT was involved in the anti-ferroptosis effect of propofol. We first knocked out the AKT gene of H9C2 cells with AKT siRNA. Western bolt analysis showed that AKT siRNA significantly reduced AKT and P-AKT expressions levels (Figures 3A–C). In addition, it showed that AKT siRNA significantly reduced the expression of anti-ferroptosis enzymes (FTH1, XCT, and GPX4) in H9C2 cells, which was further reduced by erastin, while propofol slightly inhibited the effect of erastin (Figures 3D–G). Similarly, the cell viability of H9C2 cells in the CCK8 experiment was similar to the above enzymes (Figure 3H). These results indicated that propofol might inhibit ferroptosis in H9C2 cells through the AKT signaling pathway.

### Influence of I/R-Induced Myocardial Ferroptosis

**FIGURE 4:**
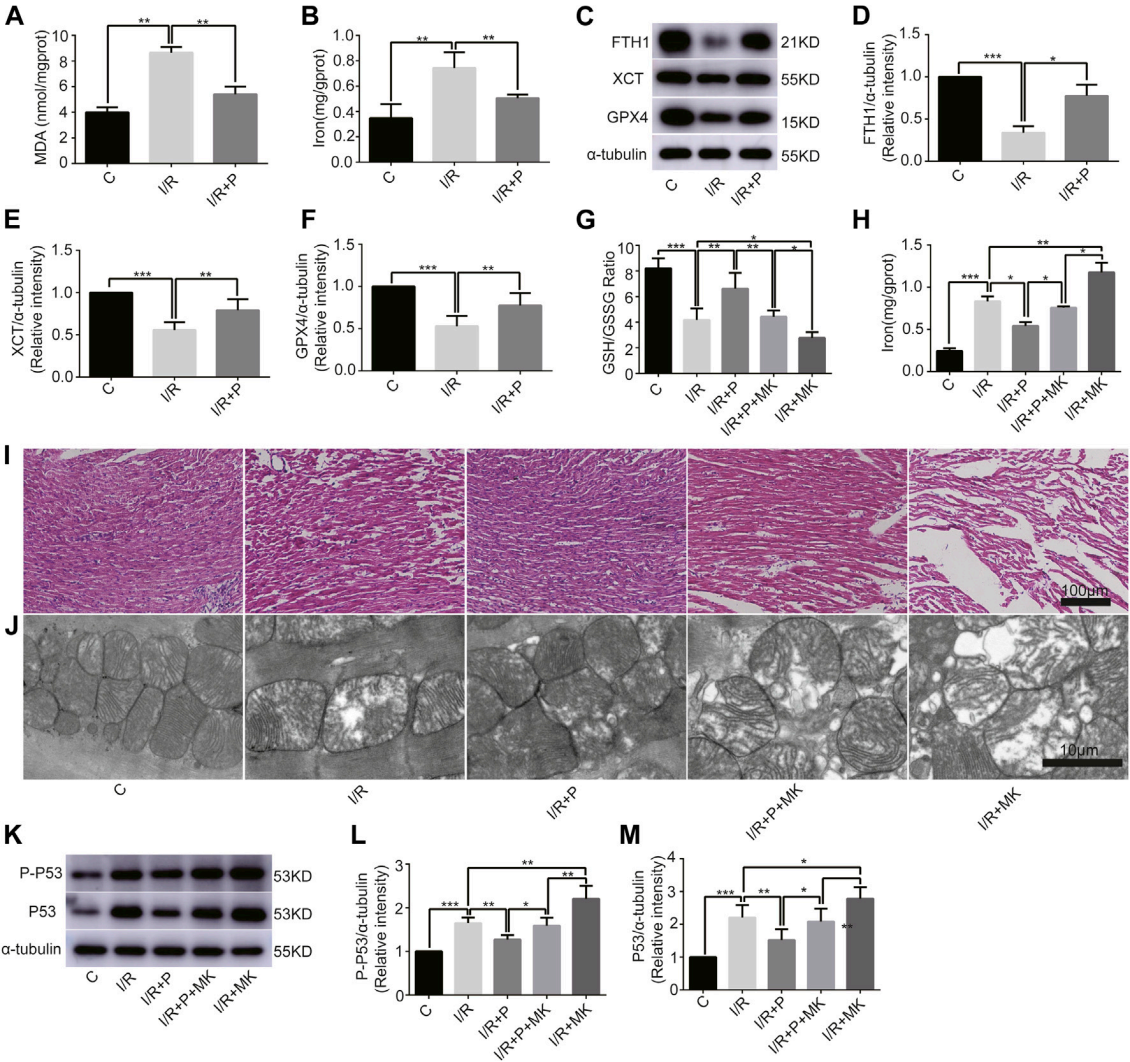
Influence of AKT inhibitor on myocardial ferroptosis with propofol pretreatment. **(A–F)**: Effect of propofol on I/R-induced myocardial MDA, iron, and antioxidant enzymes expressions. **(G–H)**: GSH/GSSG ratio and iron changes in the myocardium. **(I)** Structural changes in myocardial fibers based on HE staining. Scale bar: 100 μm. **(J)** Mitochondrial changes under electron microscopy. Scale bar: 10 μm. **(K–M)**: Expression of p53 and p-p53 in myocardium based on the western blot assay. N = 6. Data are expressed as the mean ± SD. Significance was calculated using one-way ANOVA with Tukey’s post hoc test or the *t*-test. *p*-values < 0.05 were considered statistically significant. *p < 0.05, **p < 0.01, ***p < 0.001.

To understand the effect of propofol on myocardial tissue ferroptosis, we established an isolated myocardial I/R model with the Langendorff system. Similarly, MDA and iron levels were higher in Group I/R than in Group C, as revealed by the MDA and iron test; they were also reduced by propofol pretreatment (Figures 4A,B). In addition, western bolt analysis revealed a significant decrease in the expression of anti-ferroptosis enzymes (FTH1, XCT, and GPX4) in the I/R compared with Group C, which was also reduced by propofol pretreatment (Figures 4C–F). Taken together, these results demonstrated that I/R induced myocardial tissue ferroptosis and propofol pretreatment alleviates this phenomenon.

### Influence of AKT Inhibitor on Myocardium Ferroptosis With Propofol Pre-Treatment

**FIGURE 5:**
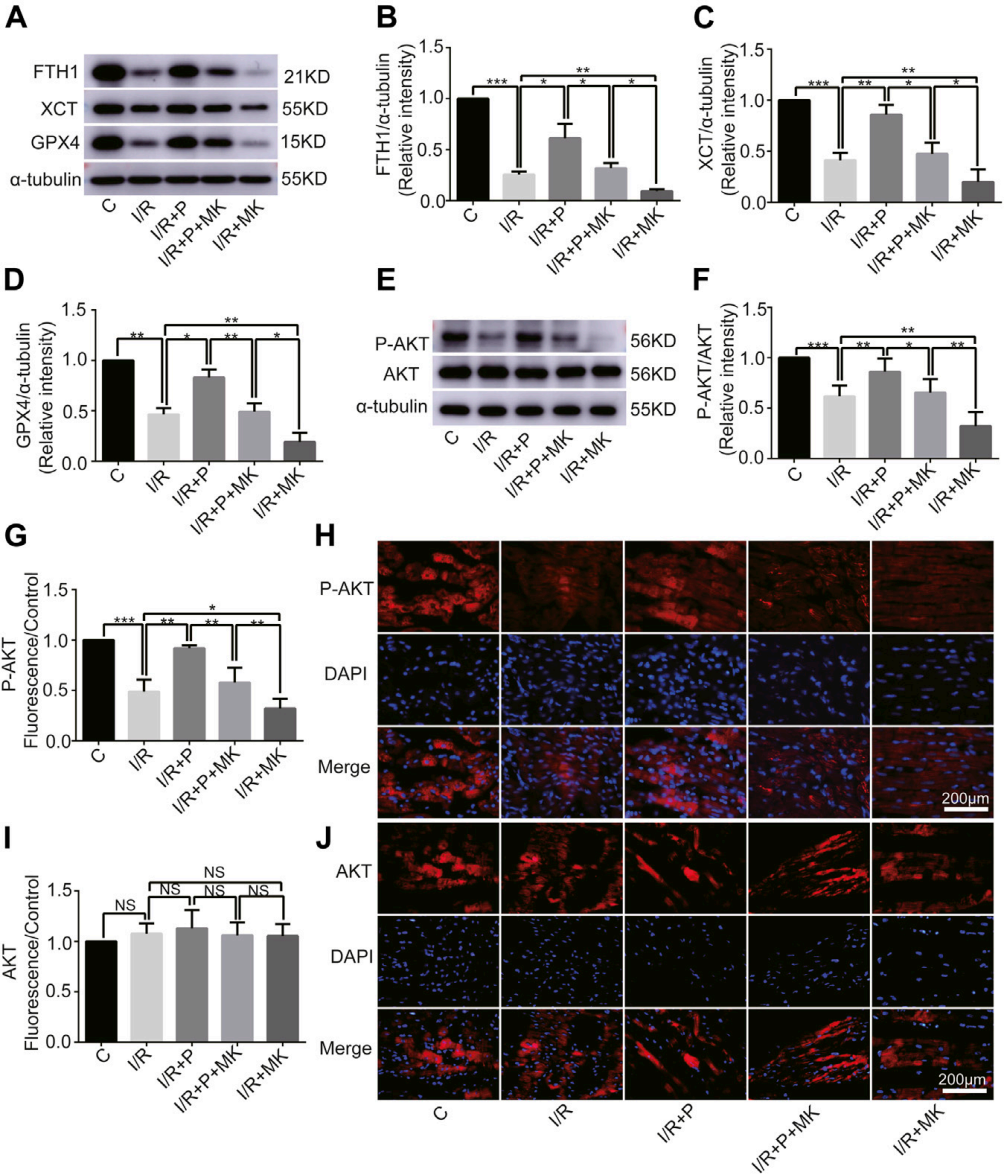
Changes in protein levels in response to propofol. **(A–D)**: Influence of propofol on I/R-induced antioxidant enzyme expression. **(E–F)**. Expression of AKT and p-AKT in myocardium based on western blot analysis. **(G–J)**. Expression of AKT and P-AKT by immunofluorescence. N = 6. Data are expressed as the mean ± SD. Scale bar: 20 µm. Significance was calculated using one-way ANOVA with Tukey’s post hoc test or the *t*-test. *p*-values < 0.05 were considered statistically significant. *p < 0.05, **p < 0.01, ***p < 0.001.

Next, we investigated whether AKT was involved in the anti-ferroptosis effect of propofol on the myocardium. We pretreated myocardial tissue with AKT inhibitor (MK2206). I/R showed a lower GSH/GSSG ratio than C, which was reversed with propofol. However, MK2206 inhibited all the effects of propofol (Figure 4G). MK2206 significantly increased myocardium iron levels in Group I/R + MK compared with Group I/R and inhibited the effect of propofol (Figure 4H). Similarly, HE staining showed an abundance of severely wavy and injured myofibers in Group I/R compared with Group C, which was prevented by propofol (Figure 4I). TEM revealed that enlarging or distorting myocardial mitochondrial ridges in Group I/R compared with Group C, which was significantly diminished in Group I/R + P. MK2206 attenuated all these effects (Figure 4J). Changes in p53 and p-p53 protein were common with increased myocardium iron (Figure 4K–M). In addition, western blot showed that the changes in anti-ferroptosis enzymes (FTH1, XCT, and GPX4) were similar to those in the GSH/GSSG ratio (Figures 5A–D). These results suggest that propofol pretreatment inhibits ferroptosis in myocardial tissue through the AKT/p53 signaling pathway.

### Change of AKT Activity by Propofol

Western blot analysis showed that AKT phosphorylation, which was inhibited by I/R or MK2206, was activated by propofol (Figures 5E,F). Immunofluorescence levels showing P-AKT phosphorylation were identical to the western blot results shown above (Figures 5G,H). There was no difference in immunofluorescence levels of AKT among the groups (Figures 5I,J).

## Discussion

The major finding of this study was that propofol pretreatment inhibited ferroptosis in H9C2 cells and rat myocardium by reducing the expression of ROS, iron, and lipid peroxidation as well as increasing the expression of anti-ferroptosis enzymes. Therefore, propofol protects the myocardium from I/R injury through the AKT/P53 signaling pathway. To the best of our knowledge, this is the first study demonstrating that propofol inhibits myocardial ferroptosis and the underlying molecular mechanism.

Investigating the death processes and underlying mechanisms of cardiomyocytes may provide insights into novel therapeutic approaches for heart diseases, especially I/R injury (Whelan et al., 2010). Ferroptosis, which is distinct from conventional cell death, including apoptosis, necroptosis, and autophagy-dependent cell death, has recently been identified as a key pathological process connecting oxidative stress, inflammation, and cardiovascular diseases (Yu et al., 2021). Ferroptosis, an iron-dependent type of cell death that is mainly caused by oxidation-reduction imbalance, differs from conventional cell death processes characterized by typical cell shrinkage, mitochondrial fragmentation, and nuclear condensation. With the increasing understanding of the molecular mechanisms of ferroptosis, ferroptosis has become a potential novel diagnostic and therapeutic target of cardiomyopathy. In this study, our results demonstrated that ferroptosis played an essential role in myocardial injury induced by erastin or I/R following previous findings (Fang et al., 2019; Li et al., 2020). Briefly, erastin or I/R triggered iron accumulation and then damaged cardiomyocytes due to oxidative stress (Olivieri, 1994).

A number of studies have reported that propofol protects the myocardium from I/R injury (Kobayashi et al., 2008; Li et al., 2019), but the mechanism is not clear. Our study showed that p53, which promoted fatal lipid ROS accumulation and resulted in cardiomyocyte’s ferroptosis, was upregulated in I/R-induced myocardial injury, which is consistent with the previous study (Tang et al., 2021). Notably, we found that propofol, which significantly inhibited p53 expression, inhibited myocardial ferroptosis caused by erastin or I/R.

Our results also demonstrated that erastin or I/R induced antioxidant enzyme damage and ROS accumulation, resulting in cell ferroptosis. Recent studies have shown that GPX4 improves iron absorption by directly decomposing peroxides, catalyzing the transformation of reduced glutathione (GSH) to oxidized GSH (GSSG) (Imai et al., 2017), and inhibiting the expression of ferritin heavy chain 1 (FTH1) (Sun et al., 2016). In the present study, we found that propofol improved the antioxidant capacity of cardiomyocytes by improving the levels of FTH1, XCT, or GPX4 and reducing iron levels. Another study has shown that p53 inhibits cysteine absorption by downregulating XCT expression (Magri et al., 2021), resulting in the inhibition of cystine-dependent glutathione oxidase activity and increased cell lipid ROS, leading to ferroptosis (Jiang et al., 2015). Our results showing propofol downregulation of p53 and reduction of cardiomyocyte dysfunction are consistent with the previous study (Lai et al., 2011).

**FIGURE 6:**
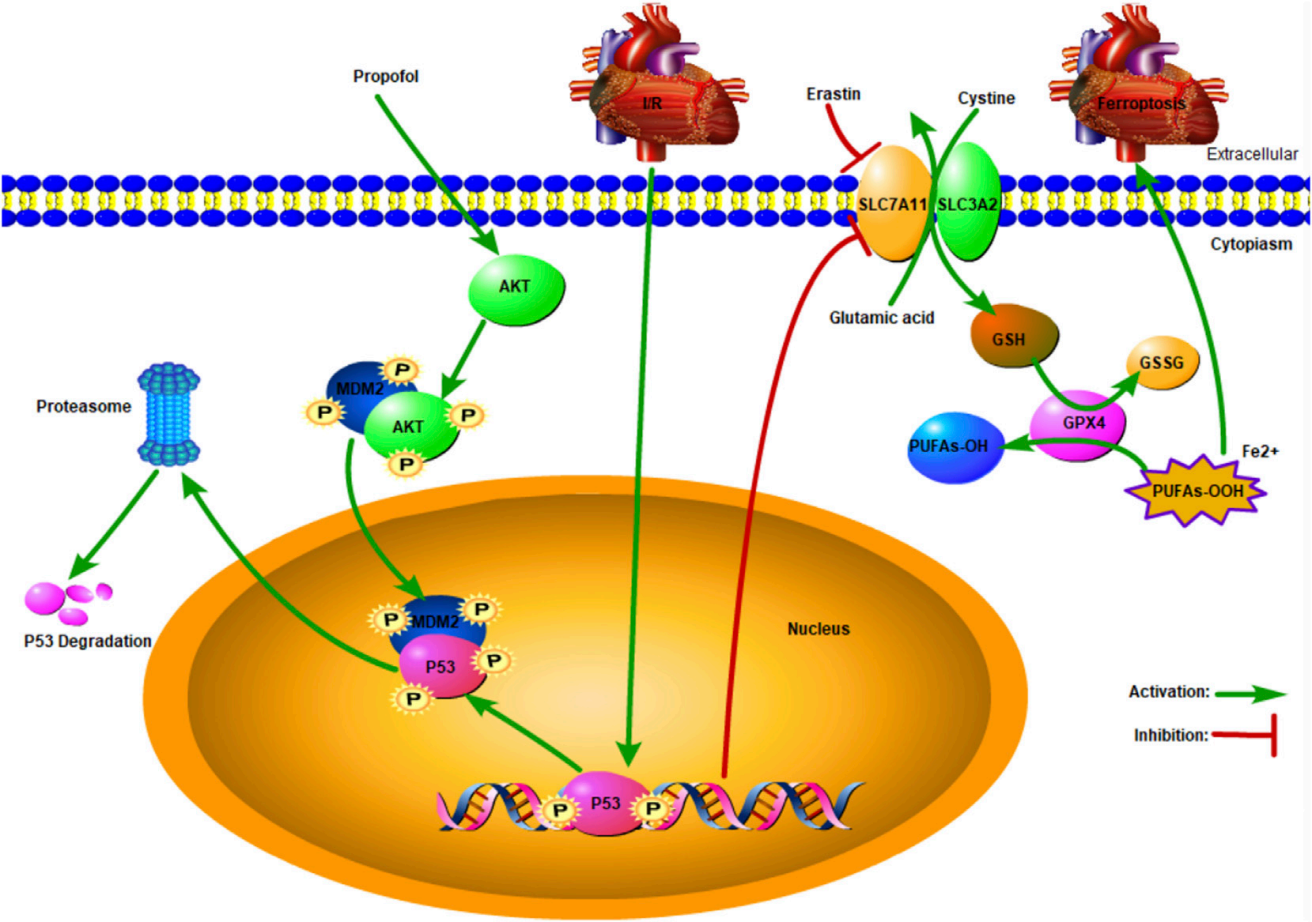
Schematic: Propofol protects the myocardium from ischemia/reperfusion injury by influencing an AKT/p53 signaling pathway-mediated ferroptosis mechanism.

A recent study showed that AKT plays a pivotal role in p53 degradation (Chibaya et al., 2021), which is inconsistent with the present findings. The p53 was degraded by binding with MDM2 (Wang et al., 2020), and this process was accelerated by AKT phosphorylation at ser166 or 188 (Feng et al., 2004). As shown in our study, propofol protected the myocardium from I/R-induced injury by AKT phosphorylation (Shravah et al., 2014). The expression of p53 increased after MK2206 pretreatment, indicating that signaling pathways other than AKT were involved in p53 degradation. When DNA is damaged, ataxia telangiectasia-mutated (ATM) activation induces p53 activation, resulting in cell aging or apoptosis (Jiang and Chen, 2020). Calcium-dependent kinase 5 (CDK5) and CDK9 activate p53 by phosphorylating ser33, which enhances the binding abilities of cyclic adenosine monophosphate (AMP) response element-binding protein (CREB) binding protein (CBP), thus raising p53 transcription activity (Zhang et al., 2002) (Figure 6).

Our study has some limitations. First, the Langendorff model rather than the rat I/R model was used in this study. However, the rat Langendorff model is easy to prepare, and the experimental results are reproducible (Finegan et al., 2000), which is consistent with best practices for reduction fine displacement (3R) (Flecknell, 2002). Second, the protective effects of AKT, which is the key survival signaling component in the heart, are associated with numerous factors. Recent studies have shown that propofol plays a protective role in the heart by activating the AKT/eNOS (Yan et al., 2021), ser473/thr308 (Lou et al., 2015), or PI3K/AKT (Wu et al., 2012) signal pathways. In our study, we only explored one biological process of AKT; other regulatory effects require further investigation. Third, the animal experiments were complicated and difficult, with a low success rate and inconsistent results (De Villiers and Riley, 2020). Fourth, propofol was used at a single concentration (50 µM) according to a previous study (Yu et al., 2018), in which propofol (50 µM) pretreatment was able to protect myocardium from I/R injury in the rat Langendorff model. The single concentration of propofol used in this study could limit our understanding of its role in I/R injury. Finally, we measured only the expression levels of FTH1, XCT, and GPX4 but not those of other antioxidant enzymes.

In conclusion, propofol pretreatment inhibits myocardial ferroptosis through the AKT/p53 signaling pathway, reducing ROS, iron, and lipid peroxidation and increasing antioxidant enzyme expression. These data may explain why propofol reduces perioperative complications associated with myocardial I/R injury, including arrhythmias, decreasing systolic and diastolic function, and myocardial stunning.

## Data Availability

The original contributions presented in the study are included in the article/Supplementary Material, further inquiries can be directed to the corresponding authors.
