# Corrigendum: Propofol Protects Myocardium From Ischemia/Reperfusion Injury by Inhibiting Ferroptosis Through the AKT/p53 Signaling Pathway

**DOI:** 10.3389/fphar.2022.910421

**Published:** 2022-04-28

**Authors:** Shengqiang Li, Zhen Lei, Xiaomei Yang, Meng Zhao, Yonghao Hou, Di Wang, Shuhai Tang, Jingxin Li, Jingui Yu

**Affiliations:** ^1^ Department of Anesthesiology, Qilu Hospital, Cheeloo College of Medicine, Shandong University, Jinan, China; ^2^ Department of Anesthesiology, The Second Hospital, Cheeloo College of Medicine, Shandong University, Jinan, China; ^3^ Department of Physiology, School of Basic Medical Science, Cheeloo College of Medicine, Shandong University, Jinan, China

**Keywords:** Akt, ferroptosis, heart, ischemia/reperfusion injury, propofol, p53

In the published article, there was an error regarding the affiliation for Jingxin Li. The full designation for affiliation 3 is “Department of Physiology, School of Basic Medical Science, Cheeloo College of Medicine, Shandong University, Jinan, Shandong, China”.

There was an error in the Funding statement. The correct name for the third listed funder is “Key Research and Development Program of Shandong Province (No. 2018GSF118079)”.

The authors apologize for these errors and state that this does not change the scientific conclusions of the article in any way. The original article has been updated.

