# Supplementary material for: Propofol Protects Myocardium From Ischemia/Reperfusion Injury by Inhibiting Ferroptosis Through the AKT/p53 Signaling Pathway
Source: Front Pharmacol. 2022 Mar 16;13:841410. doi: 10.3389/fphar.2022.841410 (PMC8966655; doi:10.3389/fphar.2022.841410)

**Supplemental figure S1**: Full scan of the original blots of cropped images shown in Figure 3A.

Lane1 and lane4: marker. Lane2: Scramble siRNA. Lane3: AKT siRNA

P-AKT AKT


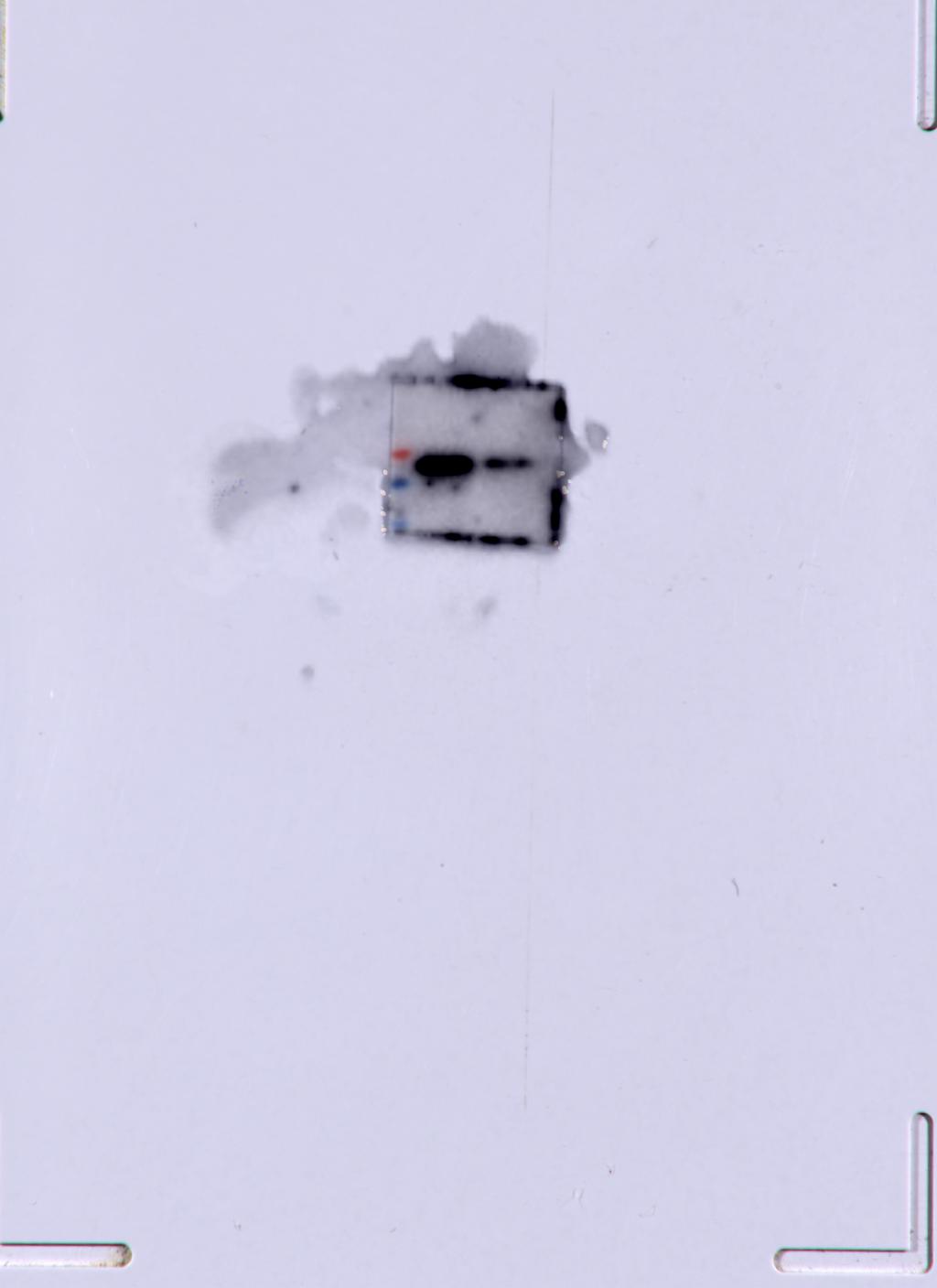

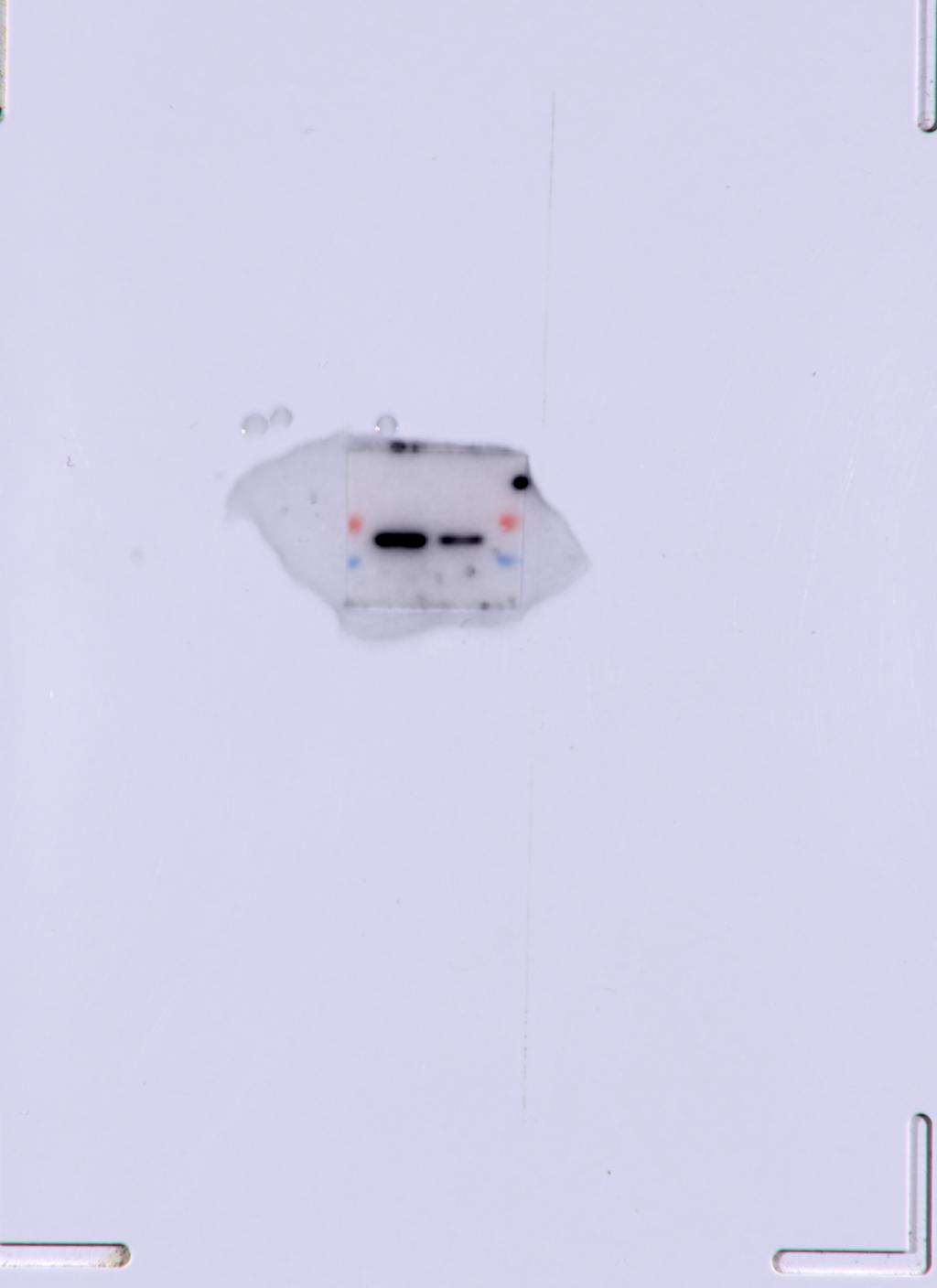


α-tubulin


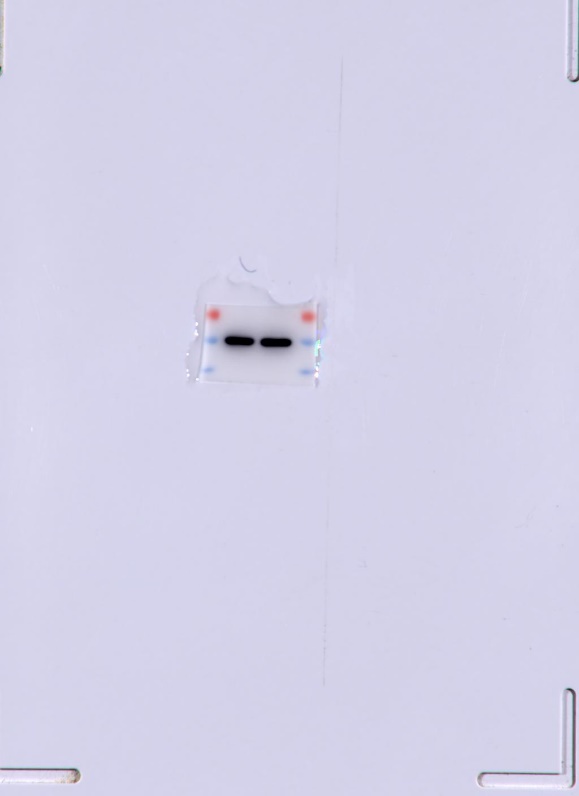


**Supplemental figure S2**: Full scan of the original blots of cropped images shown in Figure3D.

Lane1,8: marker. Lane2: Scramble C. Lane3: Scramble E. Lane4: Scramble E+P. Lane5: AKT C. Lane6: AKT E. Lane7: AKT E+P.

FTH1 XCT


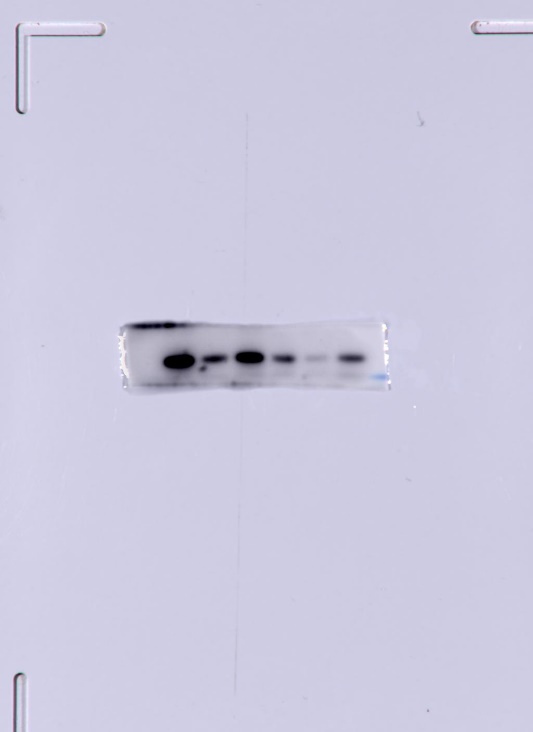

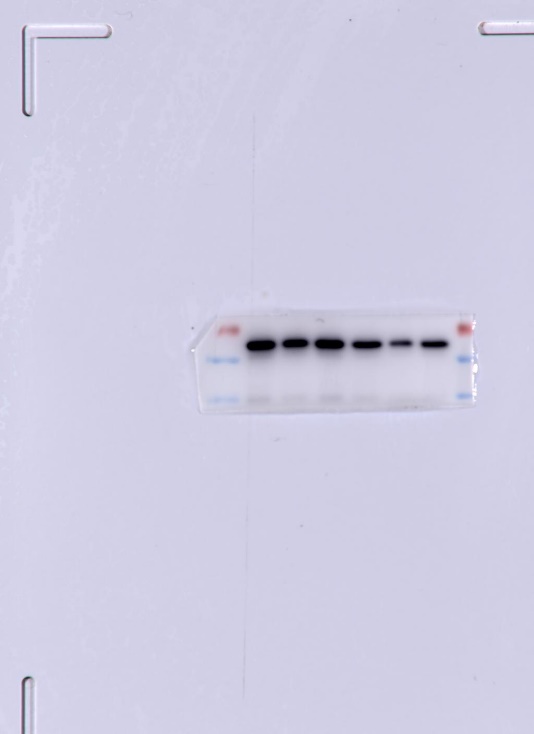


SOD-2 GPX4


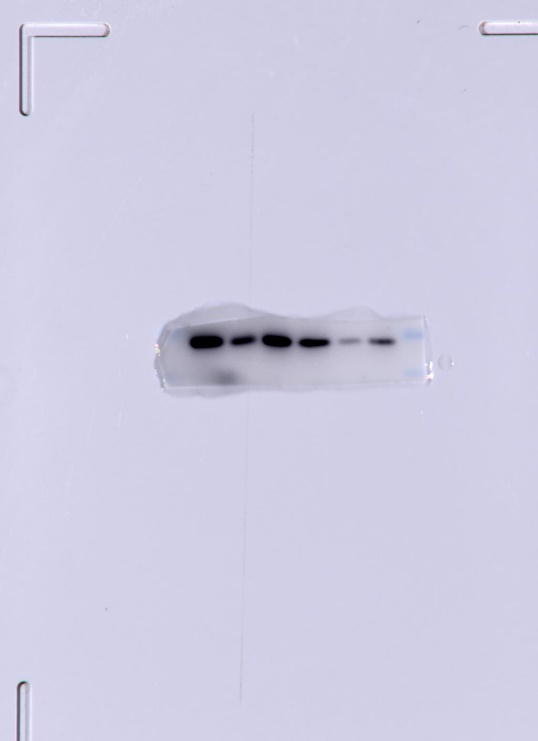

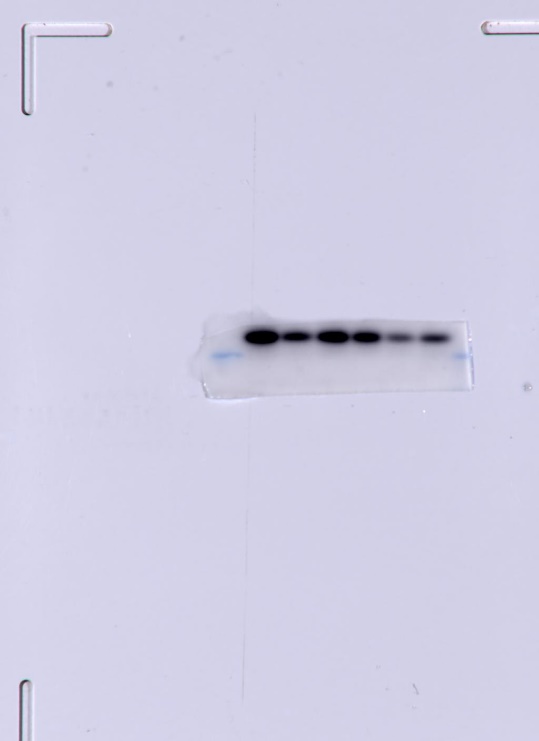


α-tubulin


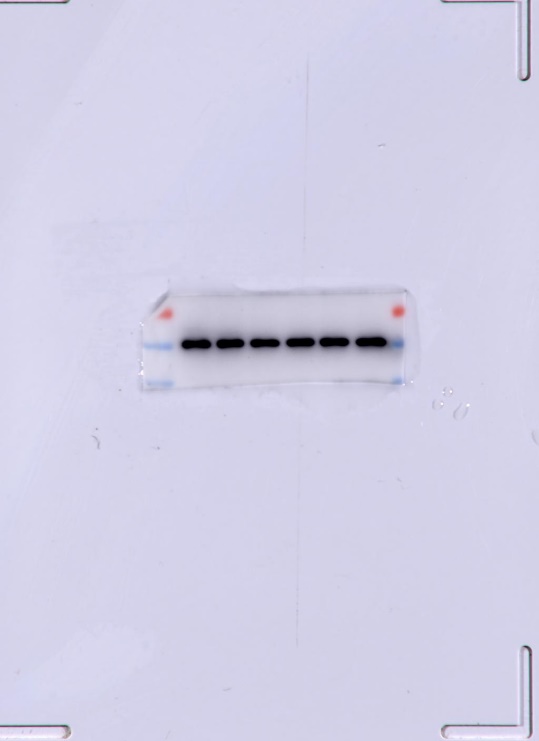

Supplement: Supplementary file 1 [file DataSheet3.ZIP › Fig3/Fig3A,D, WB.docx]
