# Supplementary figures and images for: Propofol Protects Myocardium From Ischemia/Reperfusion Injury by Inhibiting Ferroptosis Through the AKT/p53 Signaling Pathway
Source: Front Pharmacol. 2022 Mar 16;13:841410. doi: 10.3389/fphar.2022.841410 (PMC8966655; doi:10.3389/fphar.2022.841410)

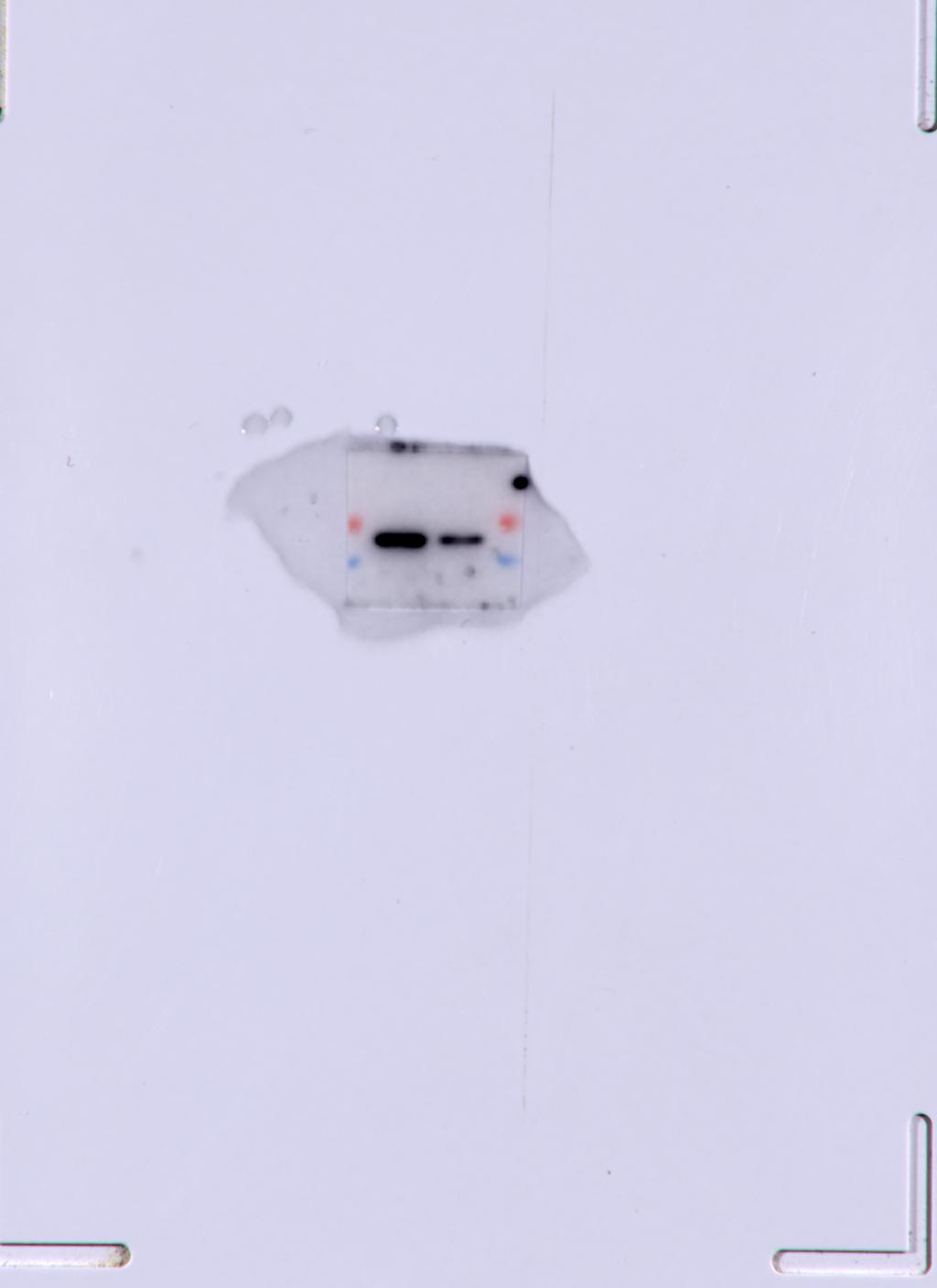

Supplement: Supplementary file 1 [file DataSheet3.ZIP › Fig3/Fig3A-C/AKT.jpg]

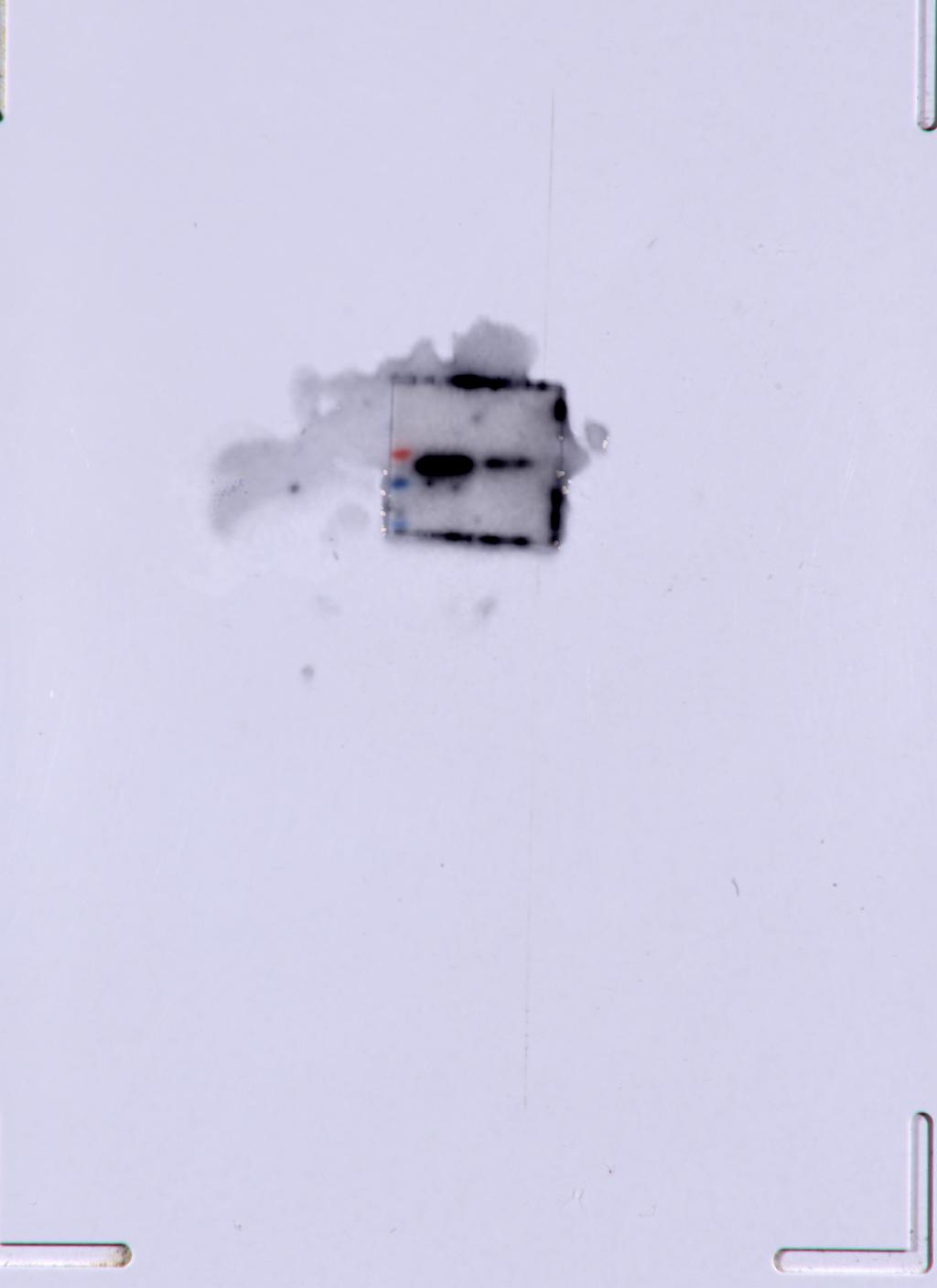

Supplement: Supplementary file 1 [file DataSheet3.ZIP › Fig3/Fig3A-C/P-AKT.jpg]

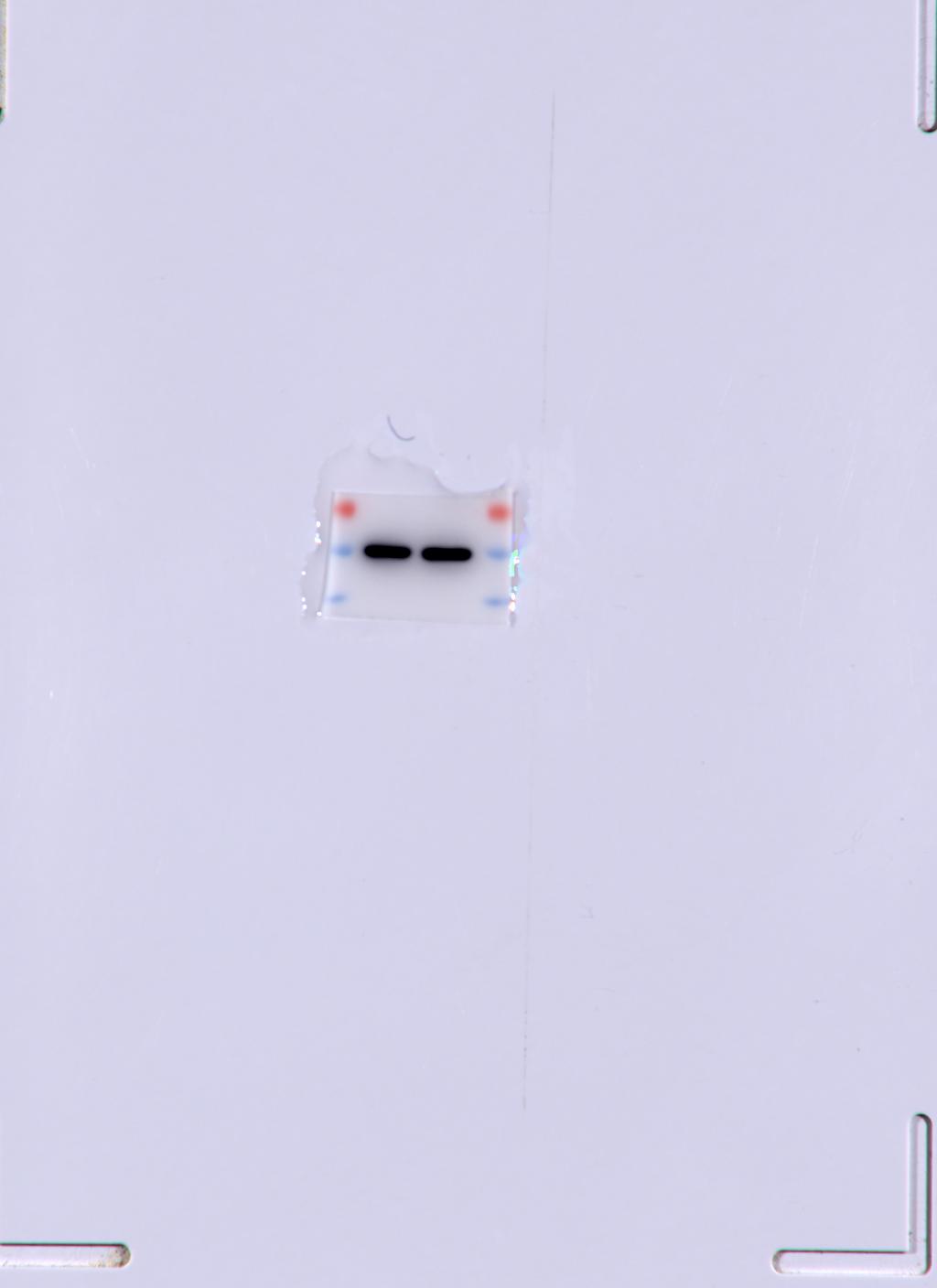

Supplement: Supplementary file 1 [file DataSheet3.ZIP › Fig3/Fig3A-C/α-tubulin.jpg]

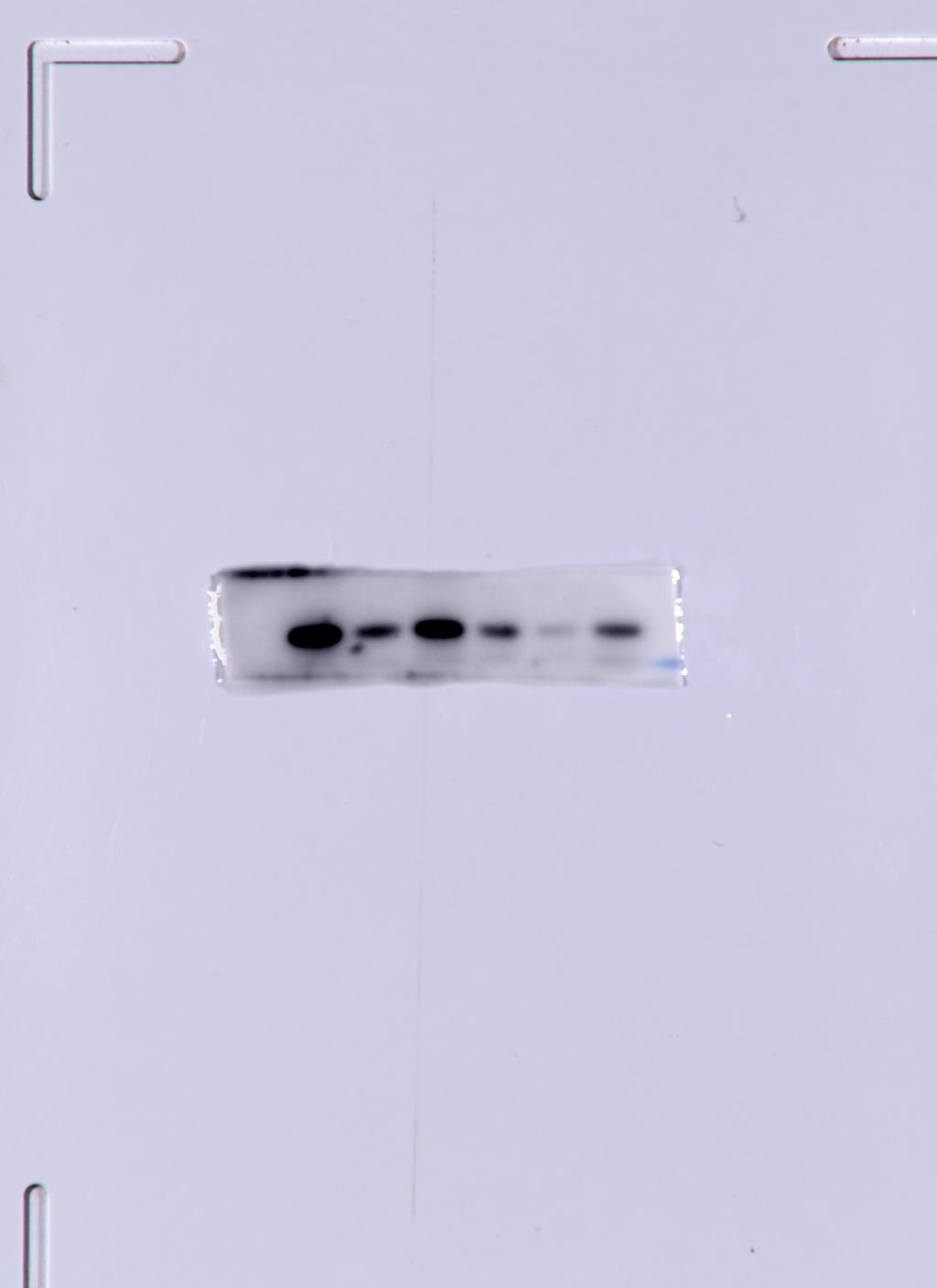

Supplement: Supplementary file 1 [file DataSheet3.ZIP › Fig3/Fig3D-H/FTH1.jpg]

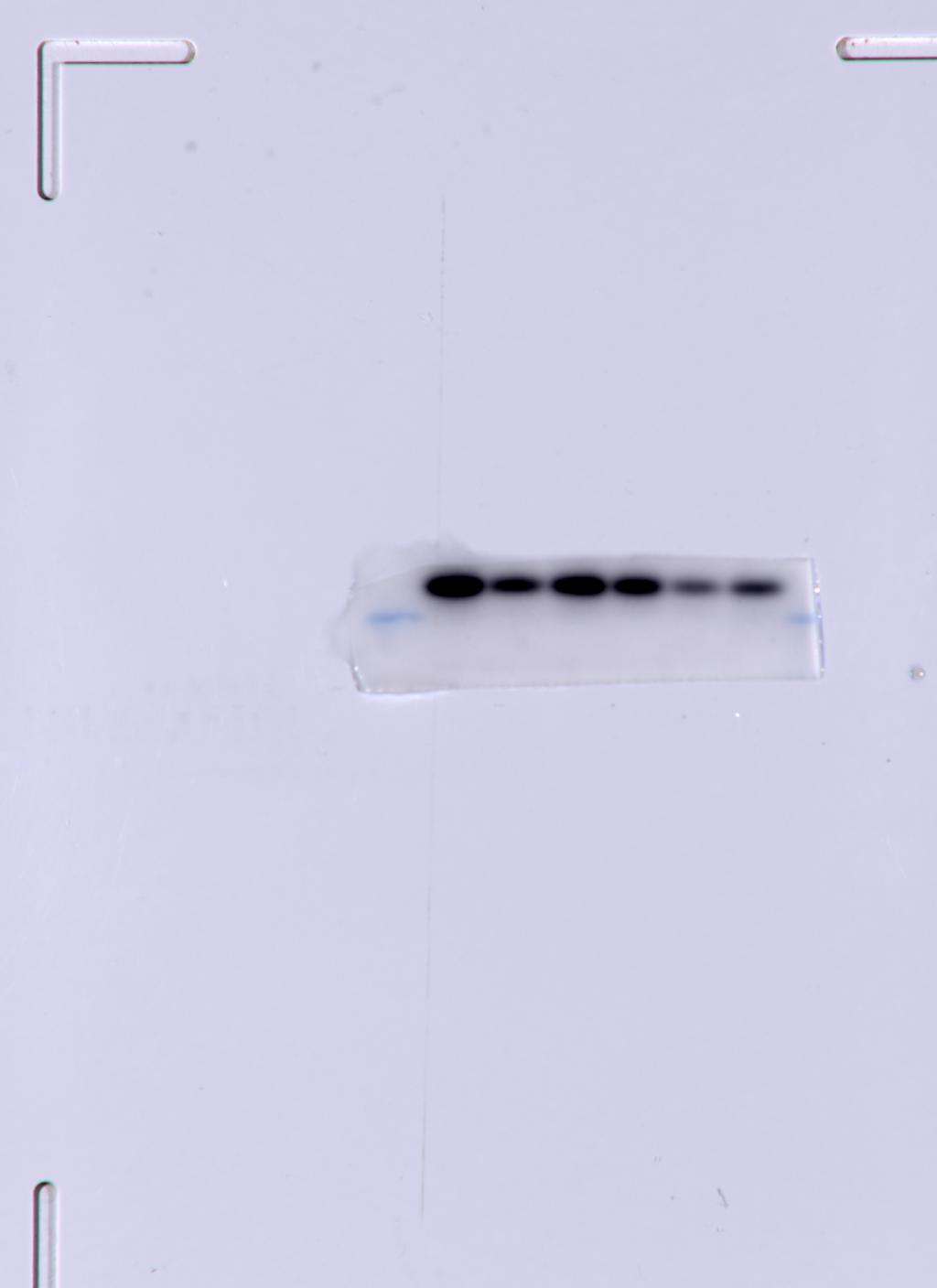

Supplement: Supplementary file 1 [file DataSheet3.ZIP › Fig3/Fig3D-H/GPX4.jpg]

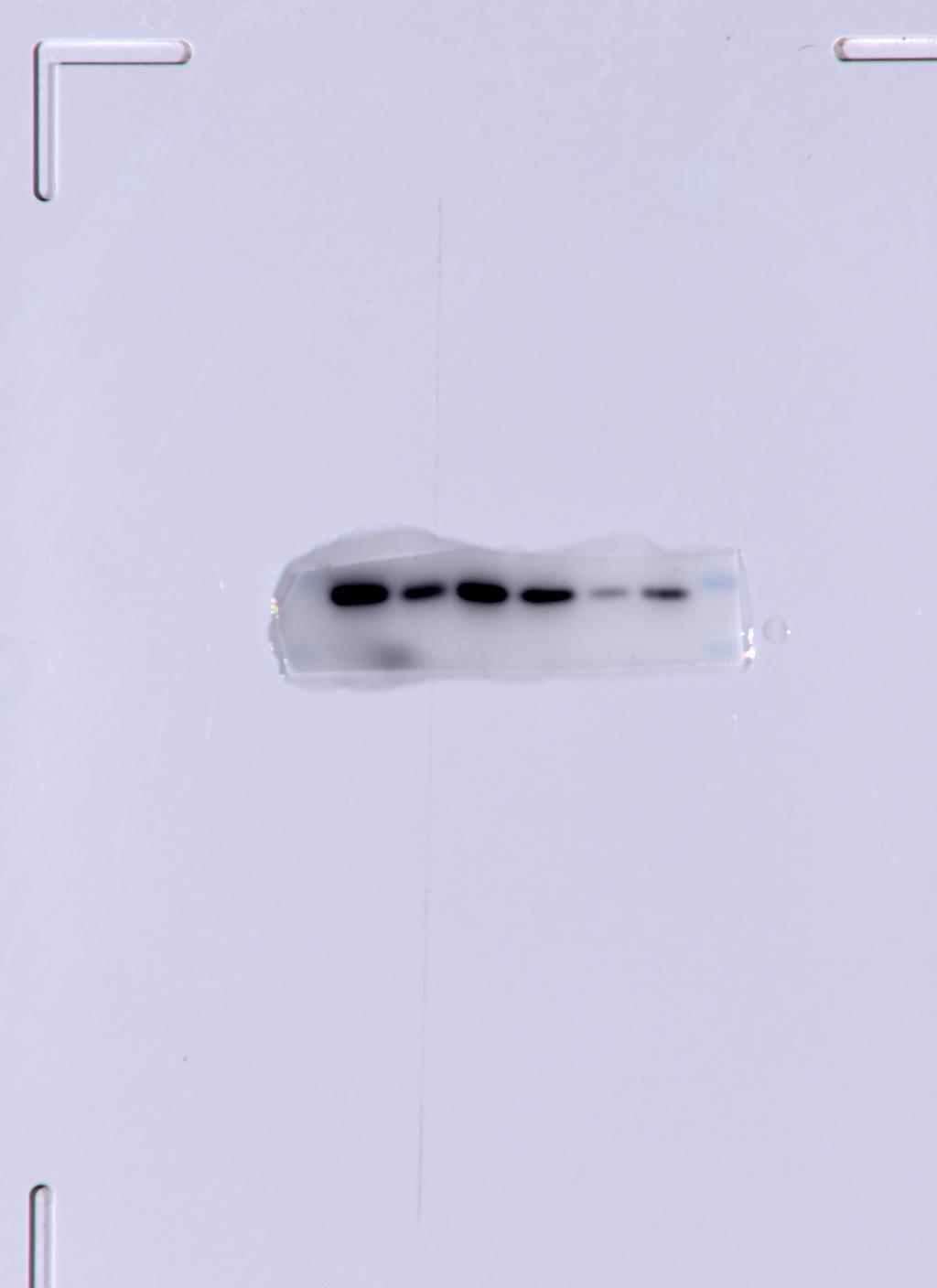

Supplement: Supplementary file 1 [file DataSheet3.ZIP › Fig3/Fig3D-H/SOD-2.jpg]

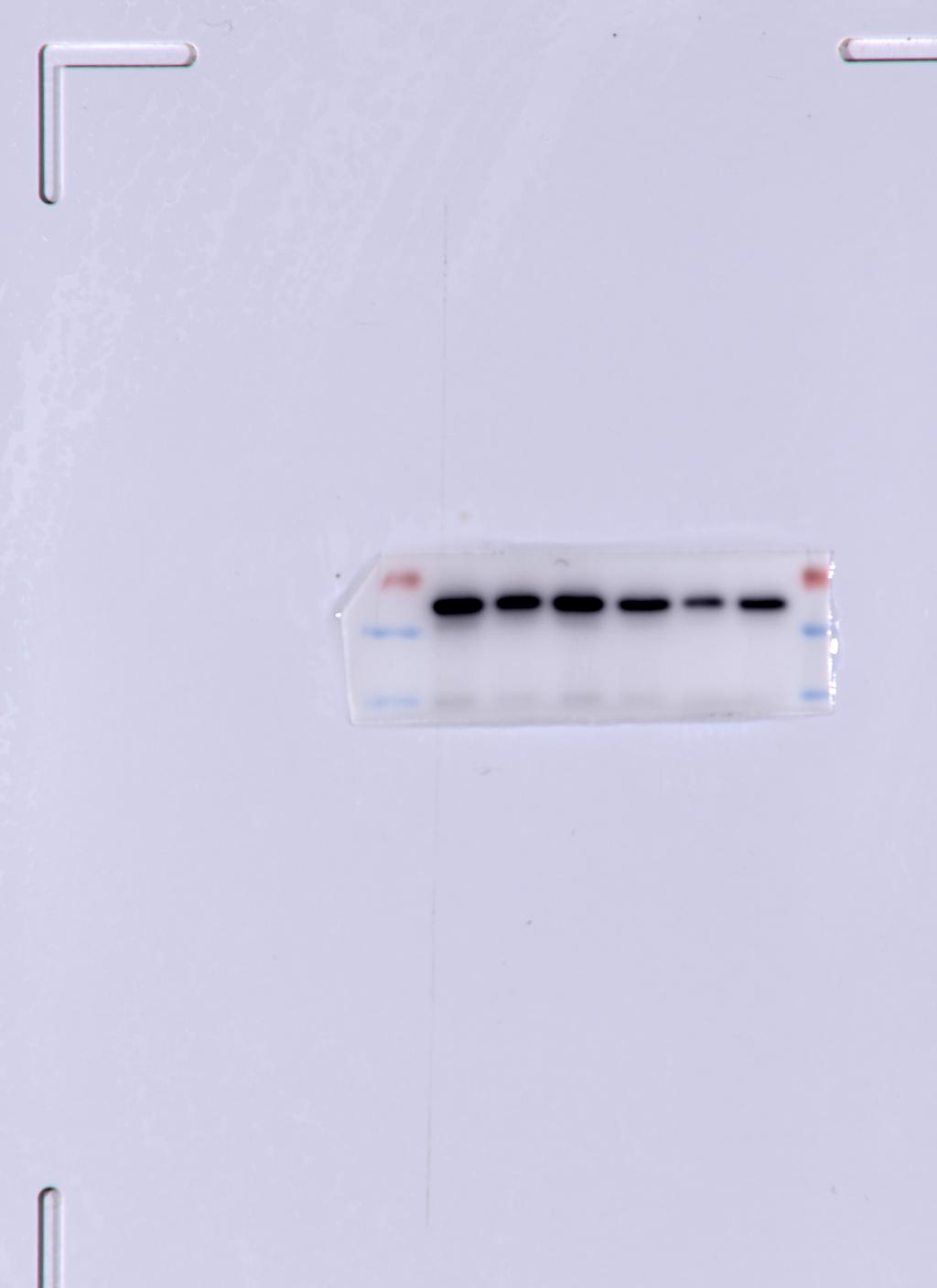

Supplement: Supplementary file 1 [file DataSheet3.ZIP › Fig3/Fig3D-H/XCT.jpg]

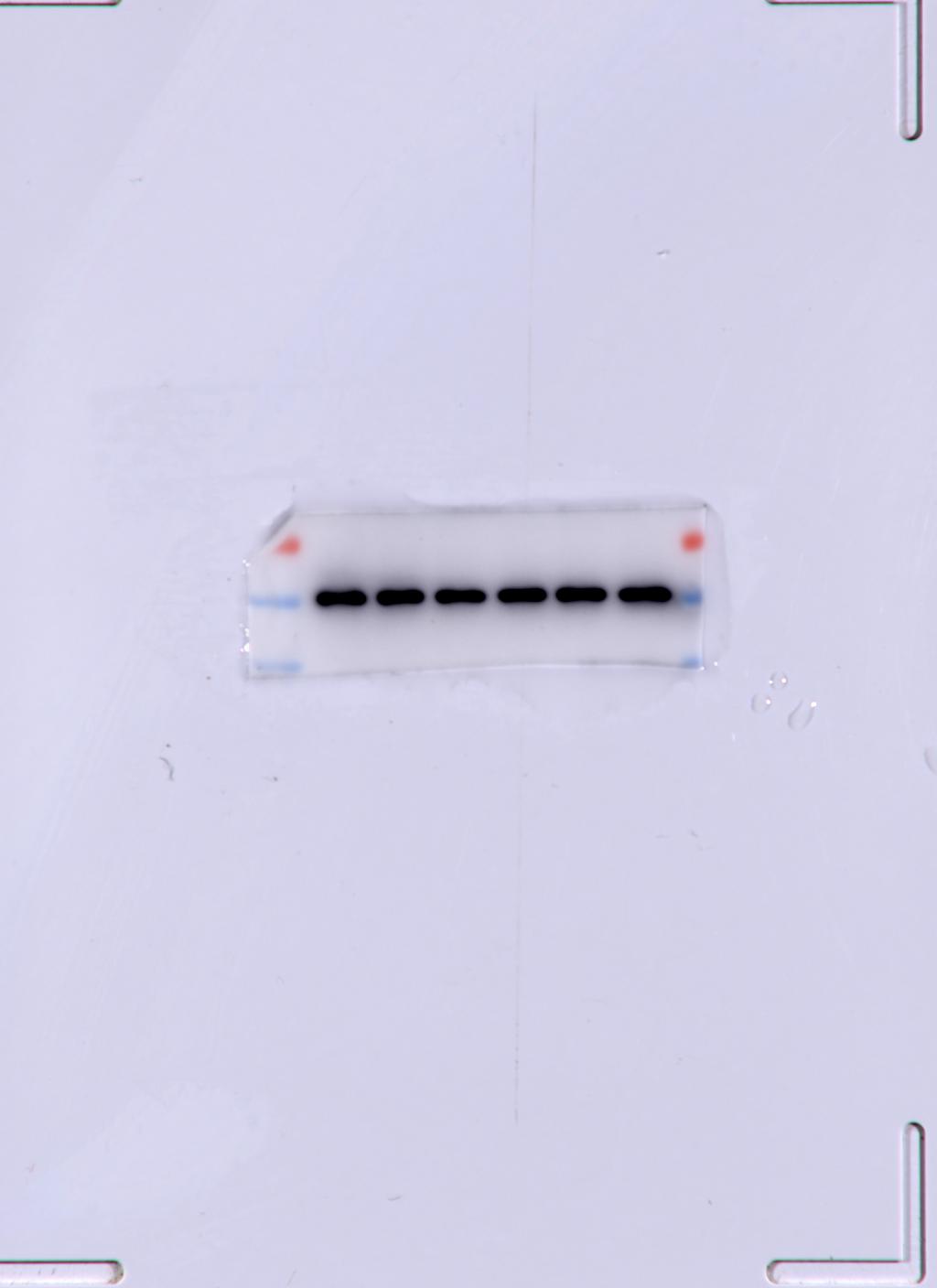

Supplement: Supplementary file 1 [file DataSheet3.ZIP › Fig3/Fig3D-H/α-tubulin.jpg]

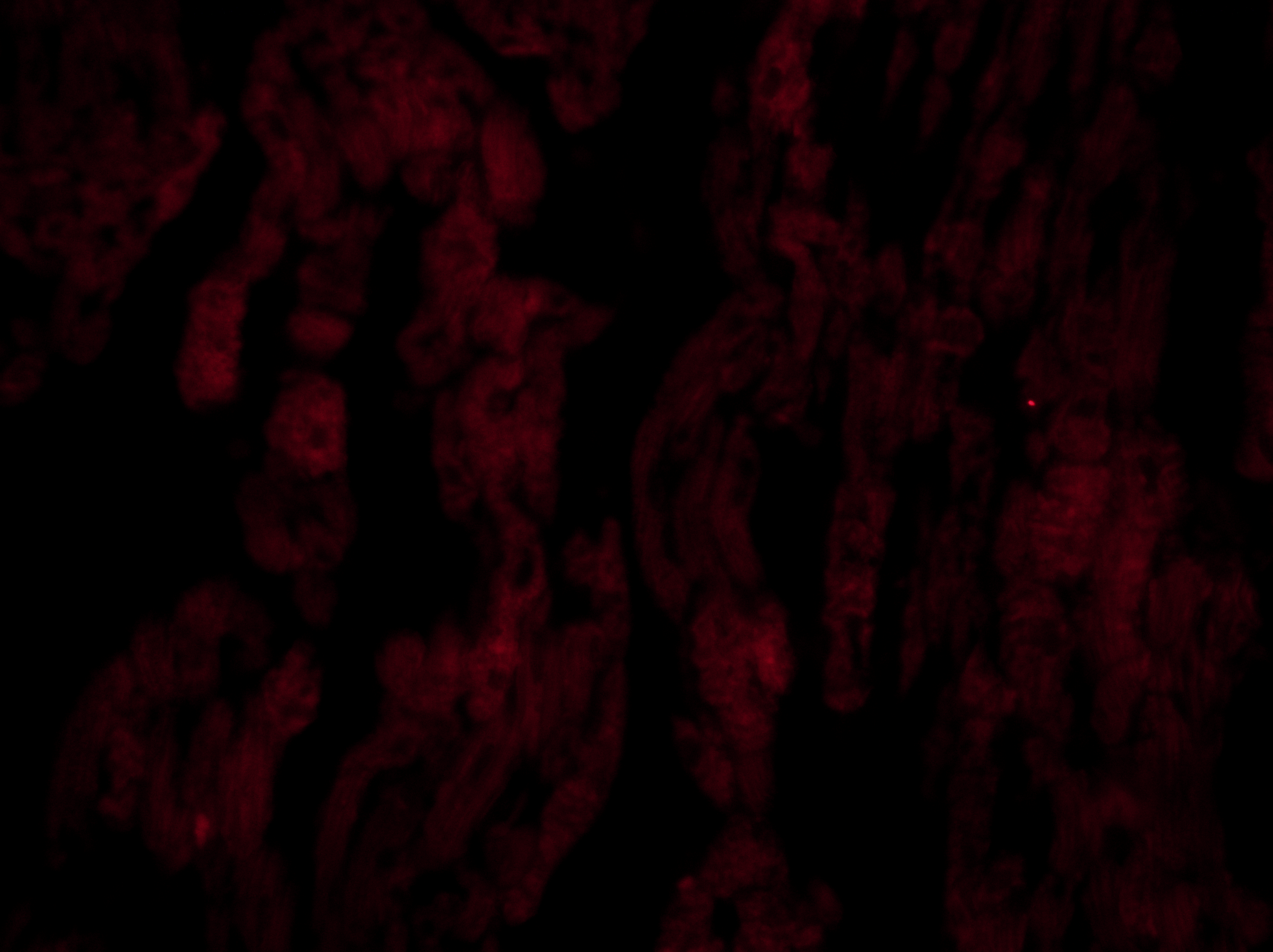

Supplement: Supplementary file 2 [file DataSheet14.ZIP › Fig5二2/Control P-AKT.tif]

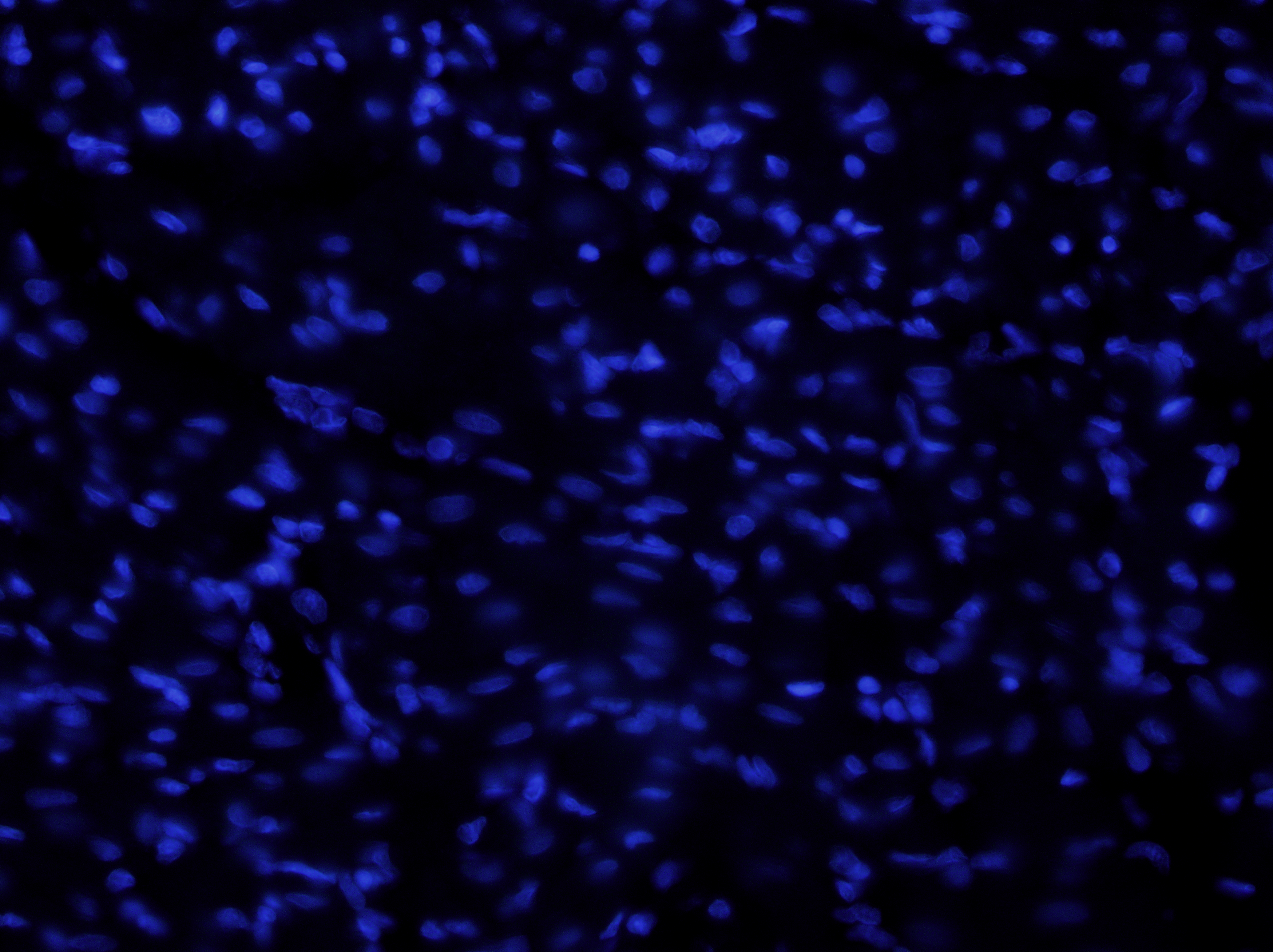

Supplement: Supplementary file 2 [file DataSheet14.ZIP › Fig5二2/IR dapi.tif]

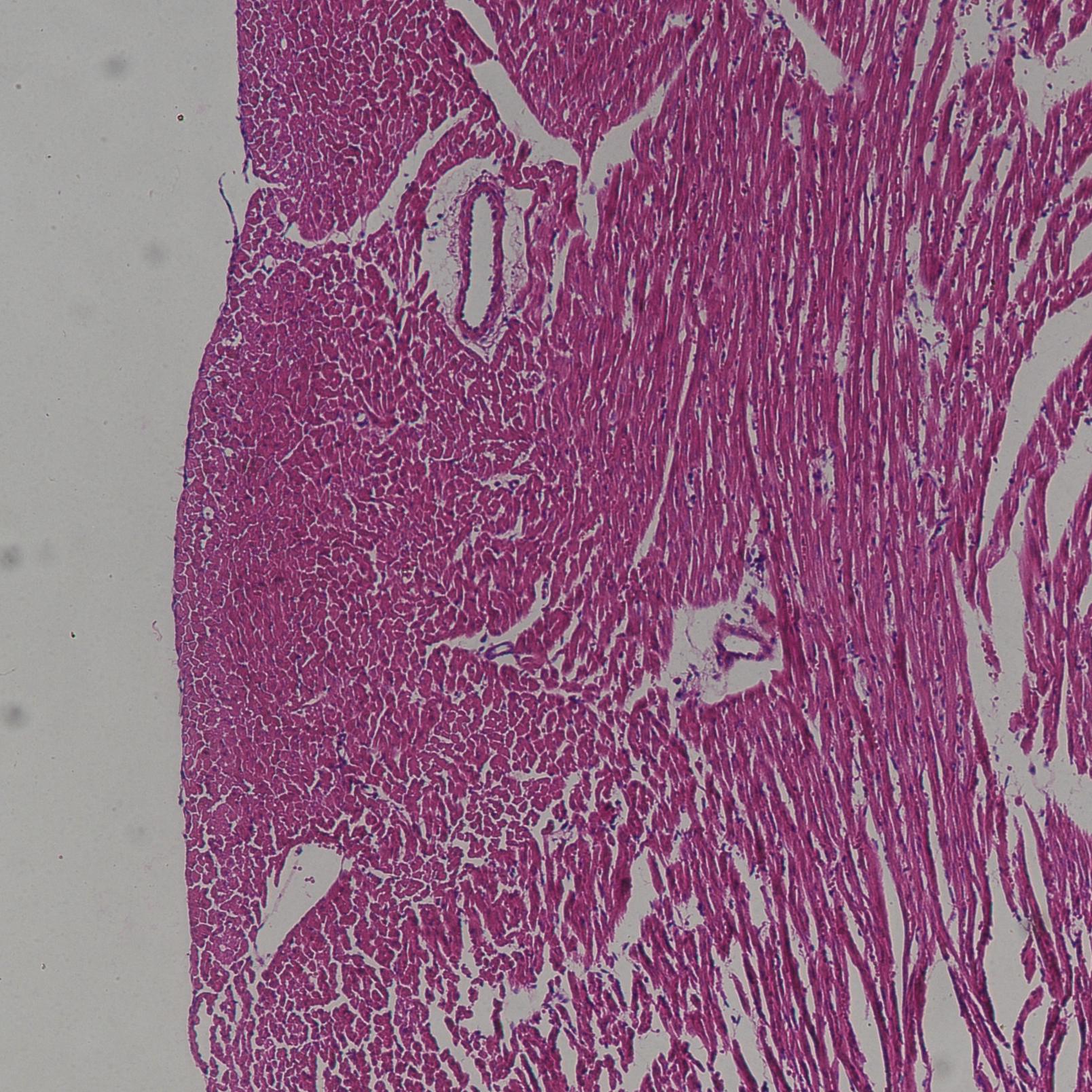

Supplement: Supplementary file 3 [file DataSheet11.ZIP › Fig4三2/IR+P+MK.tif]

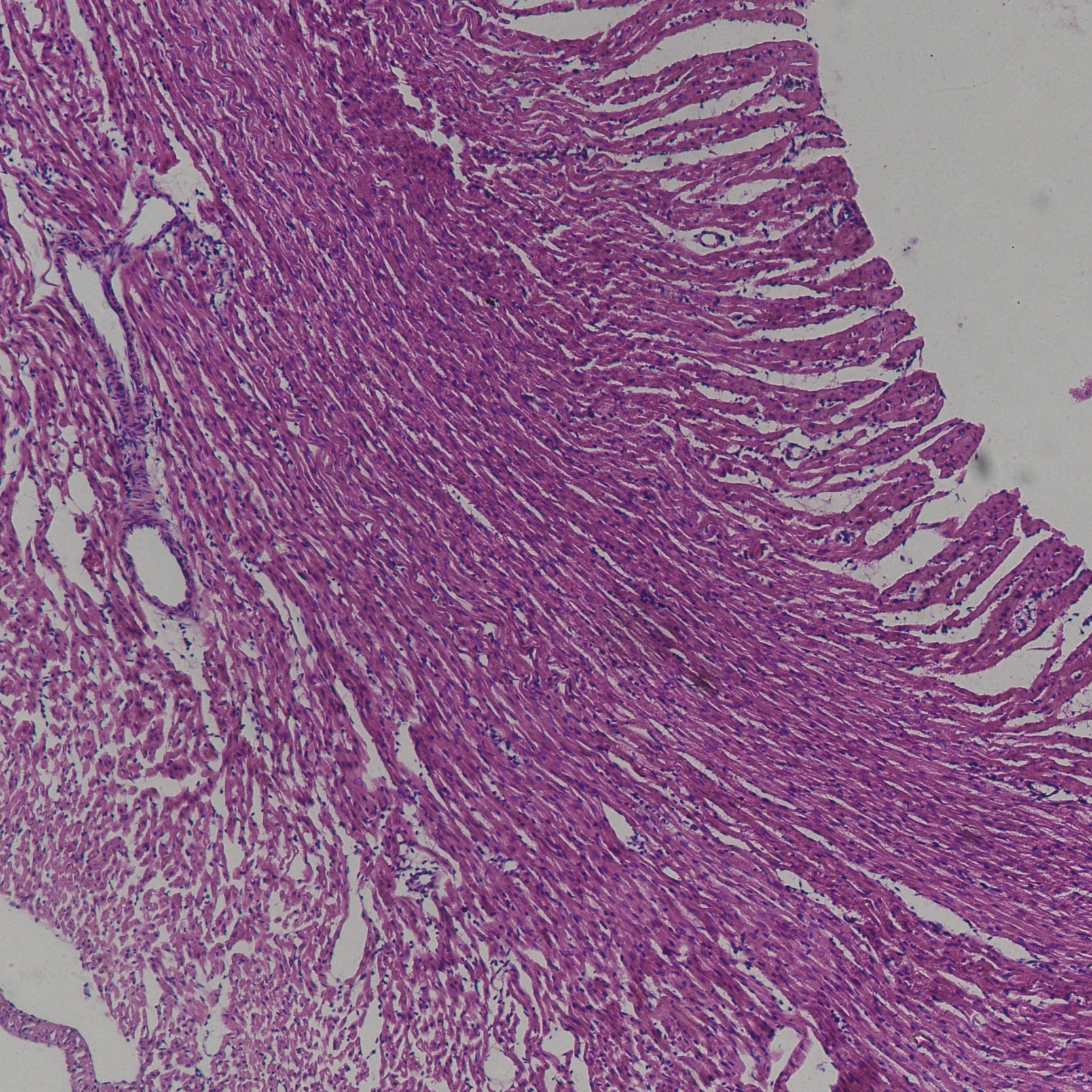

Supplement: Supplementary file 3 [file DataSheet11.ZIP › Fig4三2/IR+P.tif]

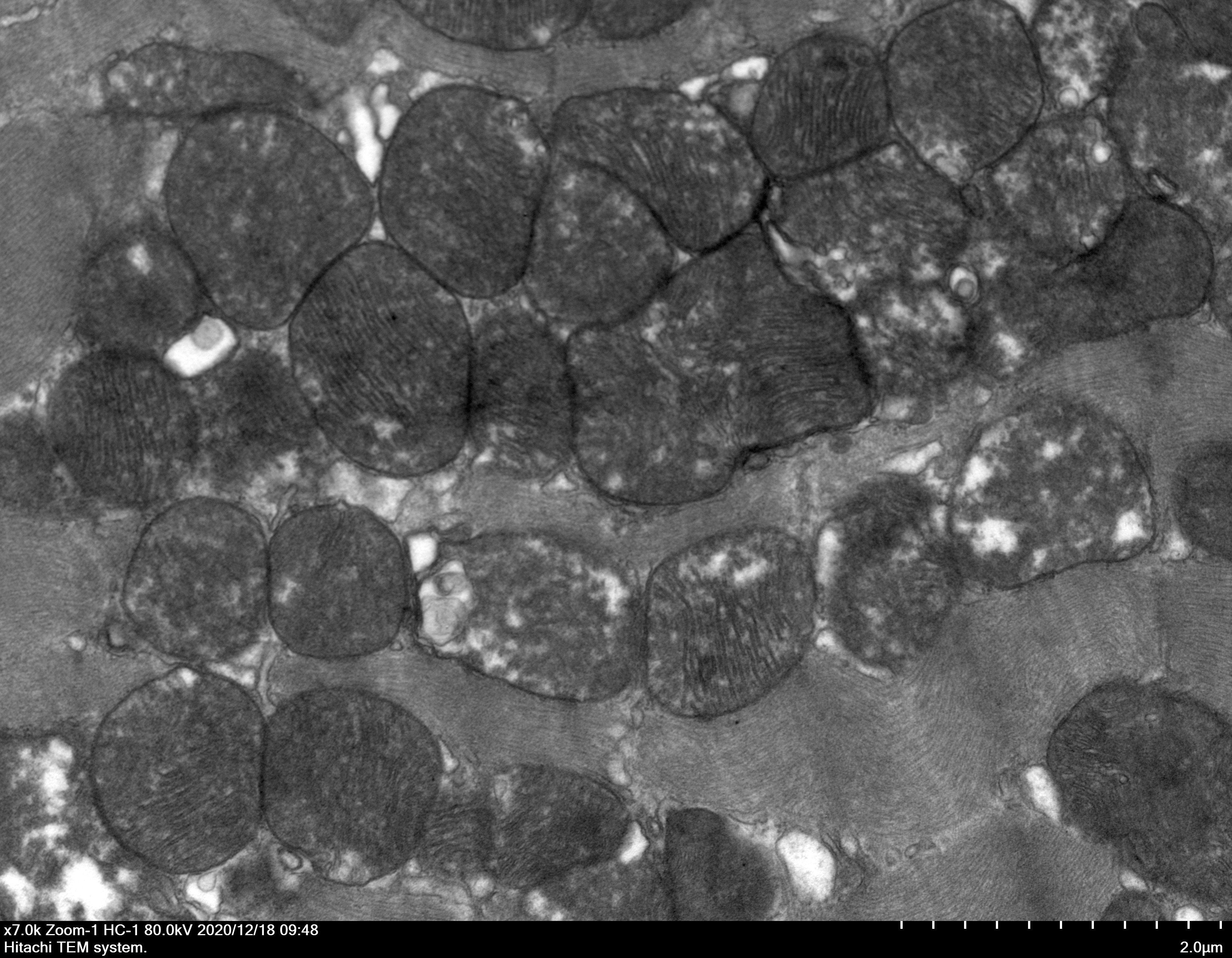

Supplement: Supplementary file 4 [file DataSheet8.ZIP › IR+P.tif]

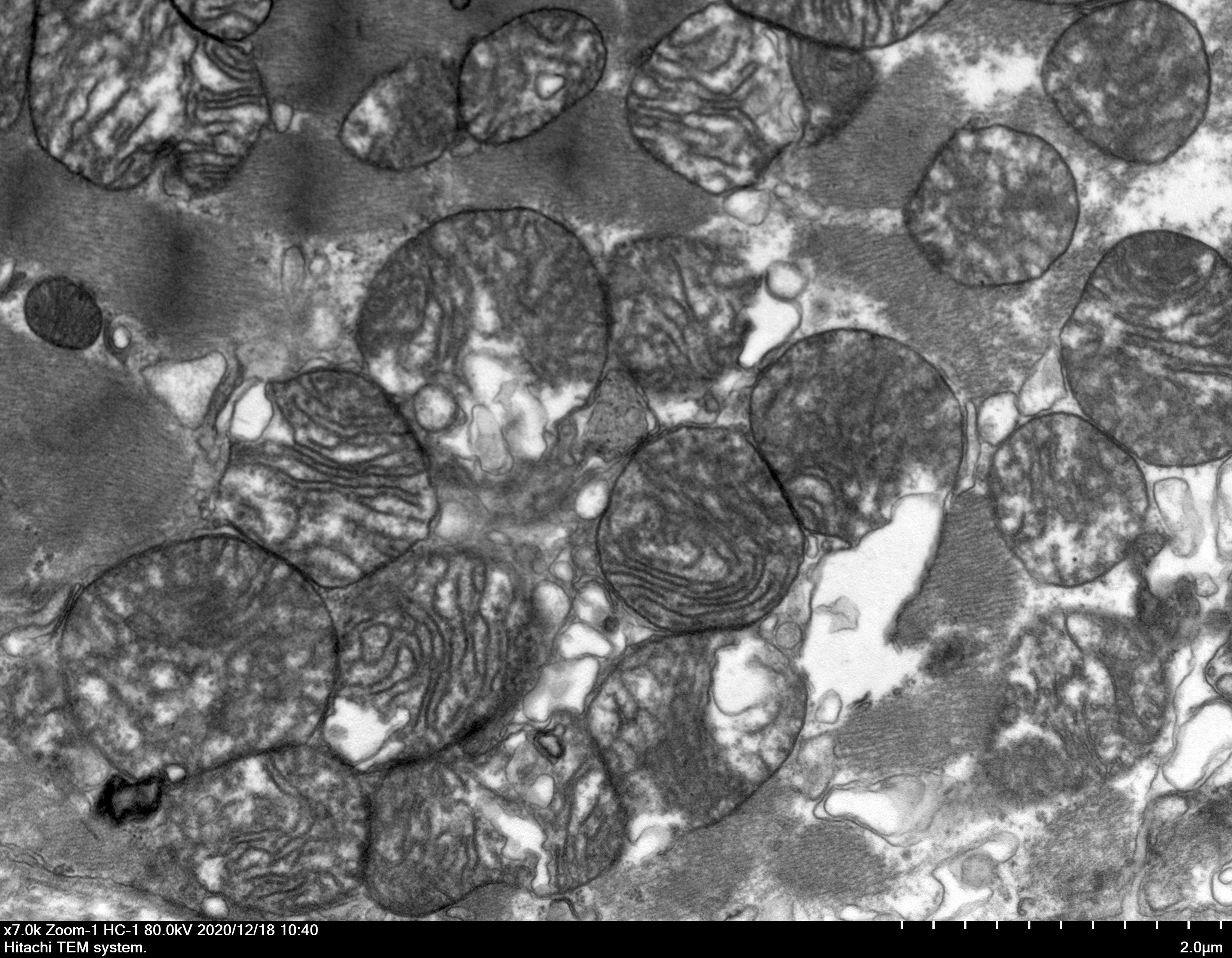

Supplement: Supplementary file 5 [file DataSheet9.ZIP › IR+P+MK.tif]

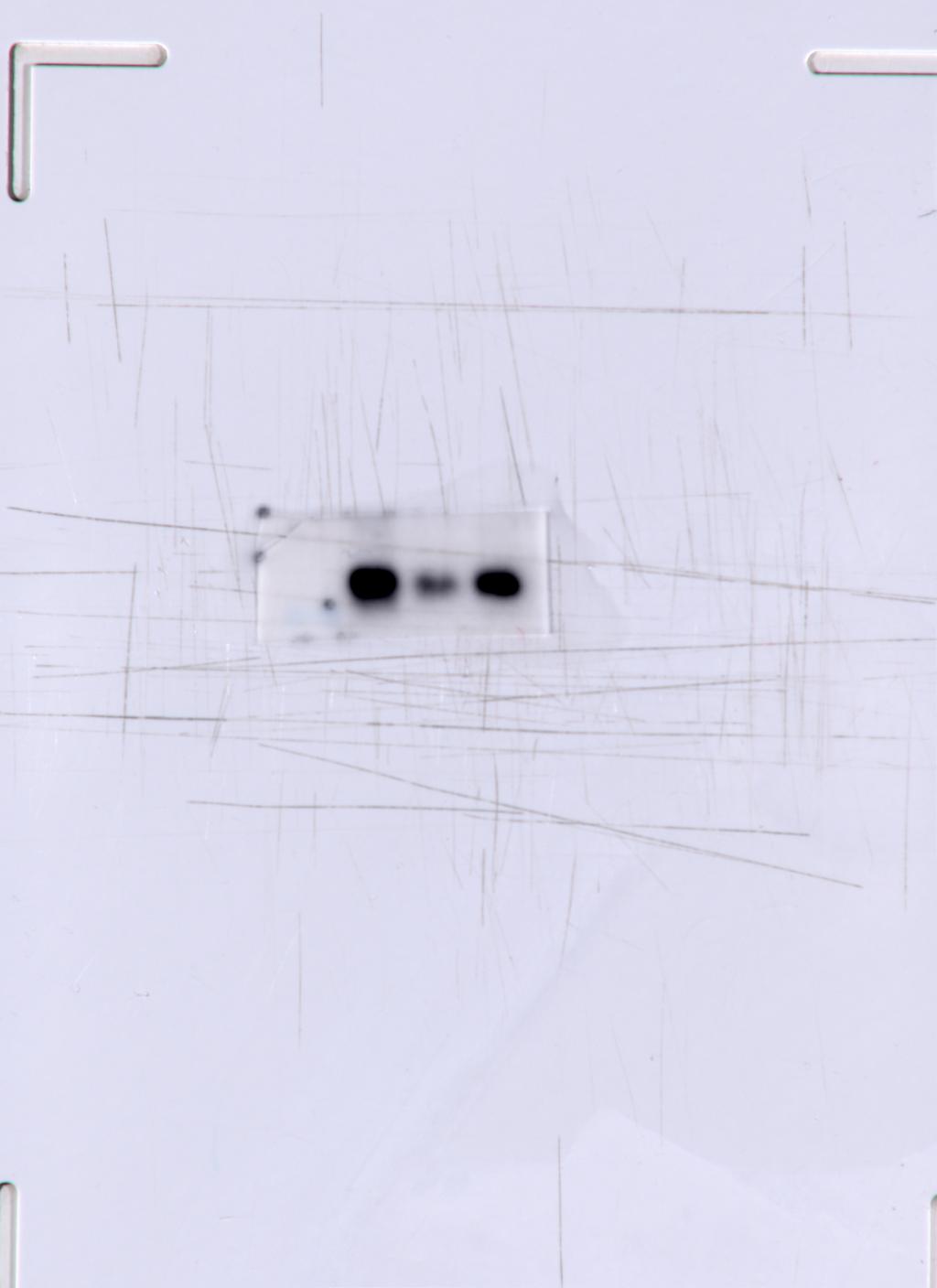

Supplement: Supplementary file 6 [file DataSheet4.ZIP › Fig4一/Fig4C-G/FTH1.jpg]

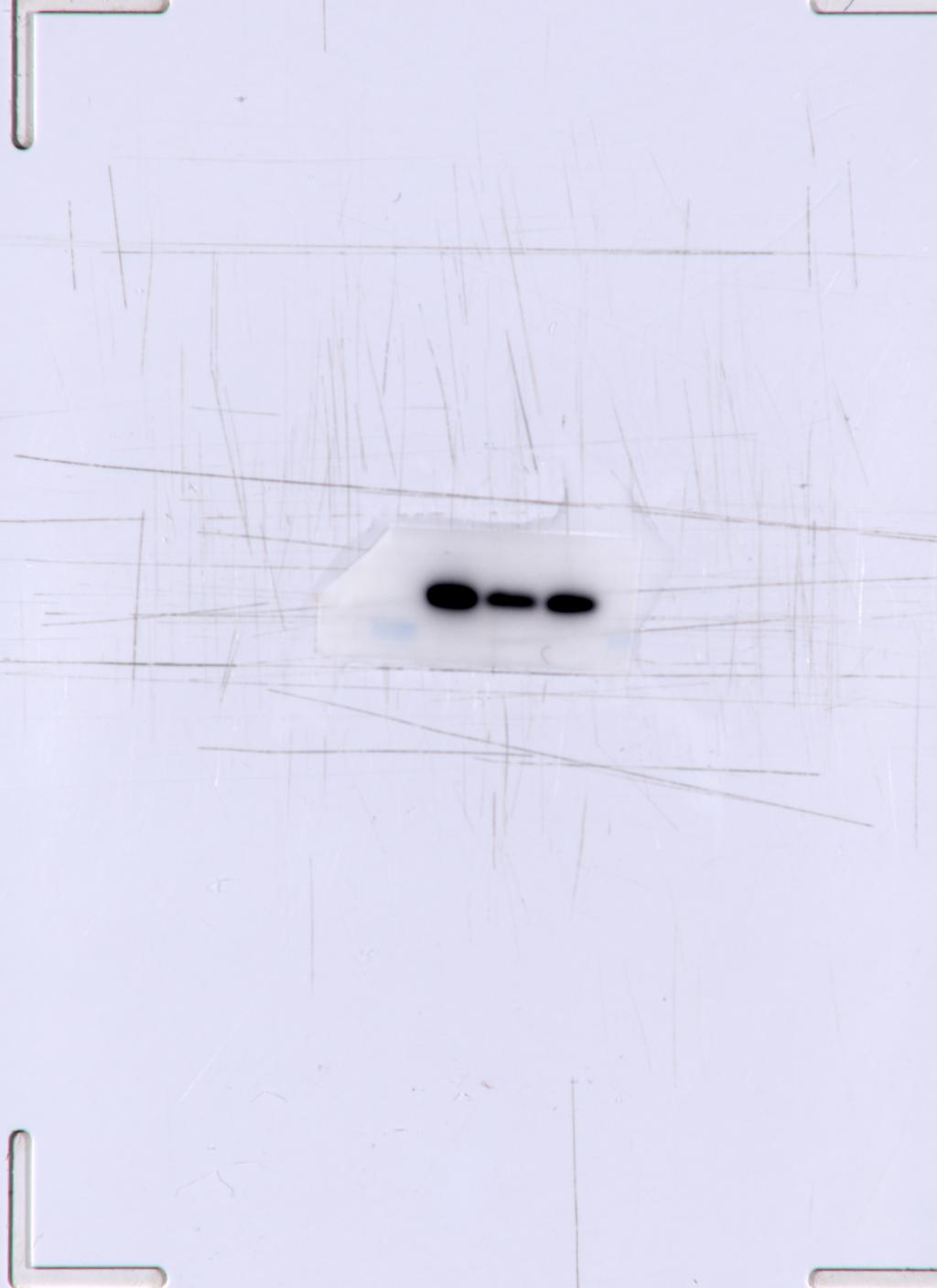

Supplement: Supplementary file 6 [file DataSheet4.ZIP › Fig4一/Fig4C-G/GPX4.jpg]

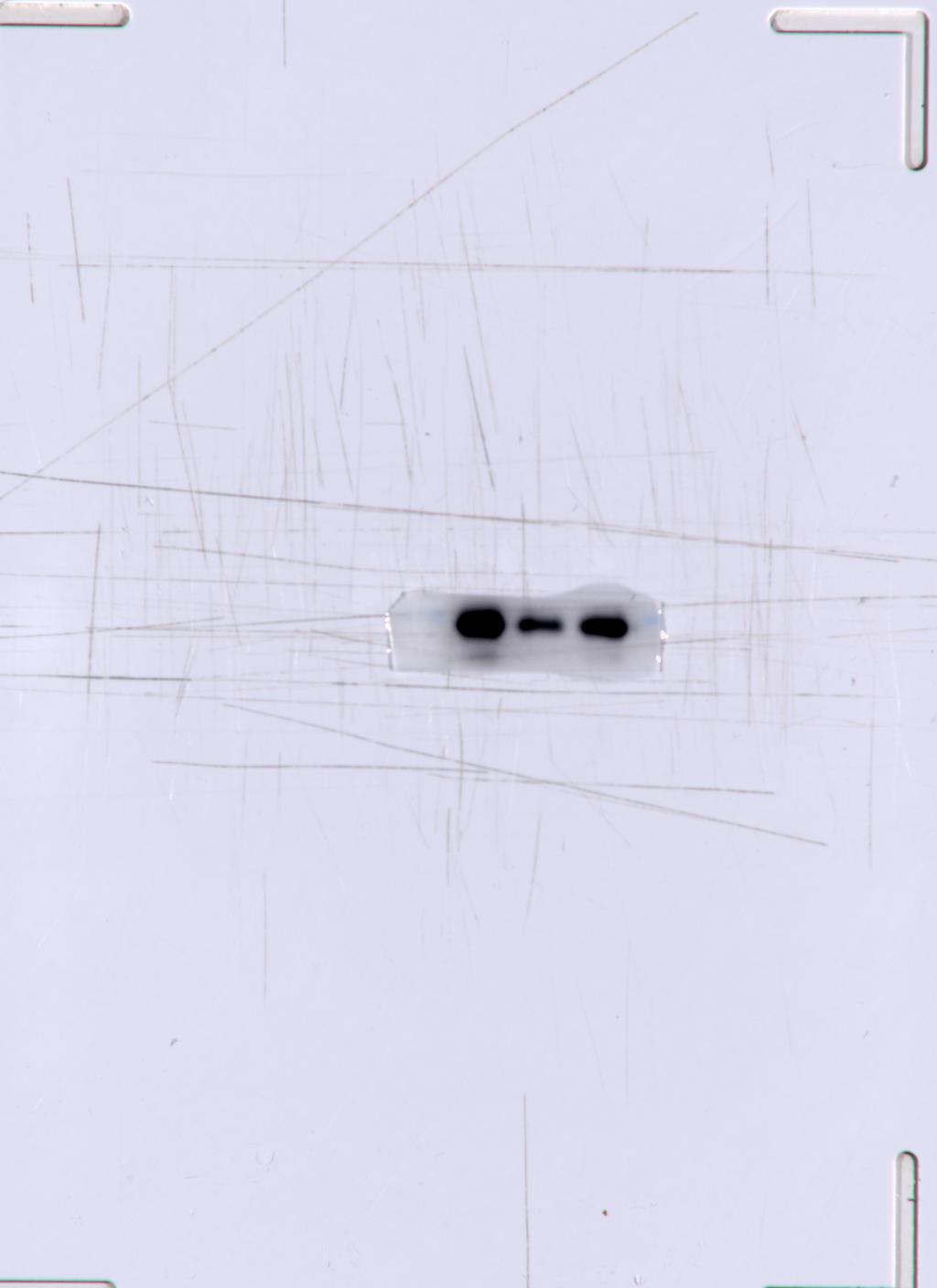

Supplement: Supplementary file 6 [file DataSheet4.ZIP › Fig4一/Fig4C-G/SOD-2.jpg]

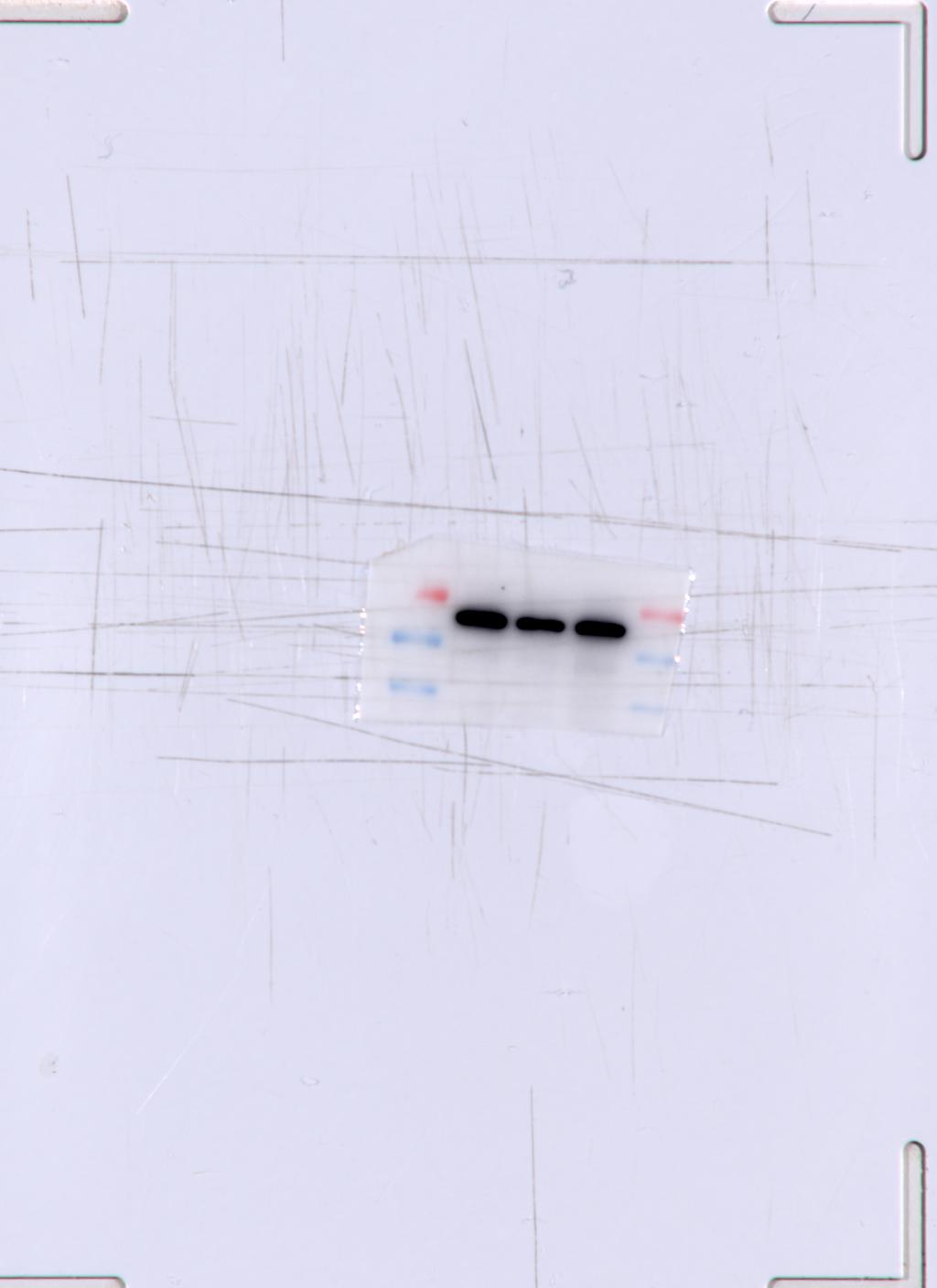

Supplement: Supplementary file 6 [file DataSheet4.ZIP › Fig4一/Fig4C-G/XCT.jpg]

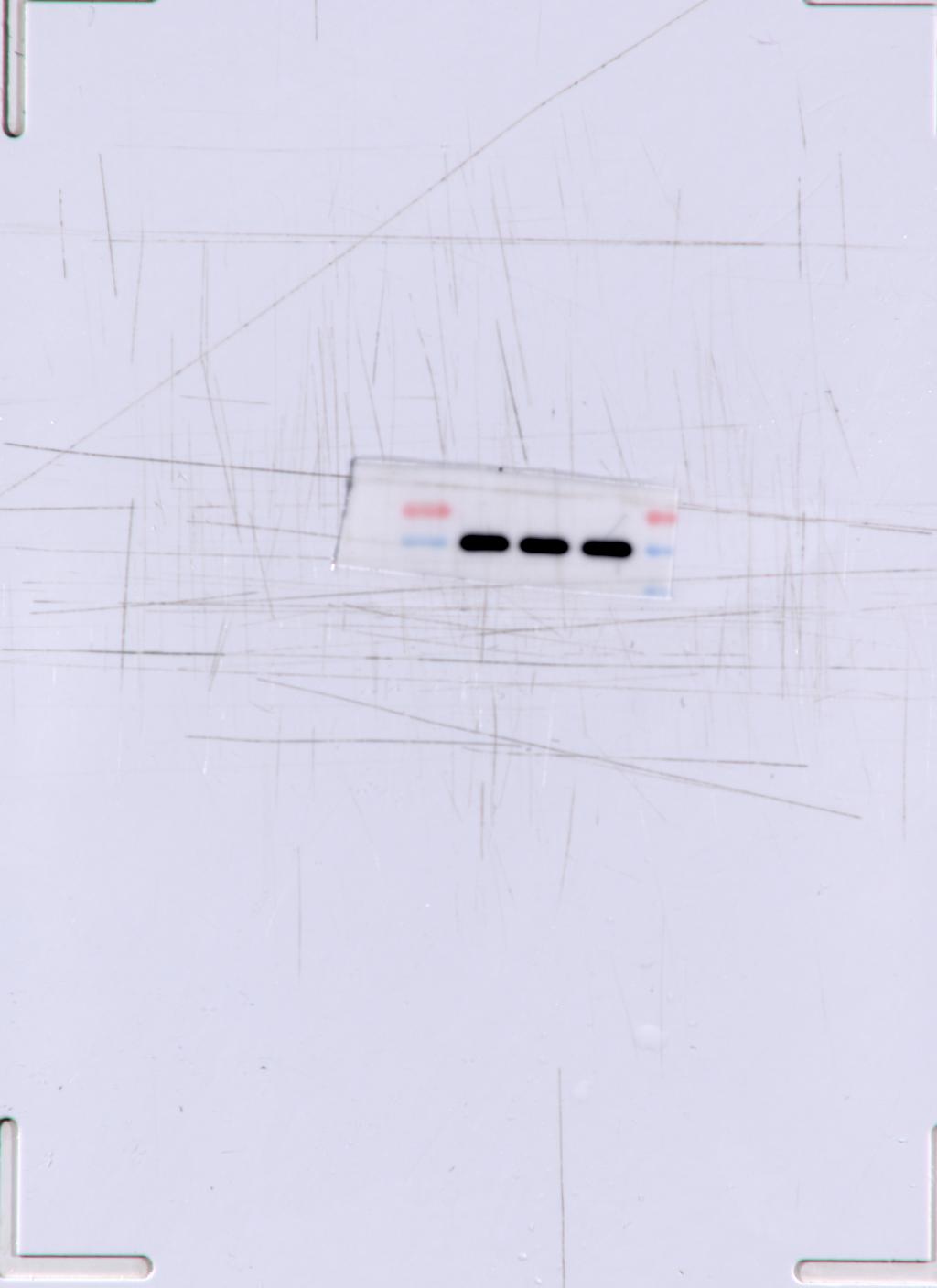

Supplement: Supplementary file 6 [file DataSheet4.ZIP › Fig4一/Fig4C-G/α-tubulin.jpg]

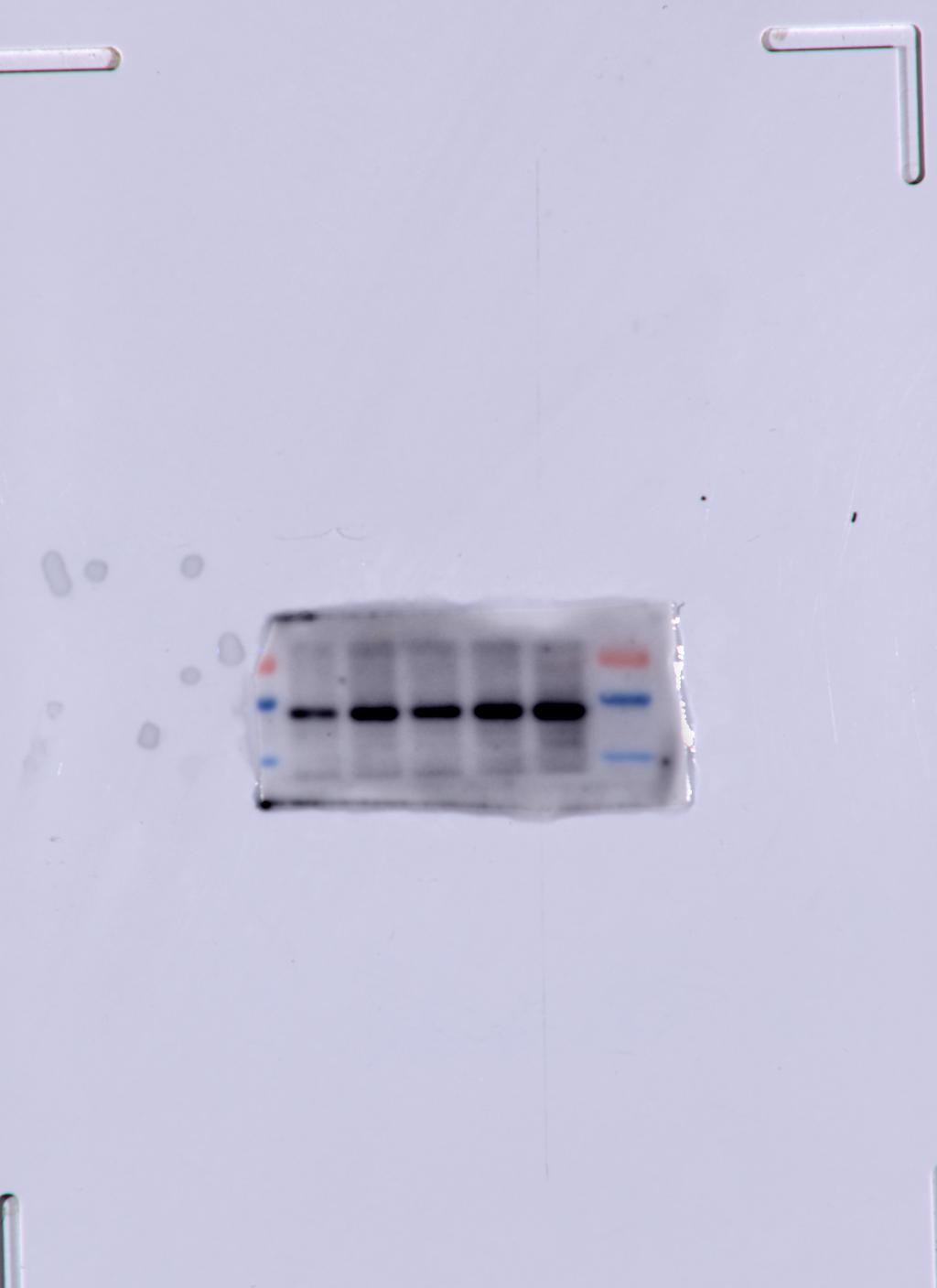

Supplement: Supplementary file 6 [file DataSheet4.ZIP › Fig4一/Fig4L-N/P-P53.jpg]

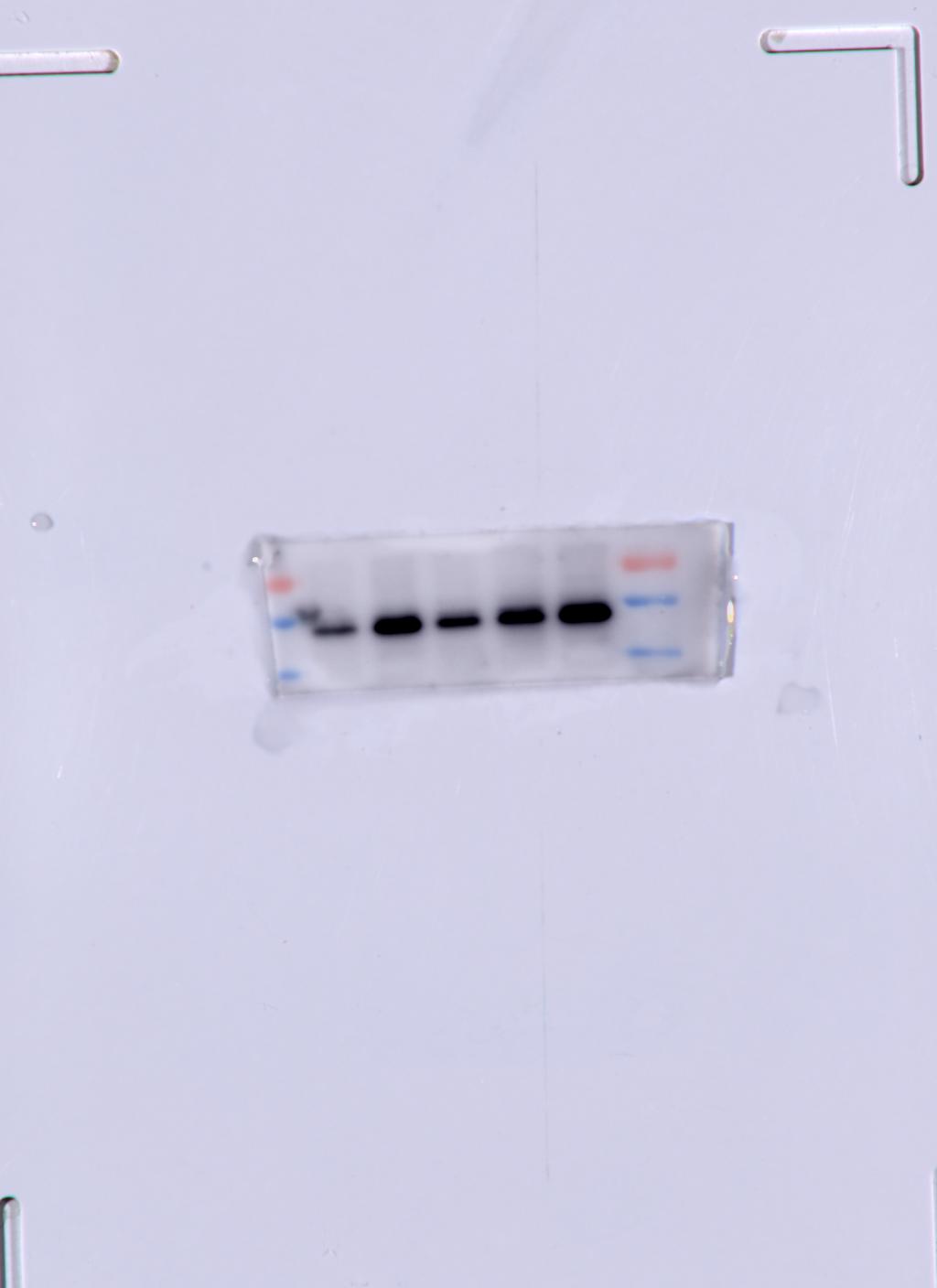

Supplement: Supplementary file 6 [file DataSheet4.ZIP › Fig4一/Fig4L-N/p53.jpg]

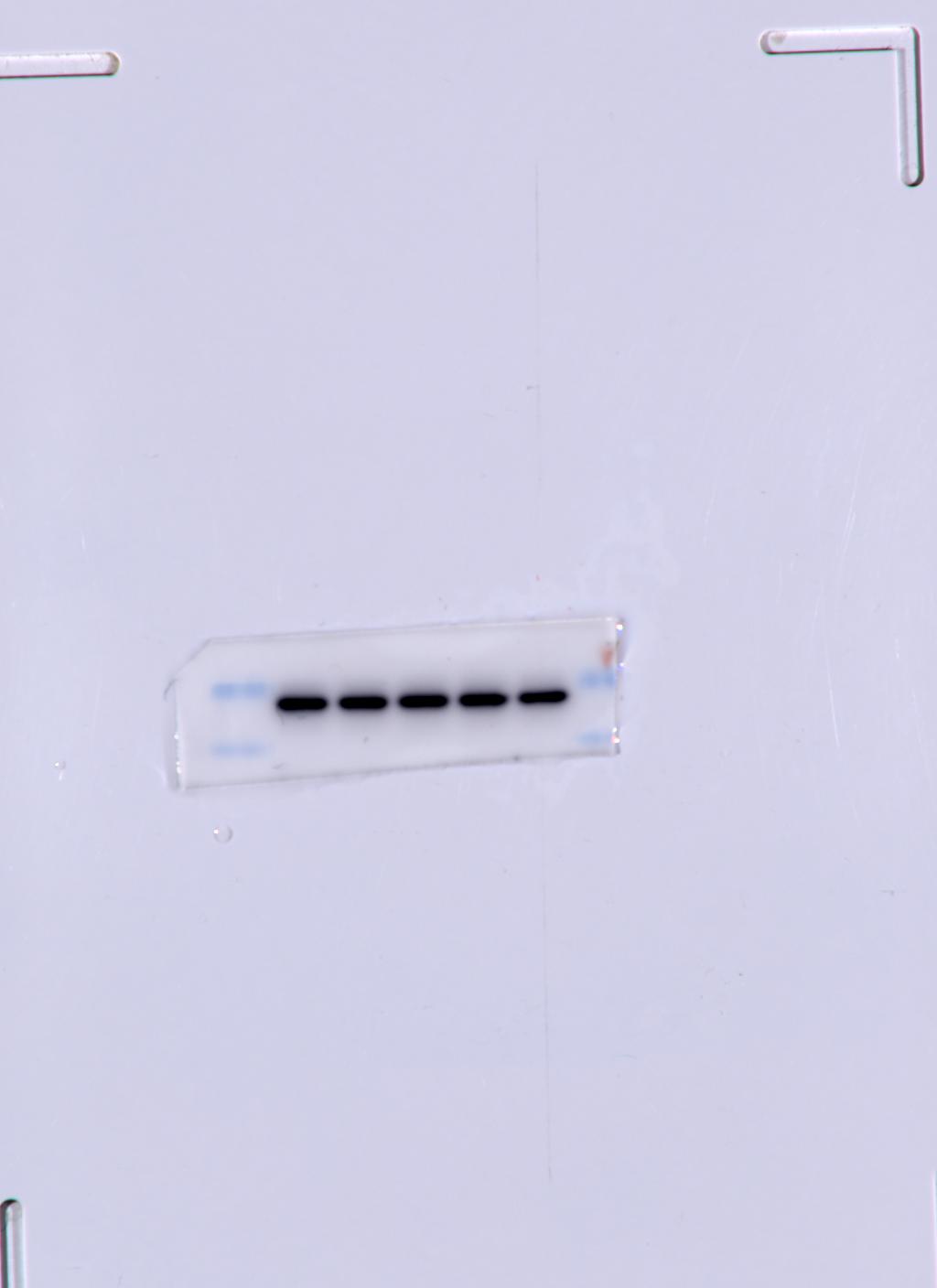

Supplement: Supplementary file 6 [file DataSheet4.ZIP › Fig4一/Fig4L-N/α-tubulin.jpg]

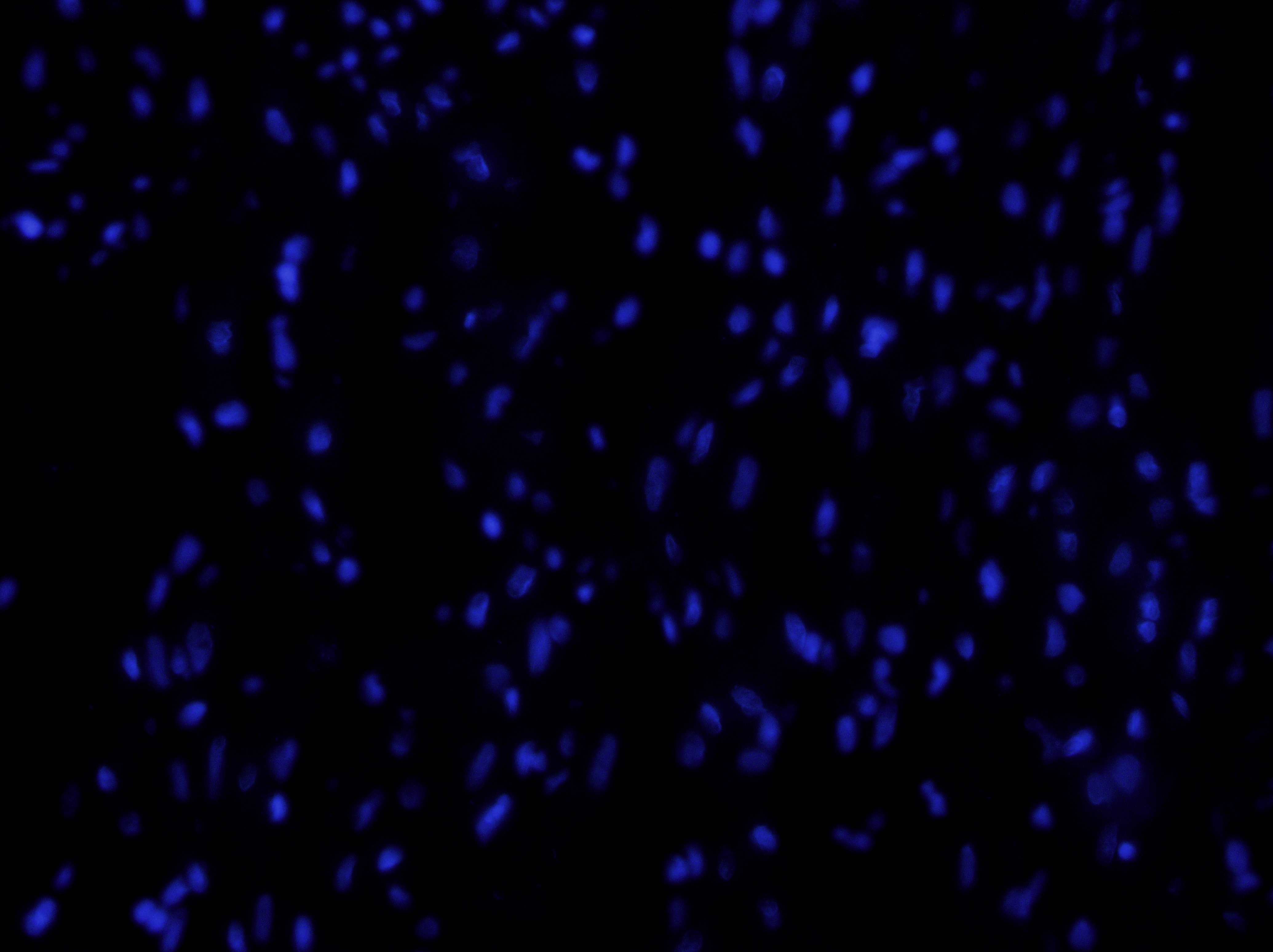

Supplement: Supplementary file 7 [file DataSheet13.ZIP › Fig5二1/Control dapi.tif]

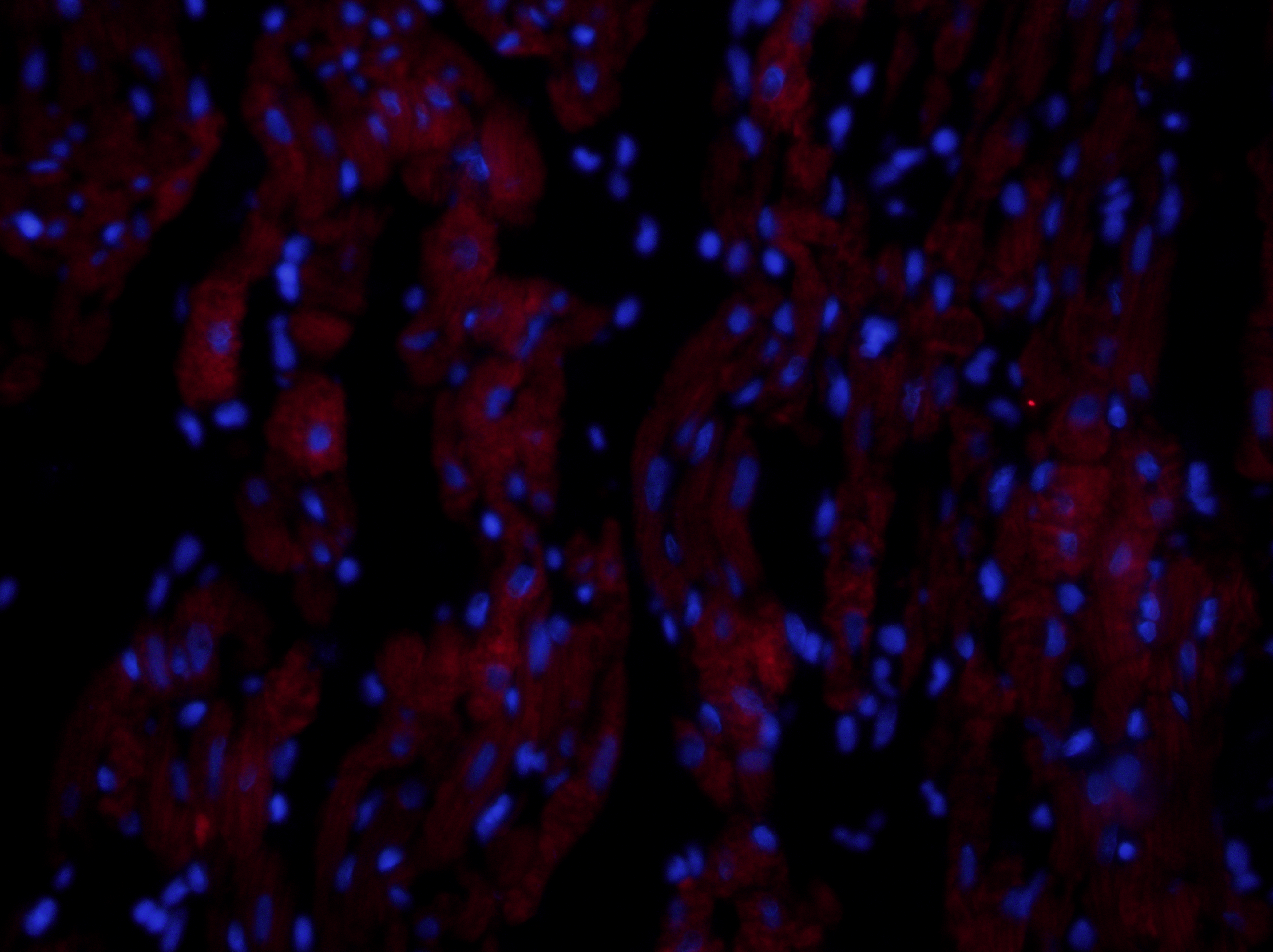

Supplement: Supplementary file 7 [file DataSheet13.ZIP › Fig5二1/Control merge.tif]

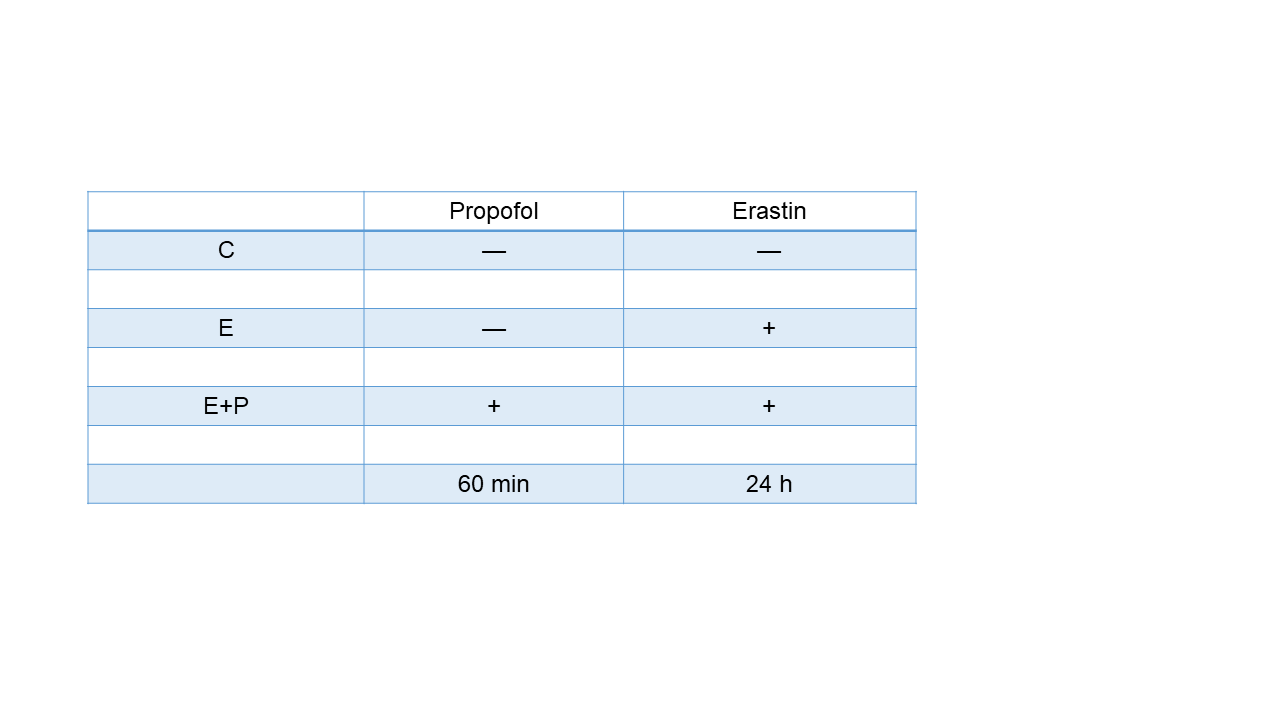

Supplement: Supplementary file 8 [file DataSheet1.ZIP › Fig1/H9C2.tif]

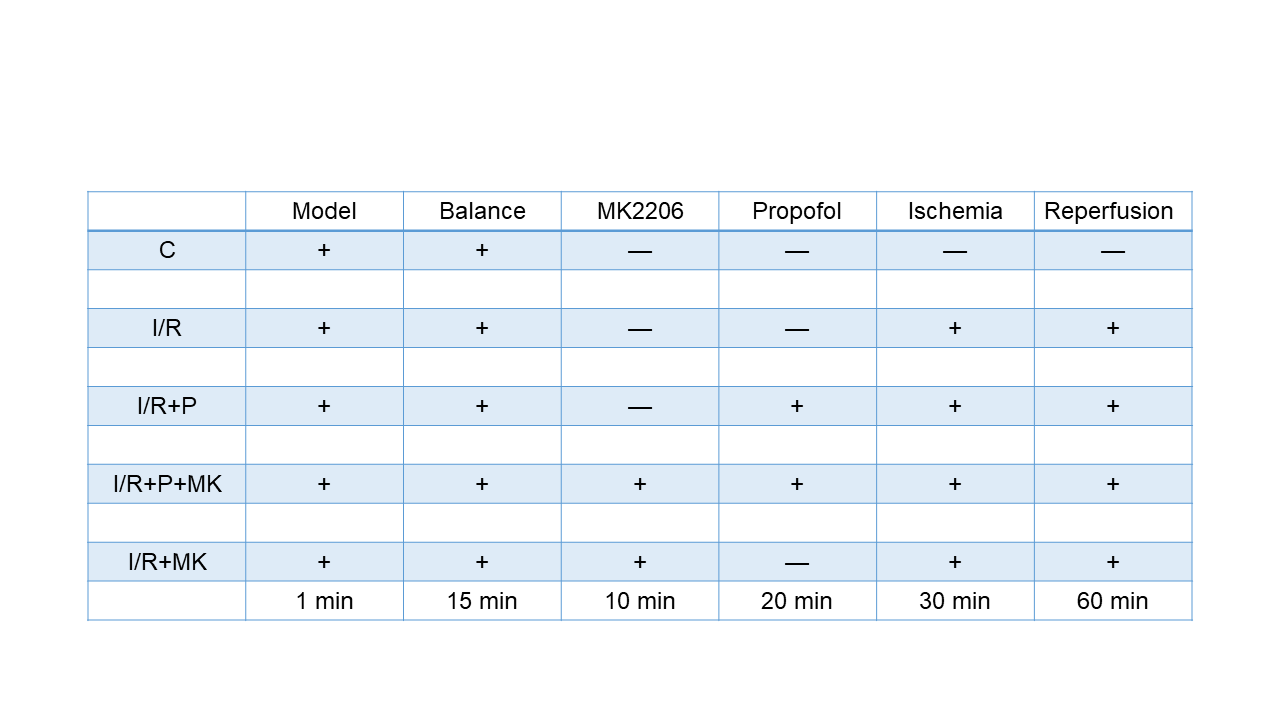

Supplement: Supplementary file 8 [file DataSheet1.ZIP › Fig1/langendorff.tif]

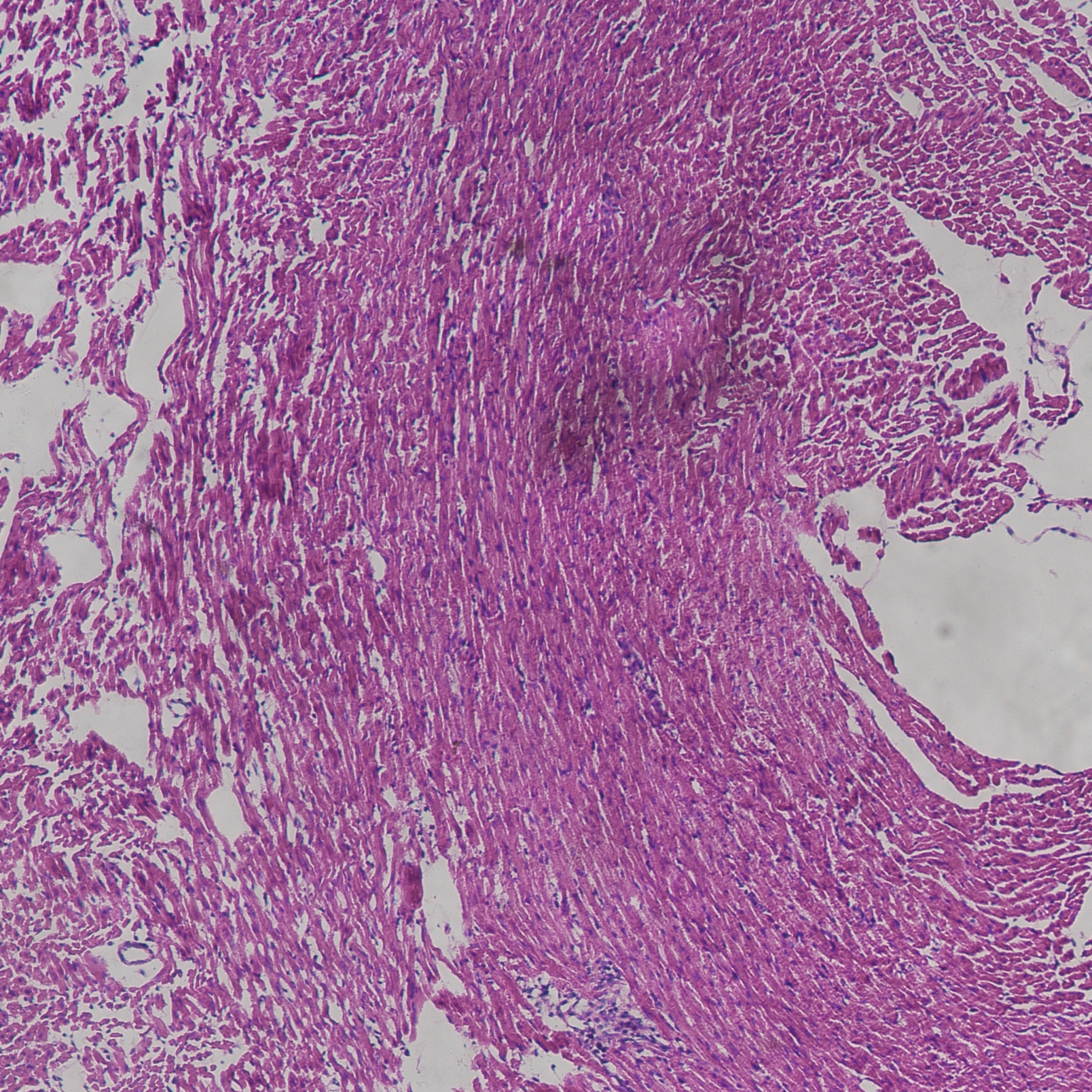

Supplement: Supplementary file 9 [file DataSheet10.ZIP › Fig4三1/Control.tif]

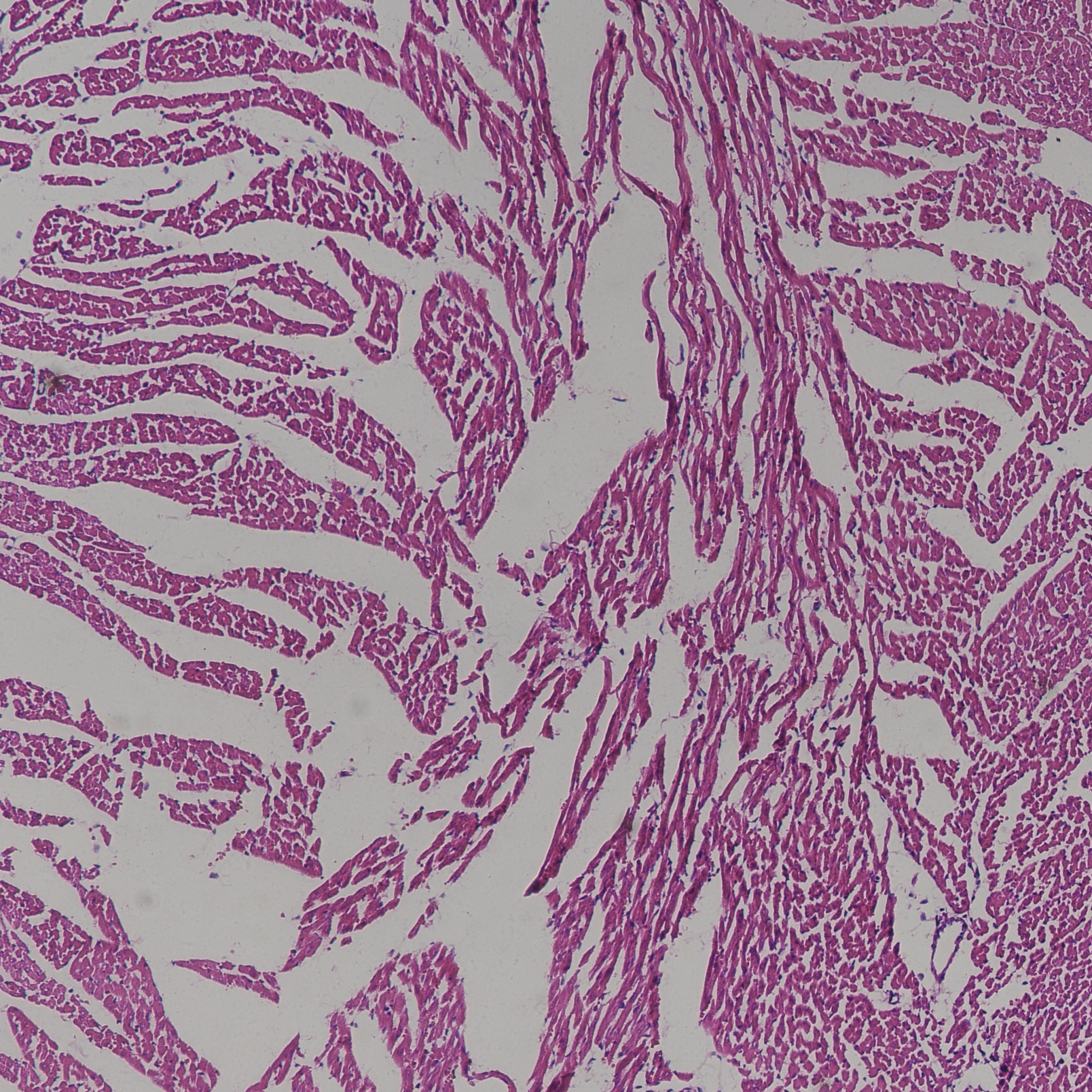

Supplement: Supplementary file 9 [file DataSheet10.ZIP › Fig4三1/IR+MK.tif]

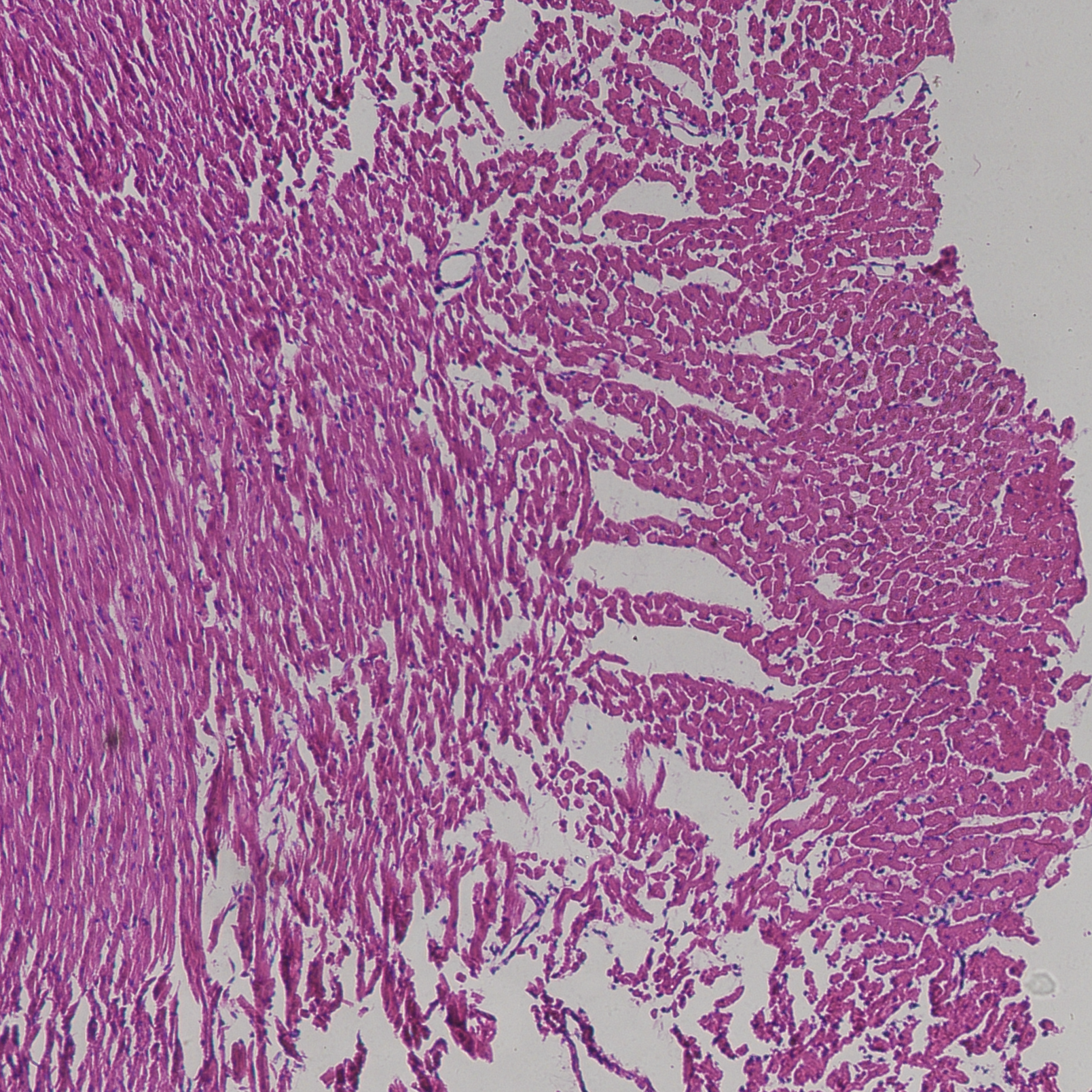

Supplement: Supplementary file 9 [file DataSheet10.ZIP › Fig4三1/IR.tif]

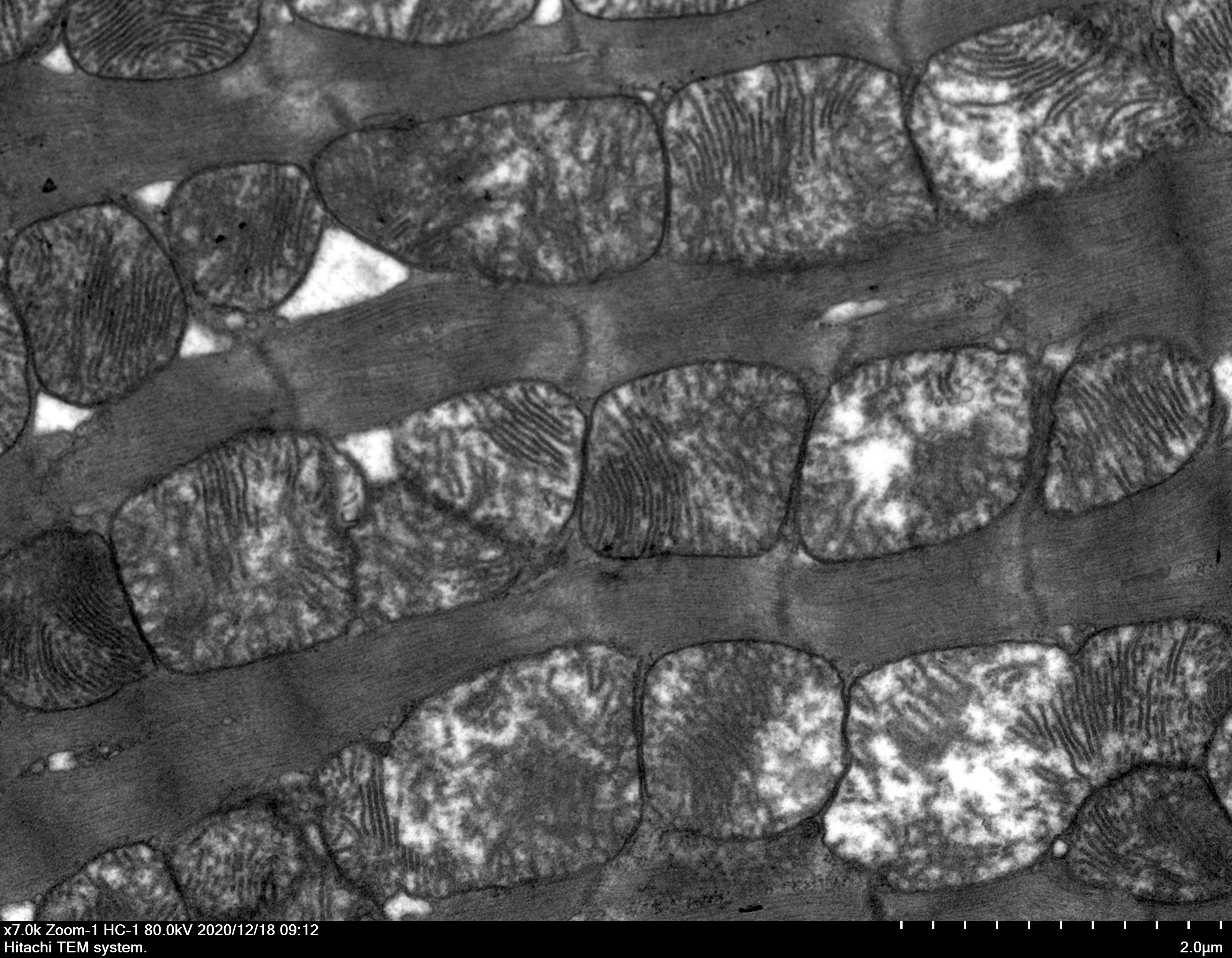

Supplement: Supplementary file 10 [file DataSheet6.ZIP › IR.tif]

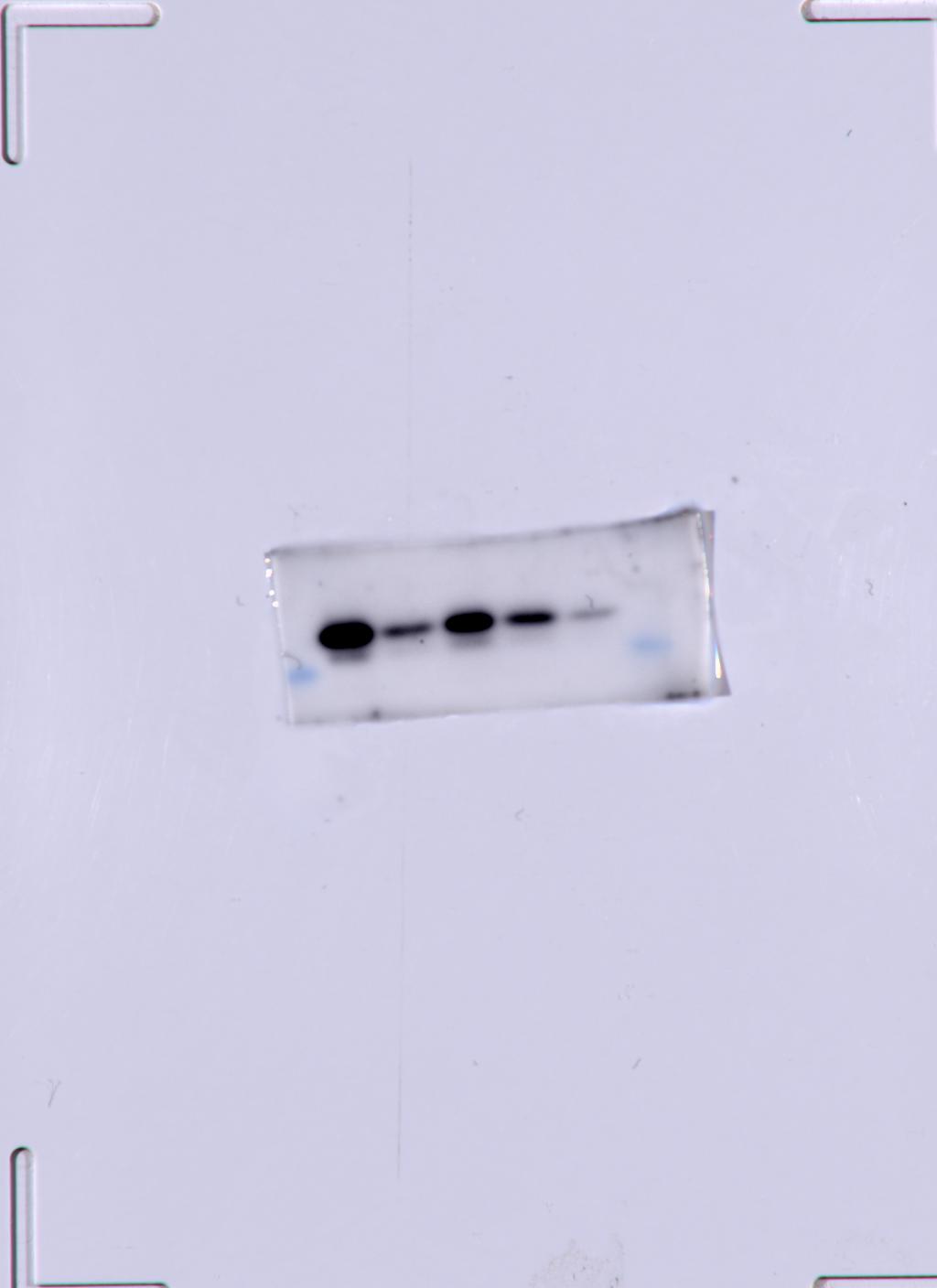

Supplement: Supplementary file 11 [file DataSheet12.ZIP › Fig5一/Fig5A-E/FTH1.jpg]

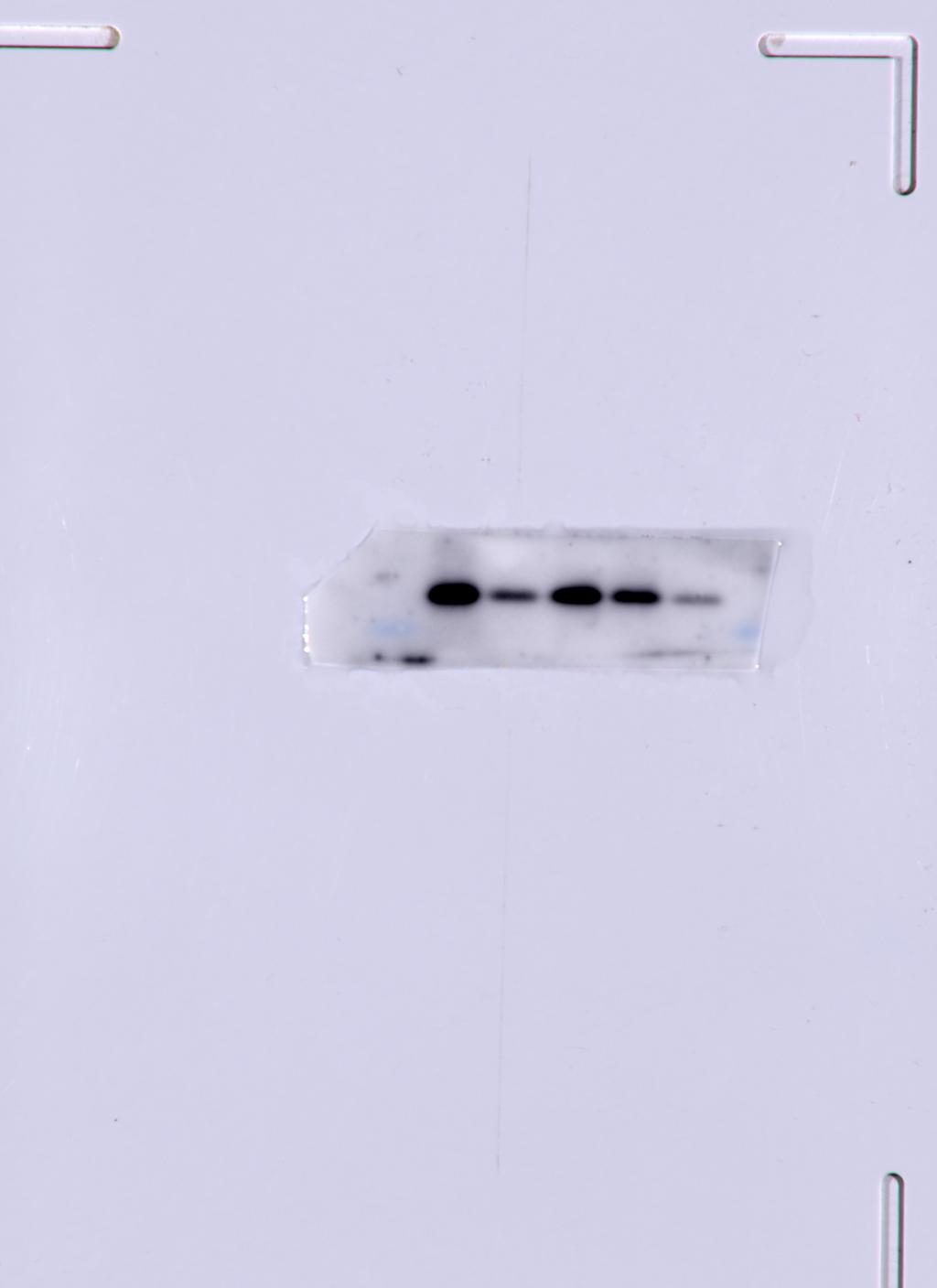

Supplement: Supplementary file 11 [file DataSheet12.ZIP › Fig5一/Fig5A-E/GPX4.jpg]

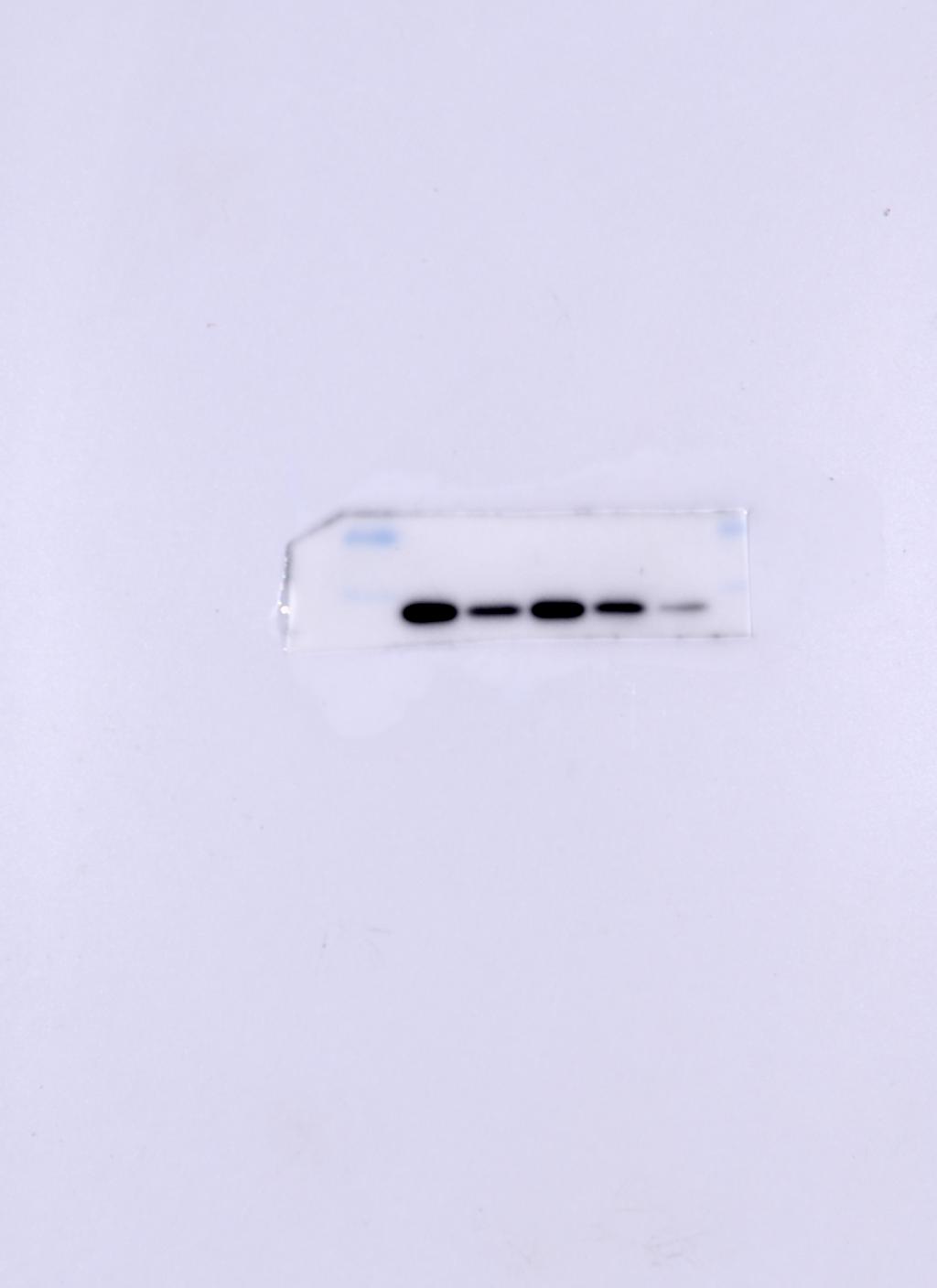

Supplement: Supplementary file 11 [file DataSheet12.ZIP › Fig5一/Fig5A-E/SOD-2.jpg]

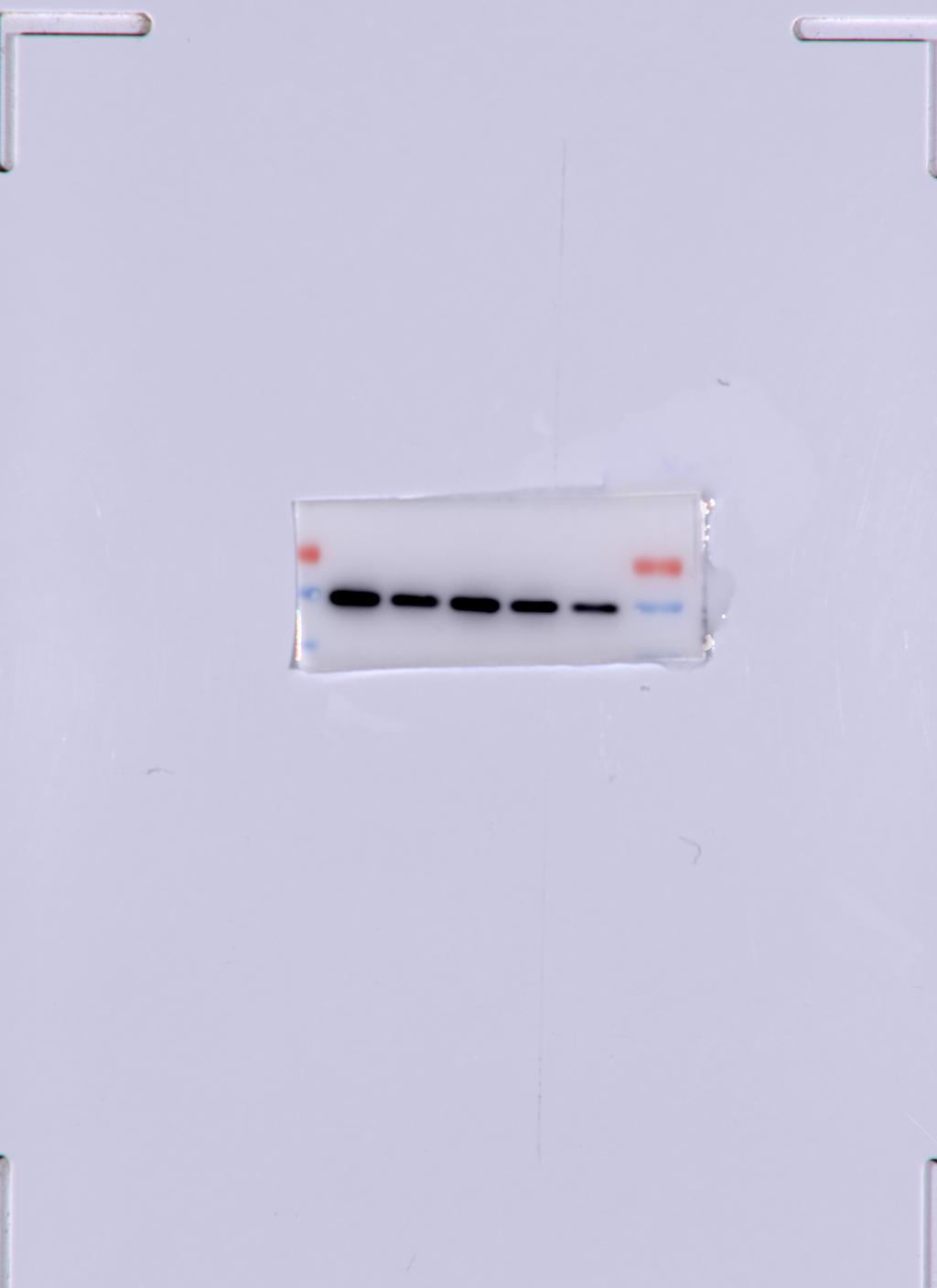

Supplement: Supplementary file 11 [file DataSheet12.ZIP › Fig5一/Fig5A-E/XCT.jpg]

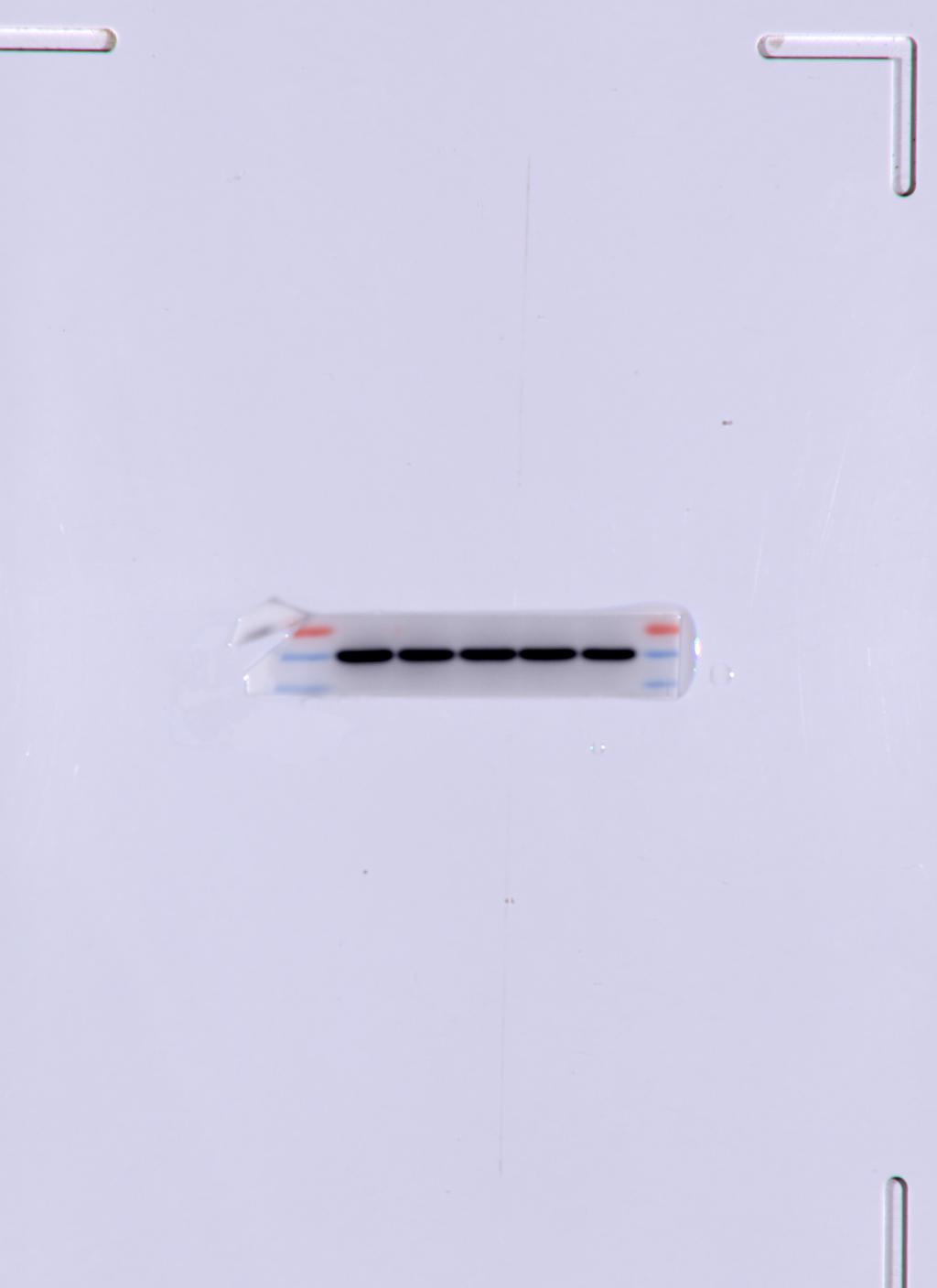

Supplement: Supplementary file 11 [file DataSheet12.ZIP › Fig5一/Fig5A-E/α-tubulin.jpg]

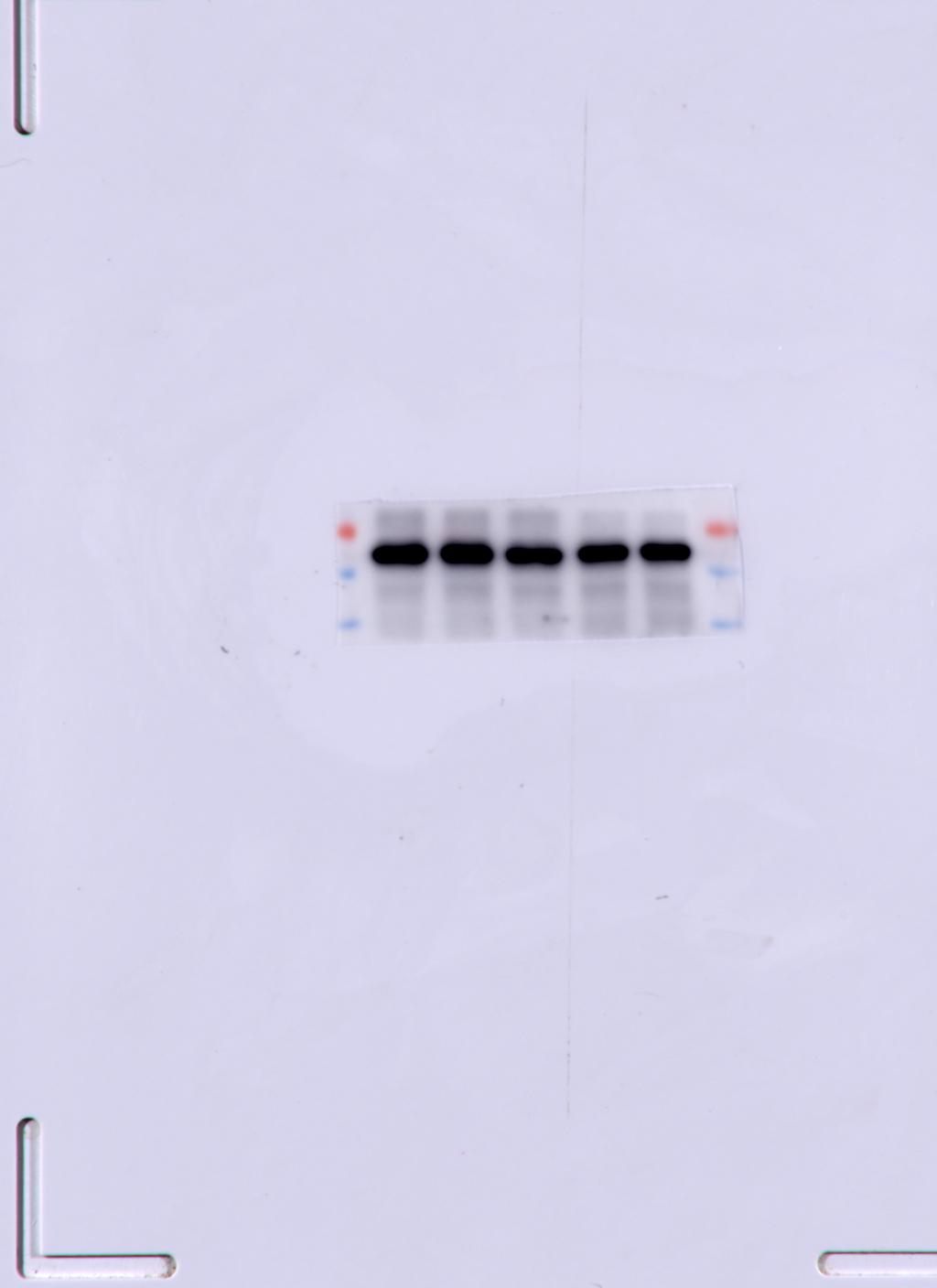

Supplement: Supplementary file 11 [file DataSheet12.ZIP › Fig5一/Fig5F,G/AKT.jpg]

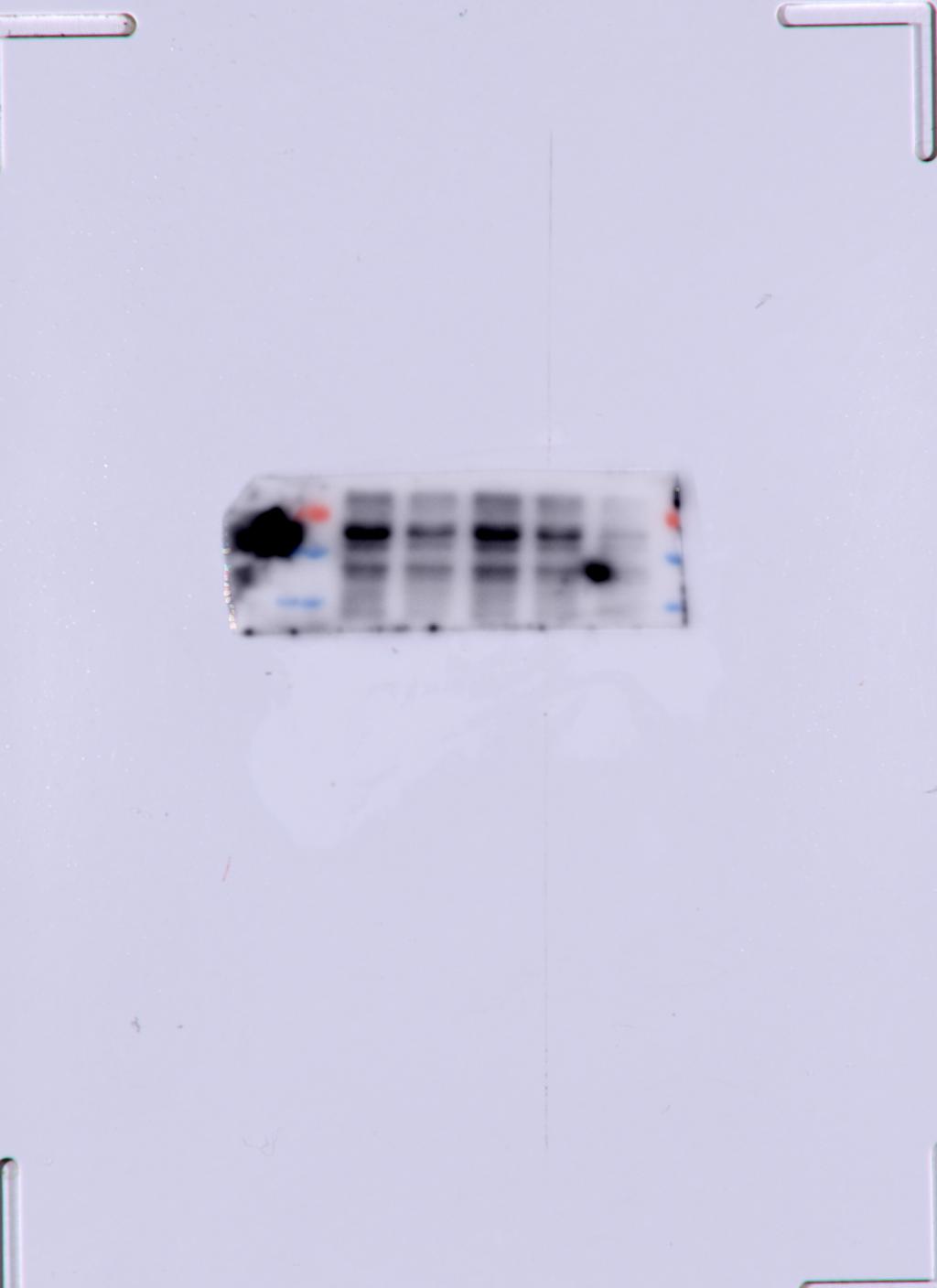

Supplement: Supplementary file 11 [file DataSheet12.ZIP › Fig5一/Fig5F,G/P-AKT.jpg]

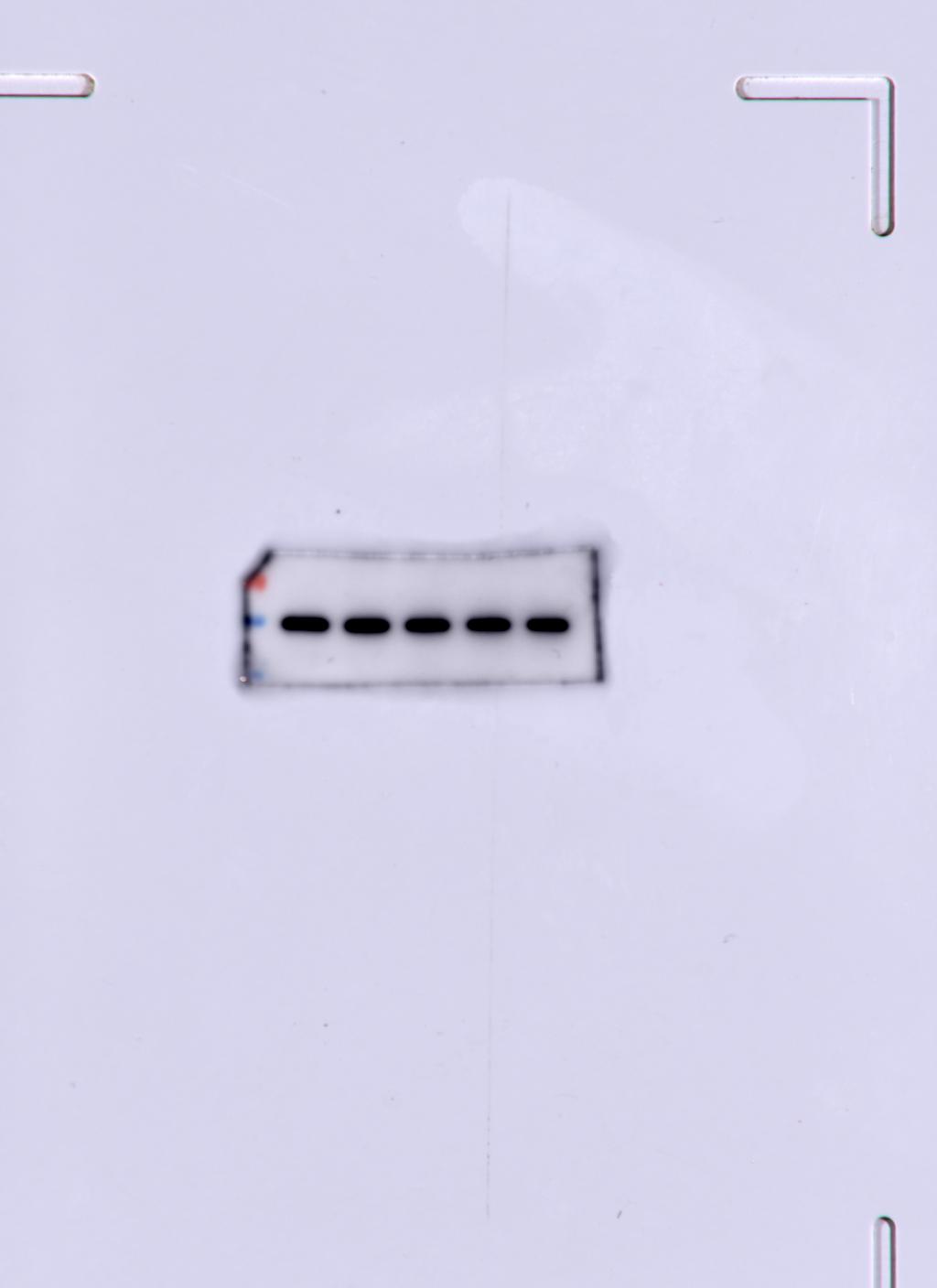

Supplement: Supplementary file 11 [file DataSheet12.ZIP › Fig5一/Fig5F,G/α-tubulin.jpg]

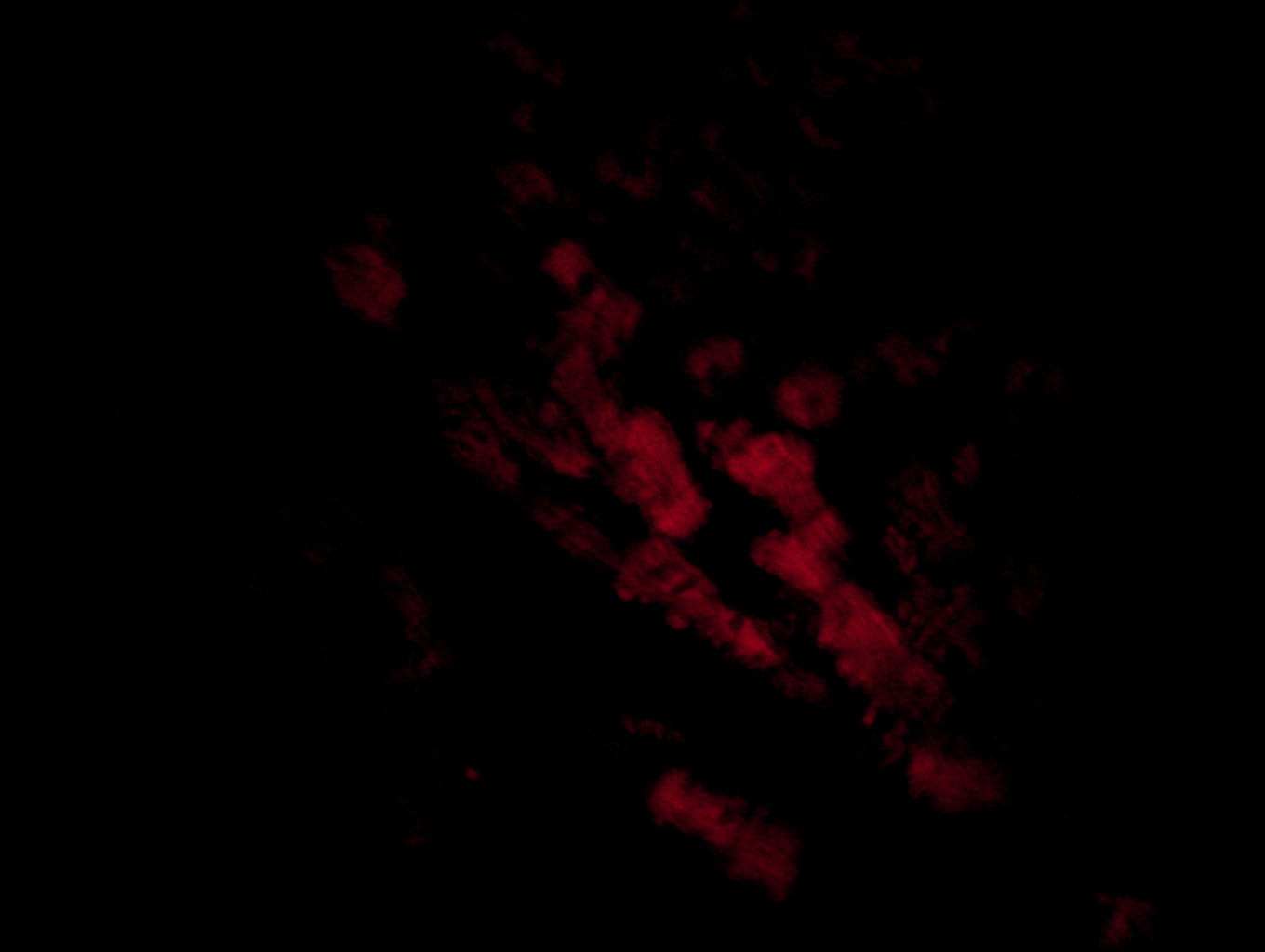

Supplement: Supplementary file 11 [file DataSheet12.ZIP › Fig5一/Fig5I,K/Control AKT.tif]

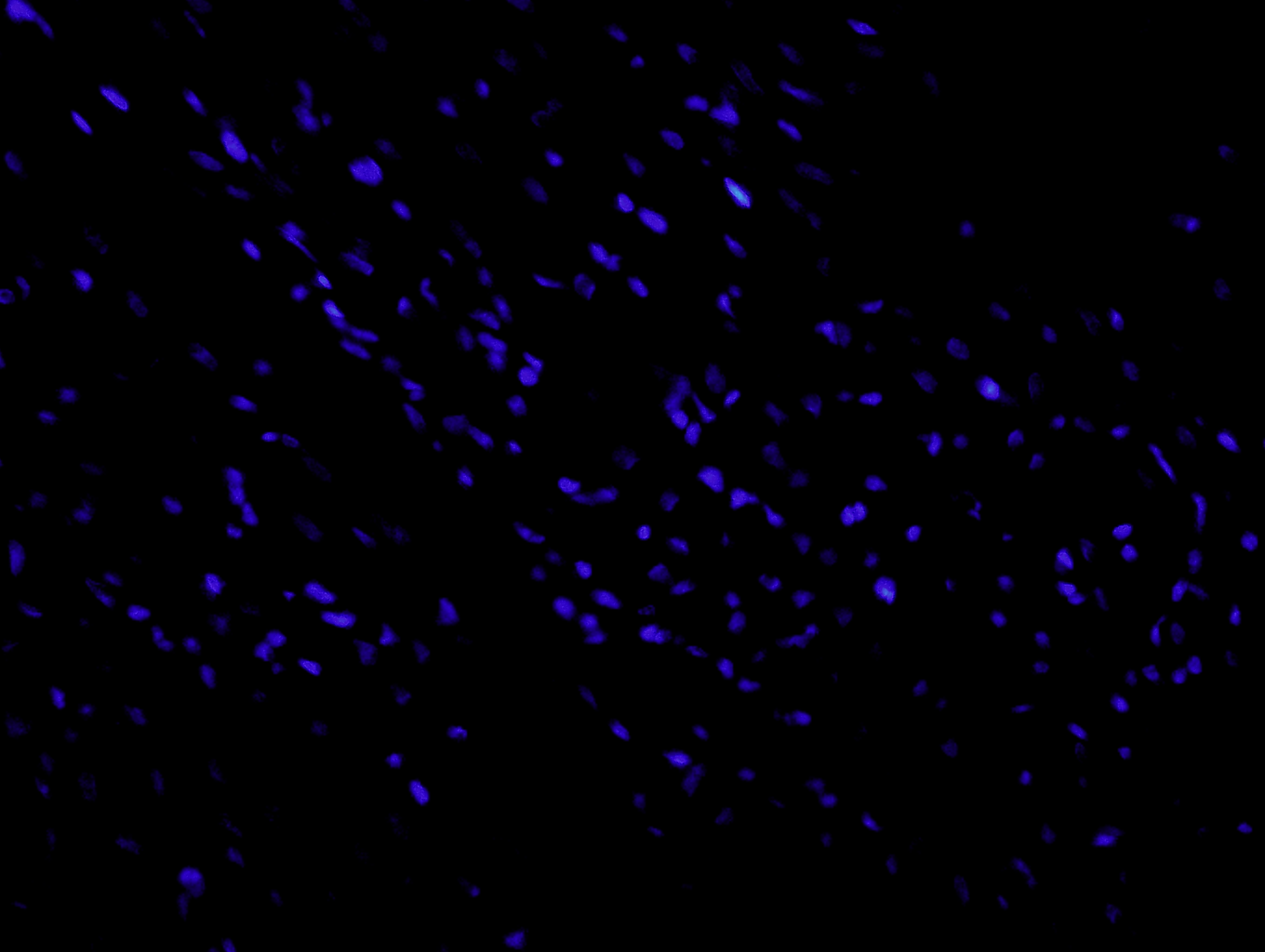

Supplement: Supplementary file 11 [file DataSheet12.ZIP › Fig5一/Fig5I,K/Control dapi.tif]

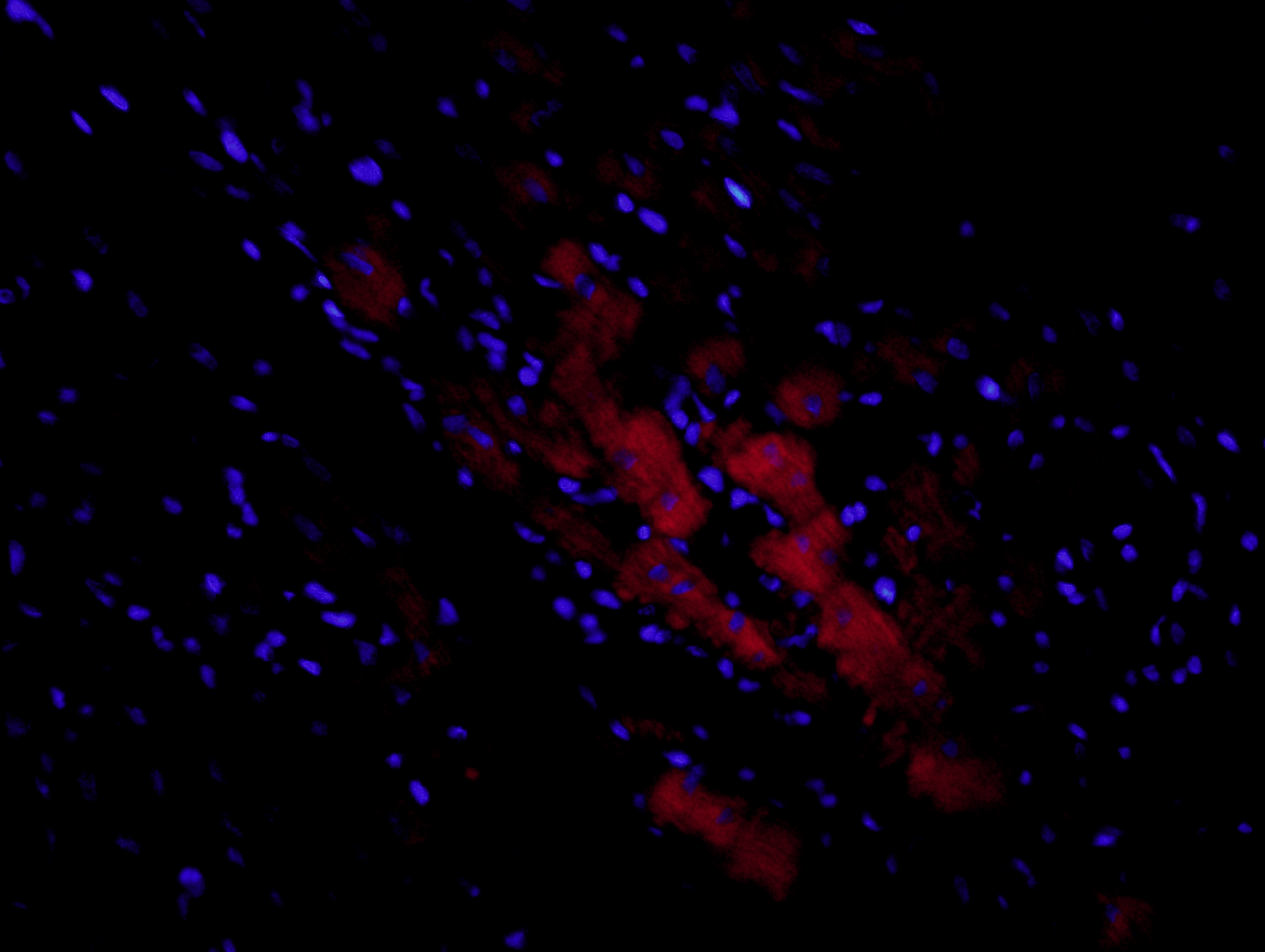

Supplement: Supplementary file 11 [file DataSheet12.ZIP › Fig5一/Fig5I,K/Control merge.tif]

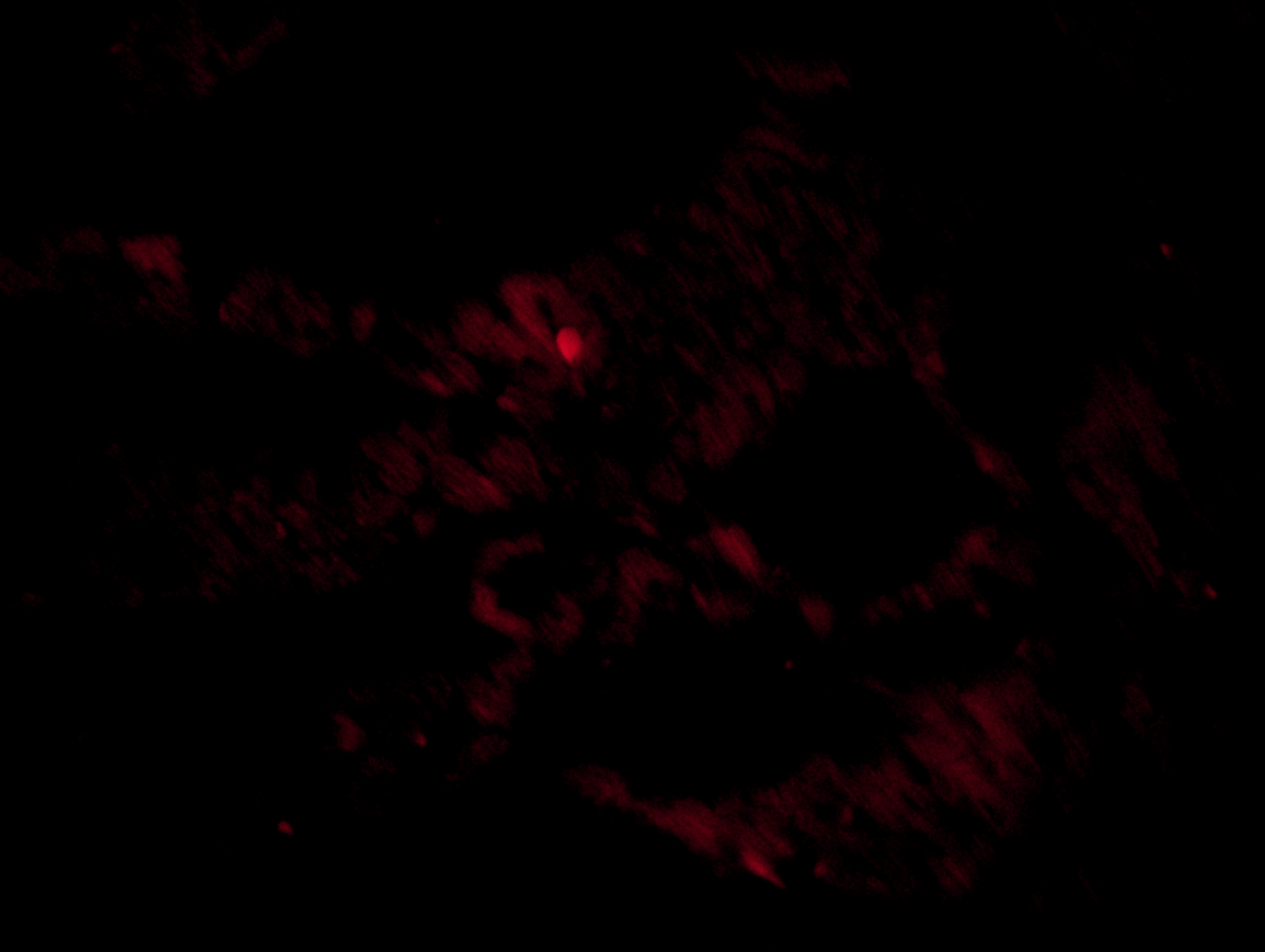

Supplement: Supplementary file 11 [file DataSheet12.ZIP › Fig5一/Fig5I,K/IR AKT.tif]

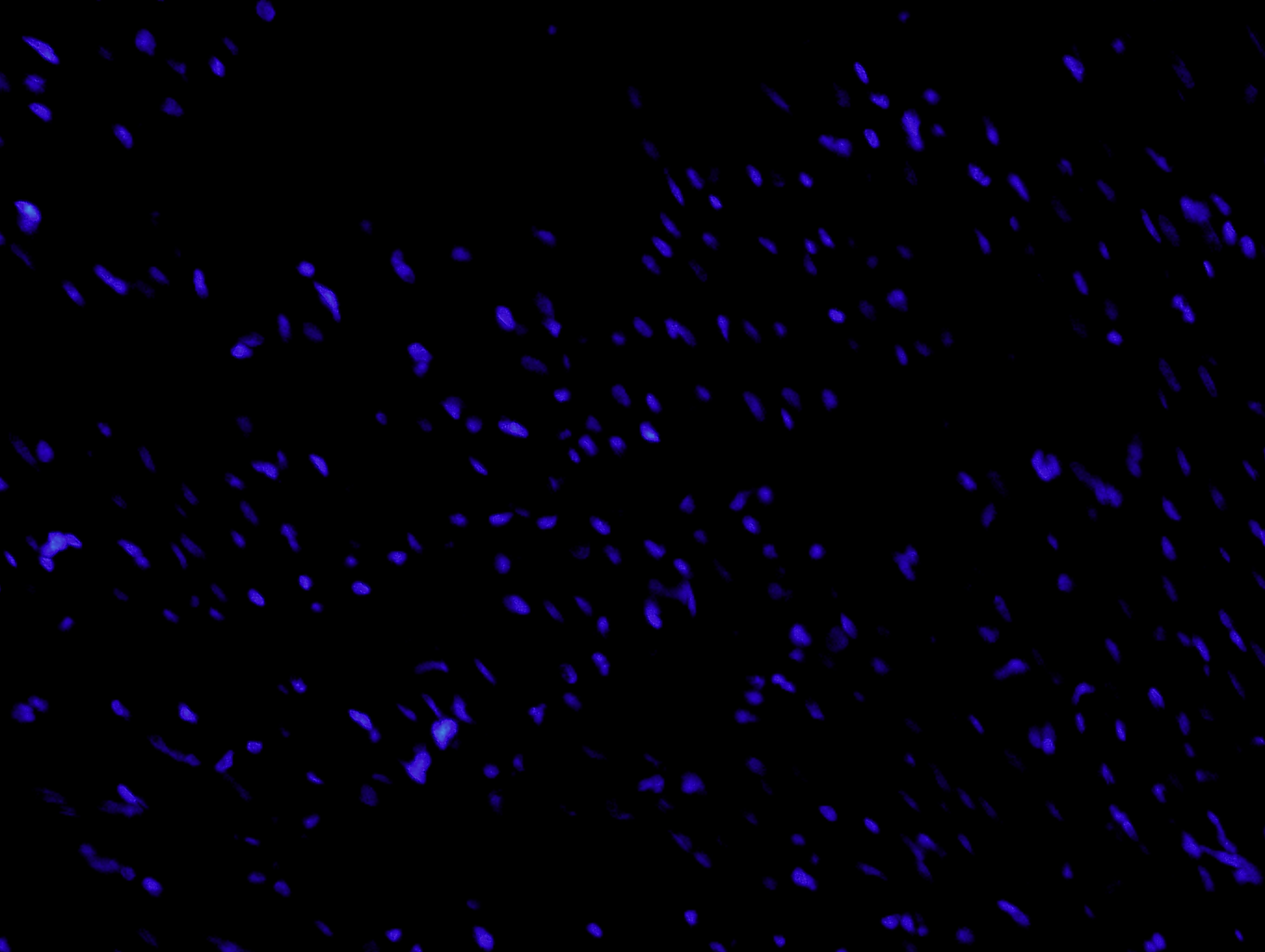

Supplement: Supplementary file 11 [file DataSheet12.ZIP › Fig5一/Fig5I,K/IR dapi.tif]

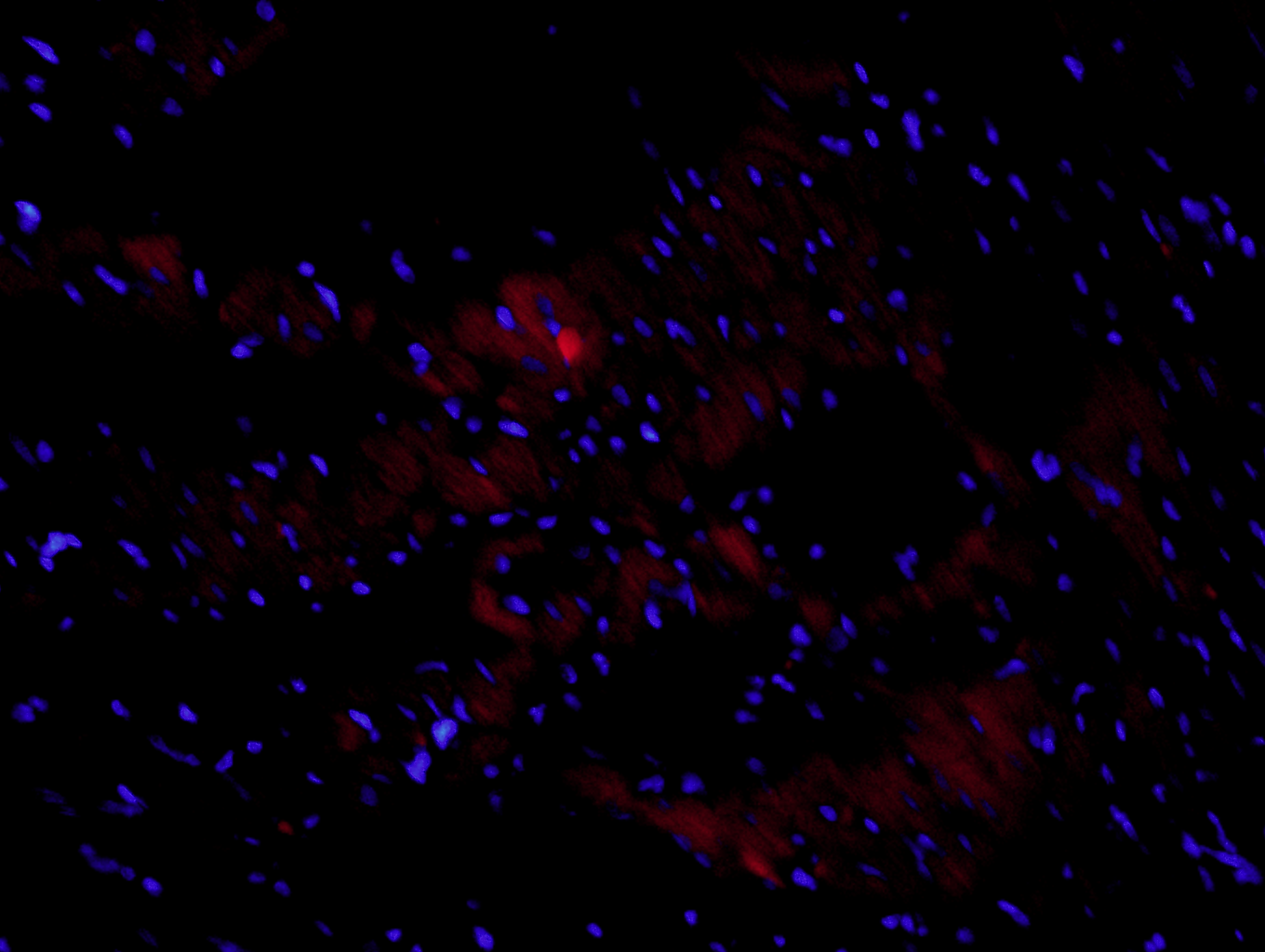

Supplement: Supplementary file 11 [file DataSheet12.ZIP › Fig5一/Fig5I,K/IR merge.tif]

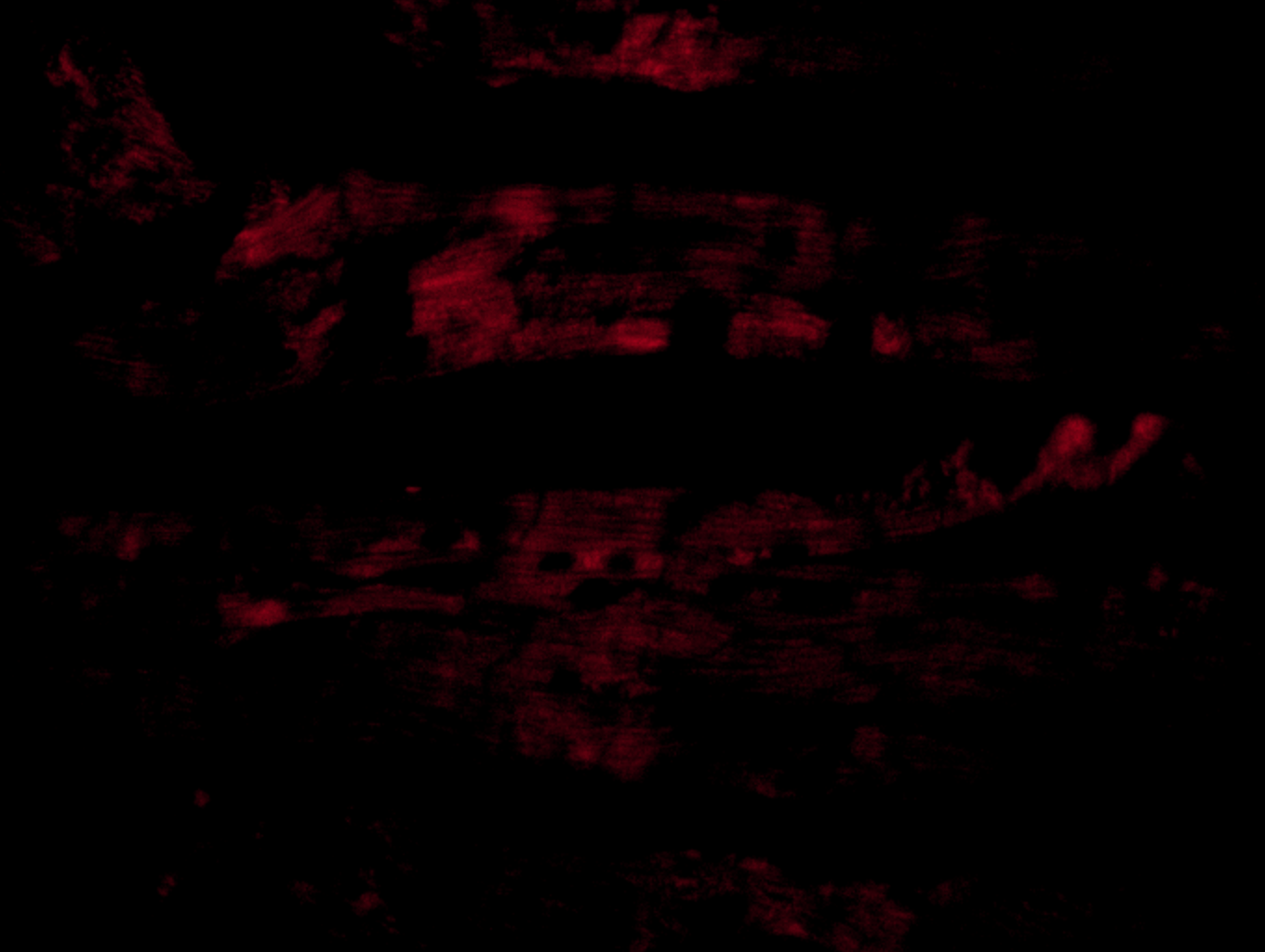

Supplement: Supplementary file 11 [file DataSheet12.ZIP › Fig5一/Fig5I,K/IR+MK AKT.tif]

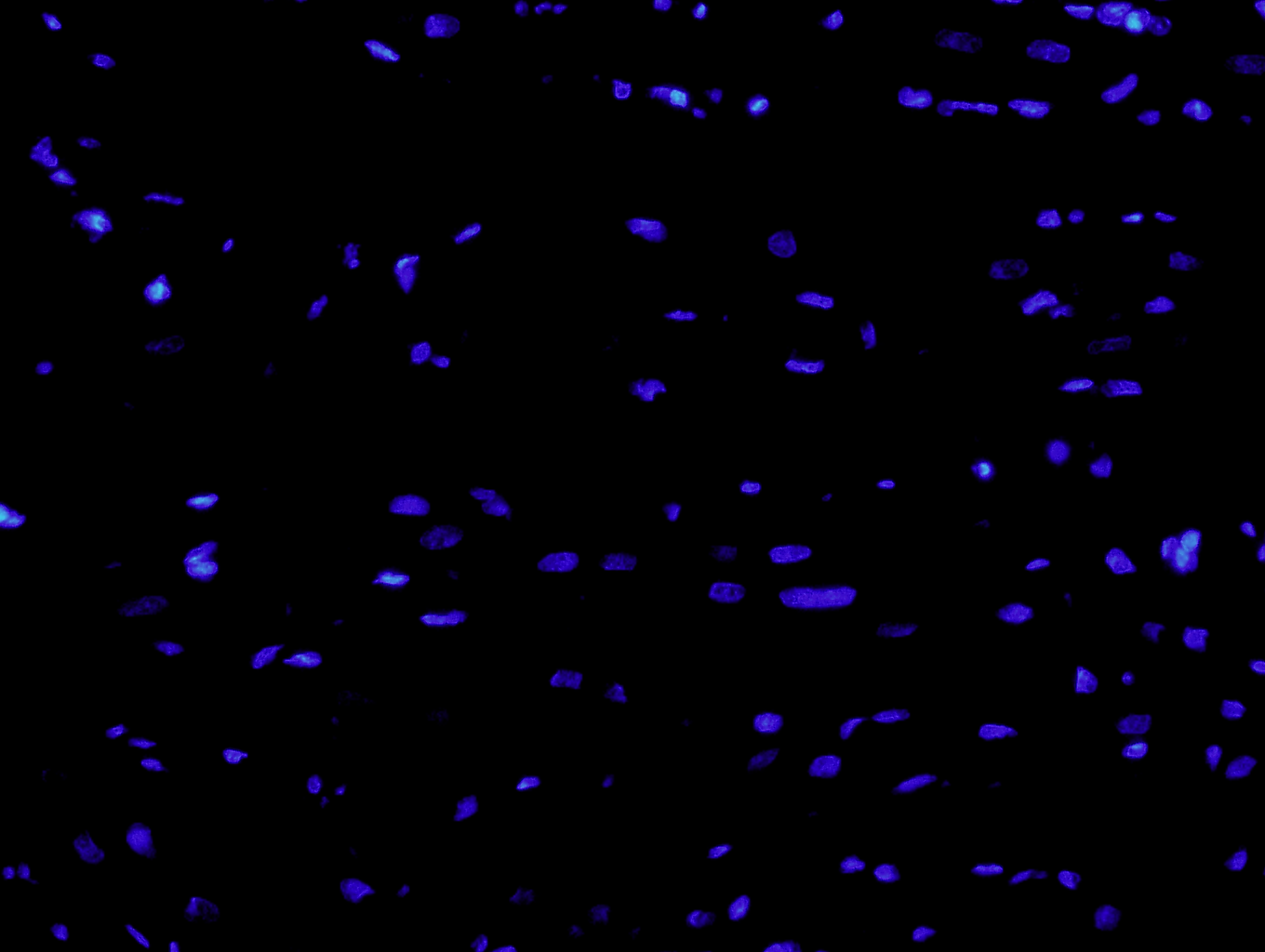

Supplement: Supplementary file 11 [file DataSheet12.ZIP › Fig5一/Fig5I,K/IR+MK dapi.tif]

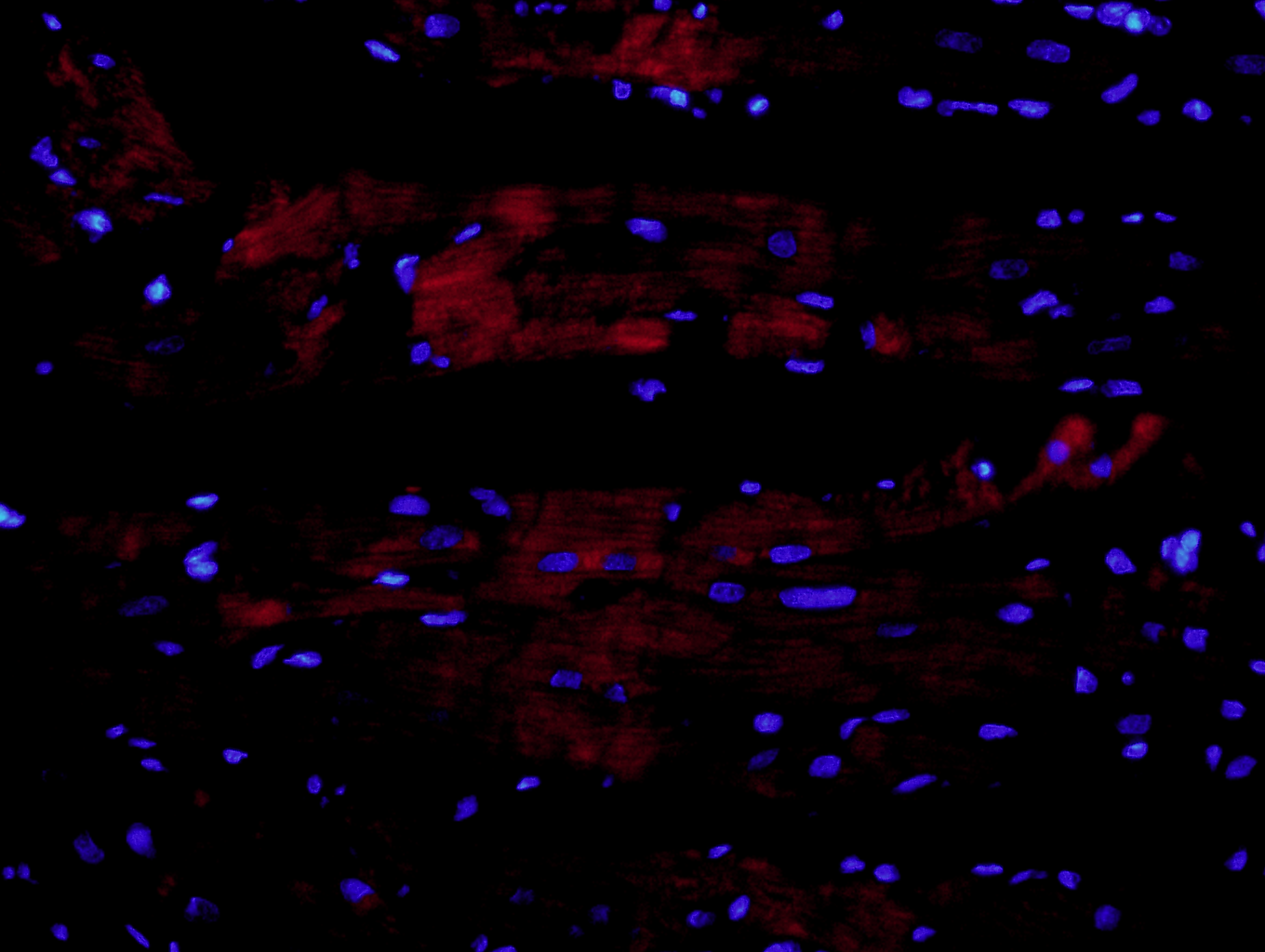

Supplement: Supplementary file 11 [file DataSheet12.ZIP › Fig5一/Fig5I,K/IR+MK merge.tif]

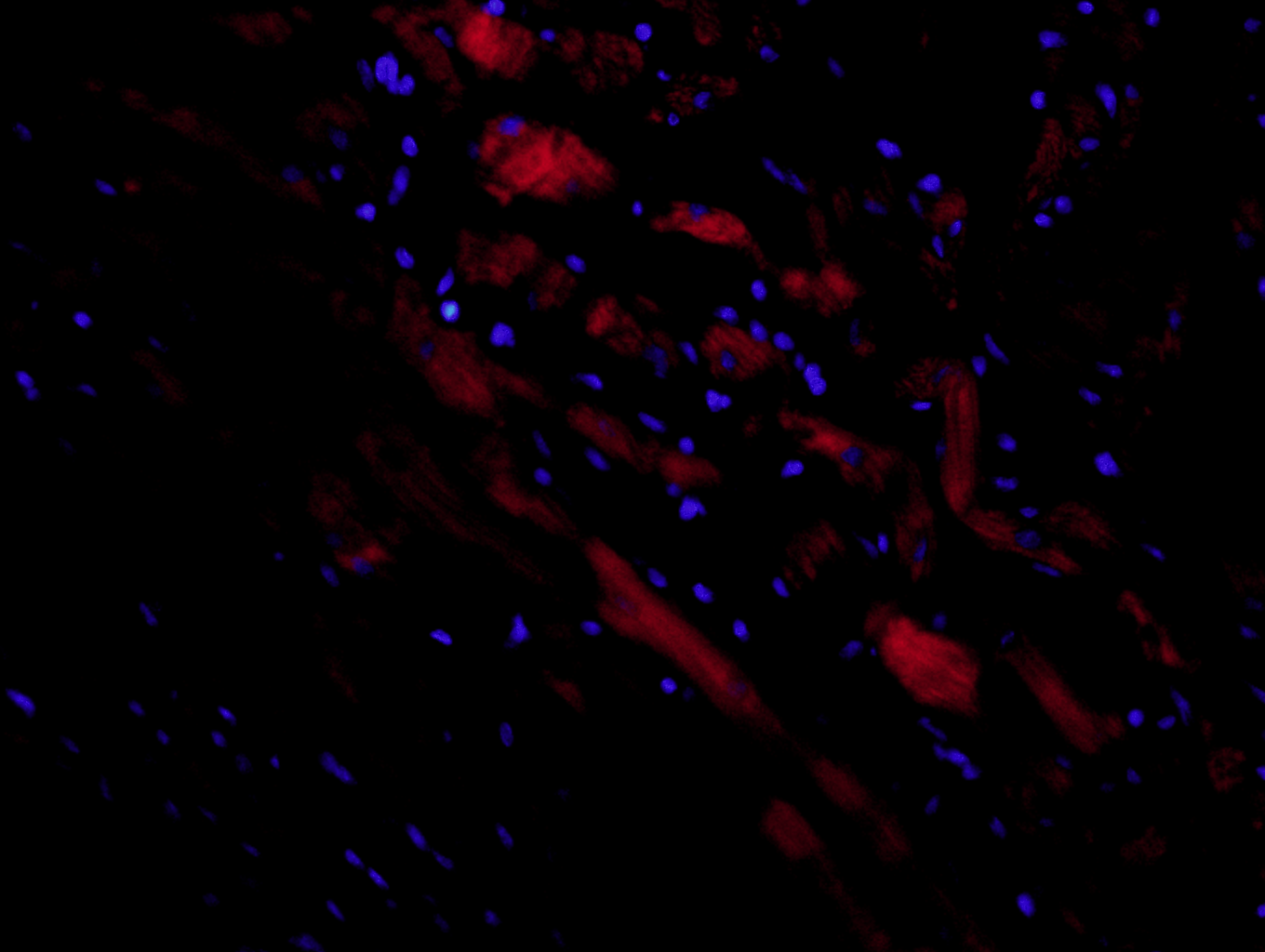

Supplement: Supplementary file 11 [file DataSheet12.ZIP › Fig5一/Fig5I,K/IR+P merge.tif]

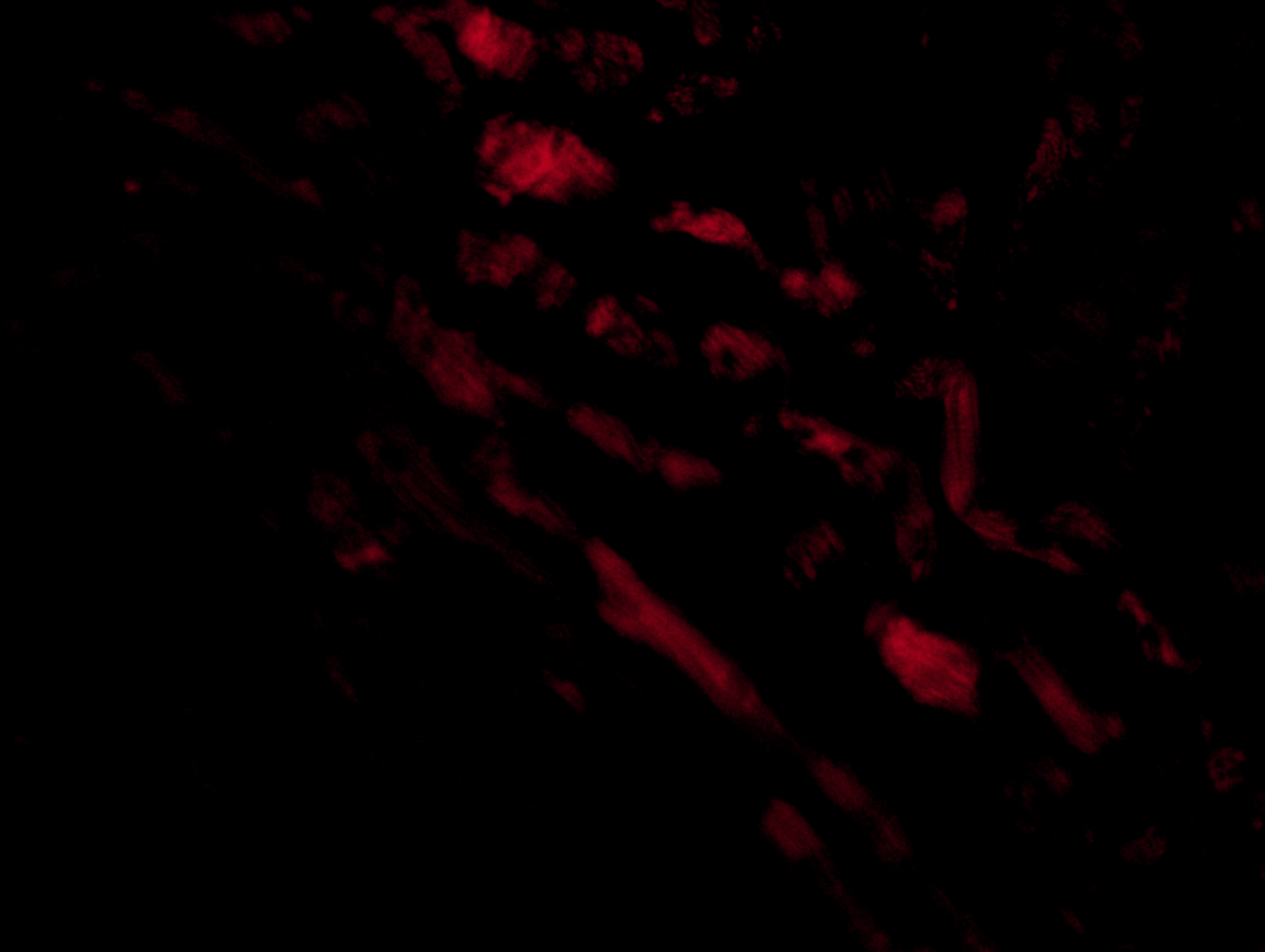

Supplement: Supplementary file 11 [file DataSheet12.ZIP › Fig5一/Fig5I,K/IR+P AKT.tif]

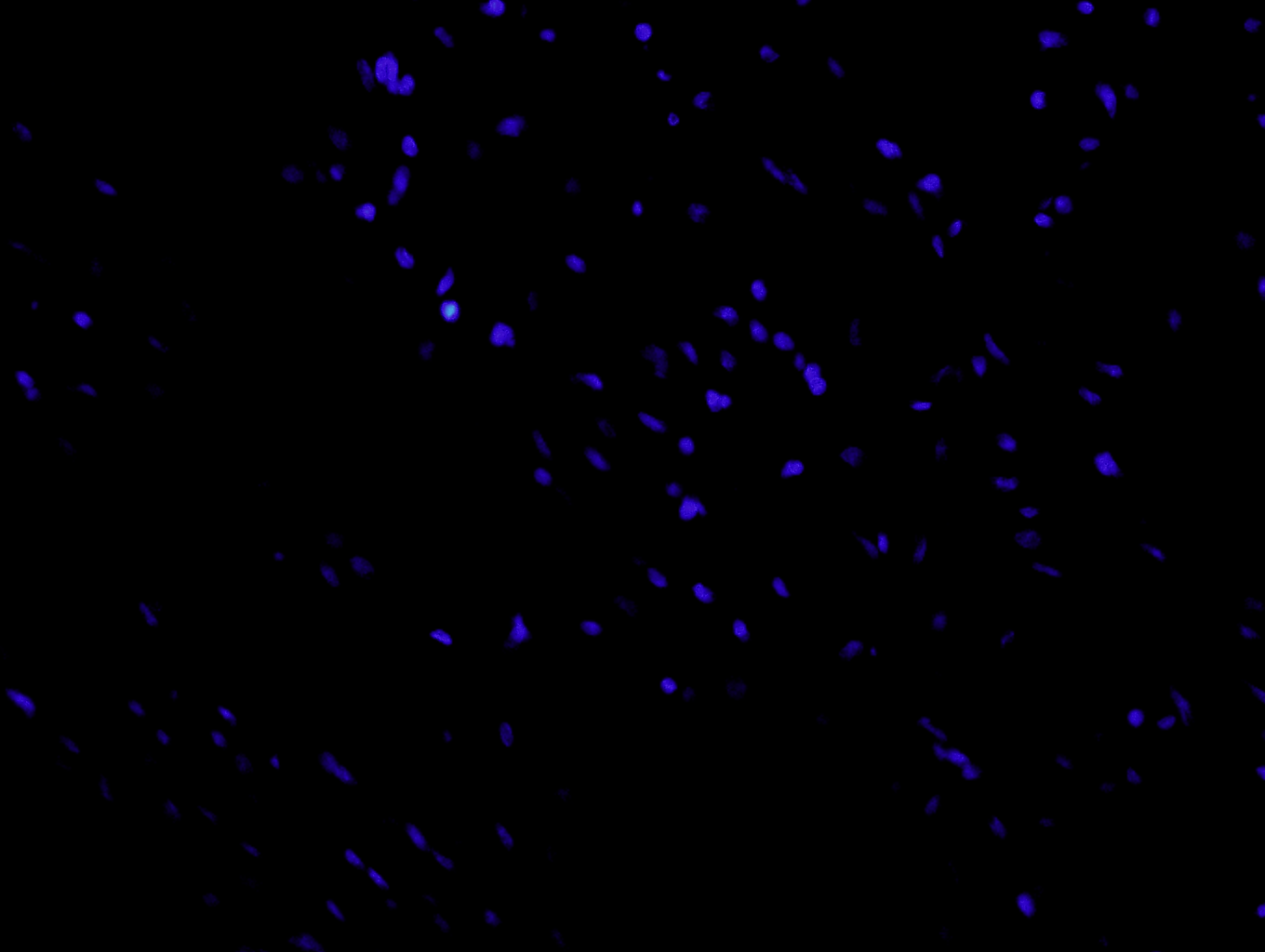

Supplement: Supplementary file 11 [file DataSheet12.ZIP › Fig5一/Fig5I,K/IR+P dapi.tif]

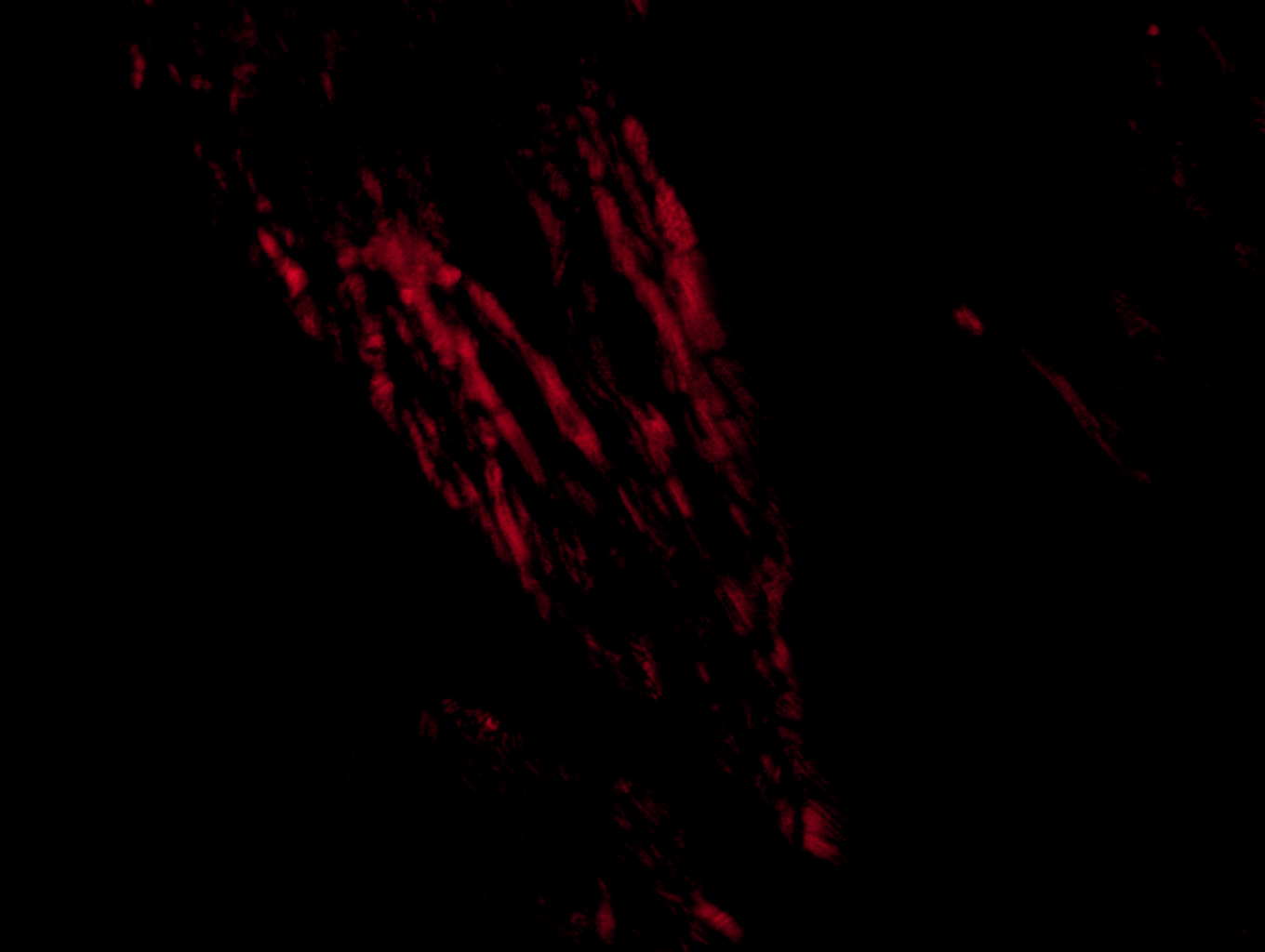

Supplement: Supplementary file 11 [file DataSheet12.ZIP › Fig5一/Fig5I,K/IR+P+mk AKT.tif]

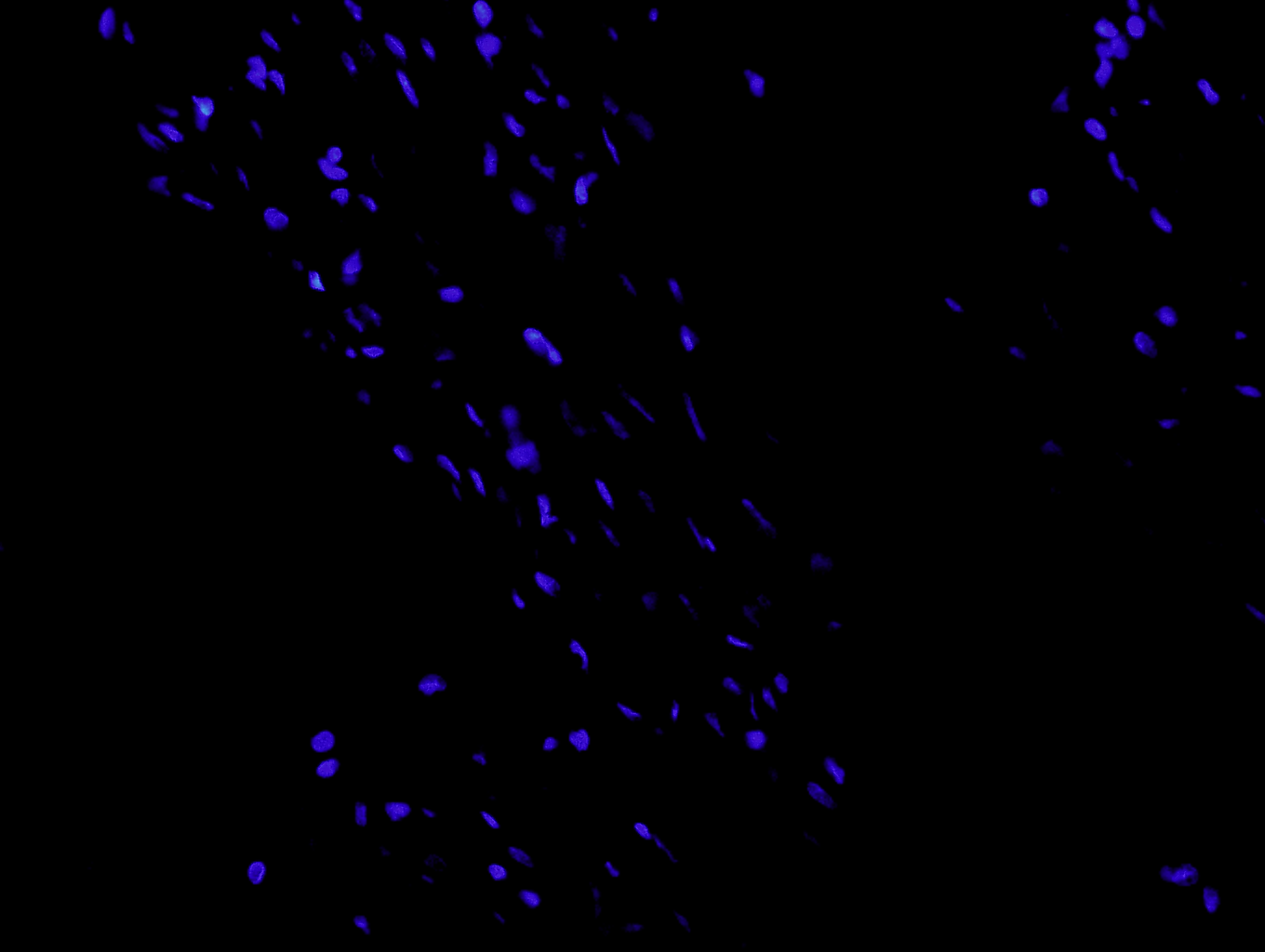

Supplement: Supplementary file 11 [file DataSheet12.ZIP › Fig5一/Fig5I,K/IR+P+mk dapi.tif]

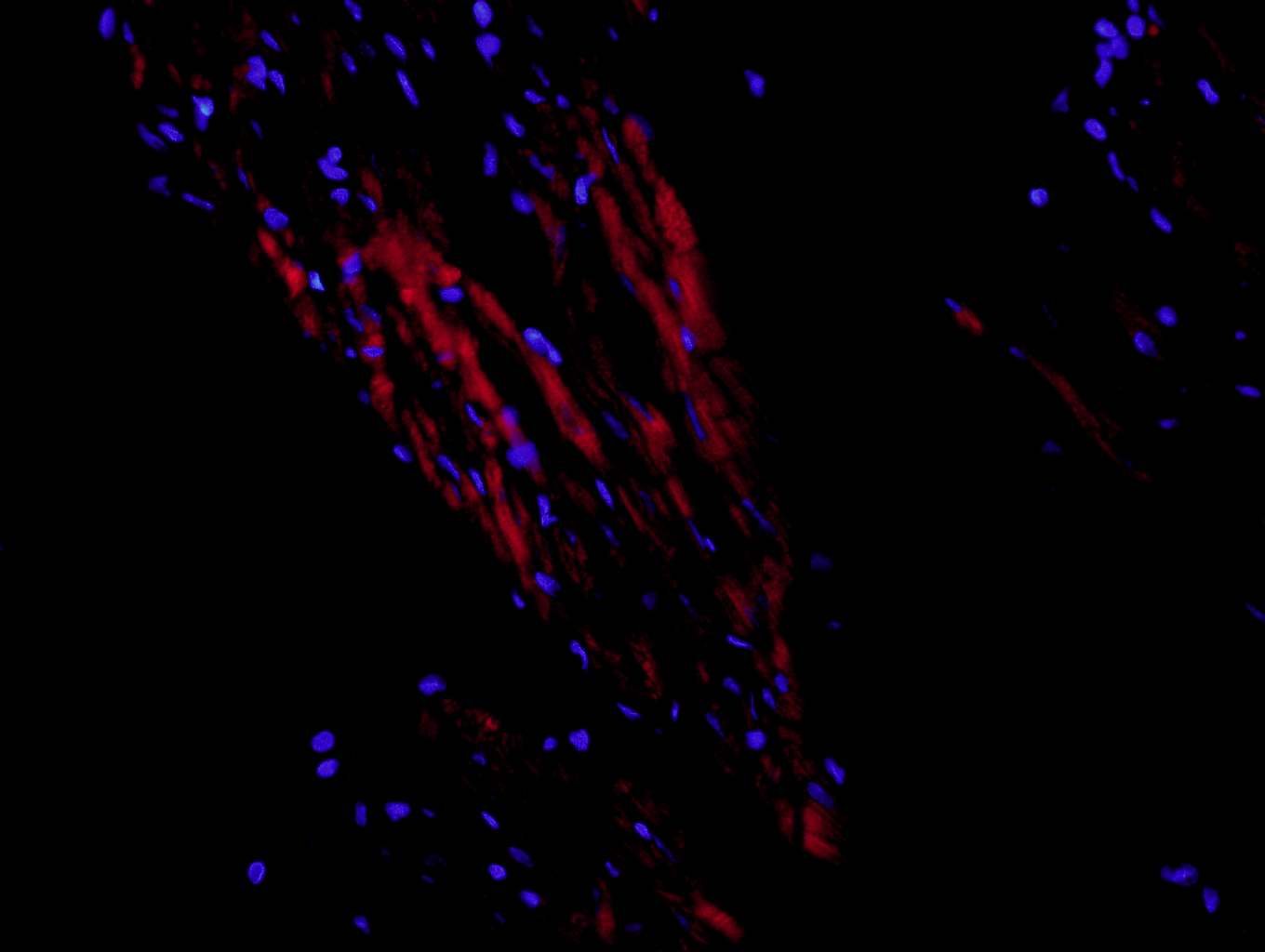

Supplement: Supplementary file 11 [file DataSheet12.ZIP › Fig5一/Fig5I,K/IR+P+mk merge.tif]

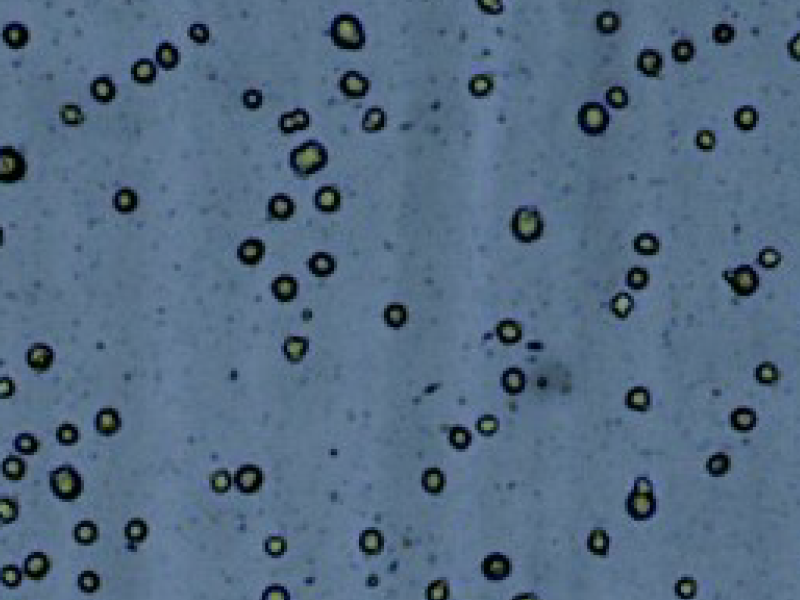

Supplement: Supplementary file 12 [file DataSheet2.ZIP › Fig2/Fig2B,E/Control.tif]

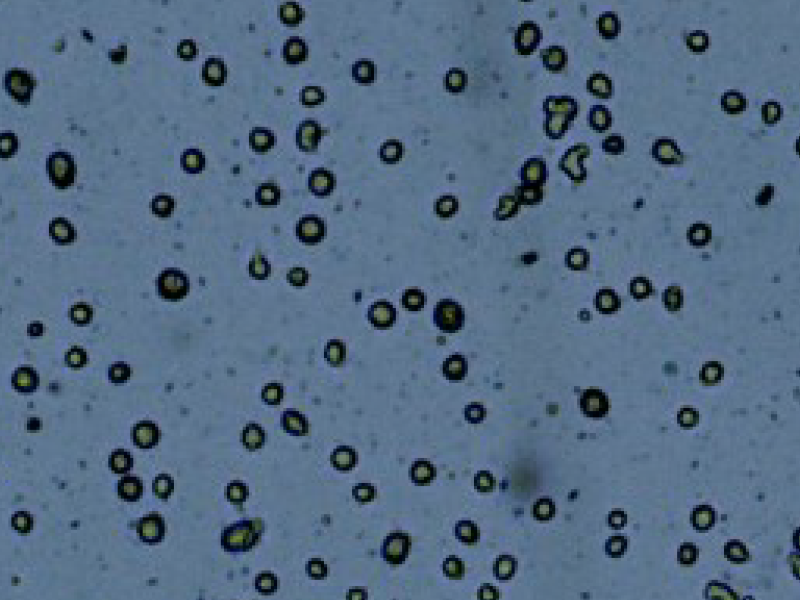

Supplement: Supplementary file 12 [file DataSheet2.ZIP › Fig2/Fig2B,E/E+P.tif]

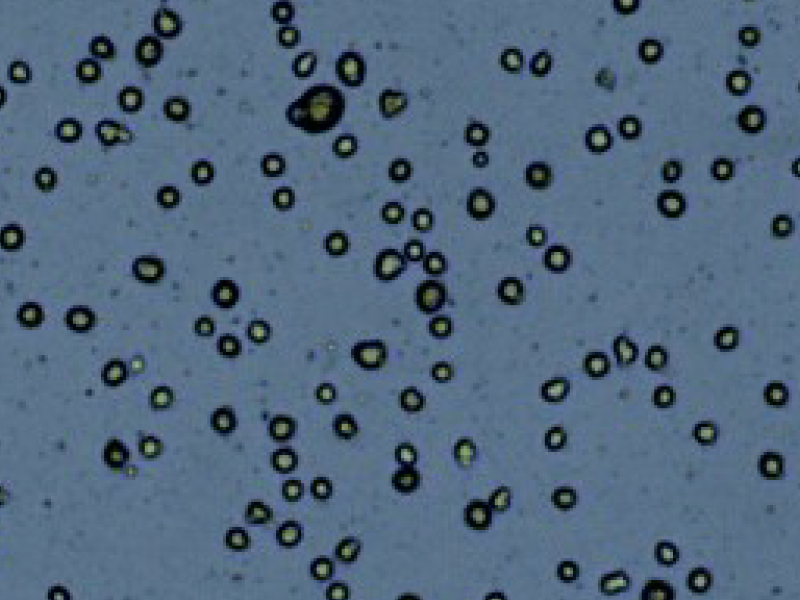

Supplement: Supplementary file 12 [file DataSheet2.ZIP › Fig2/Fig2B,E/E.tif]

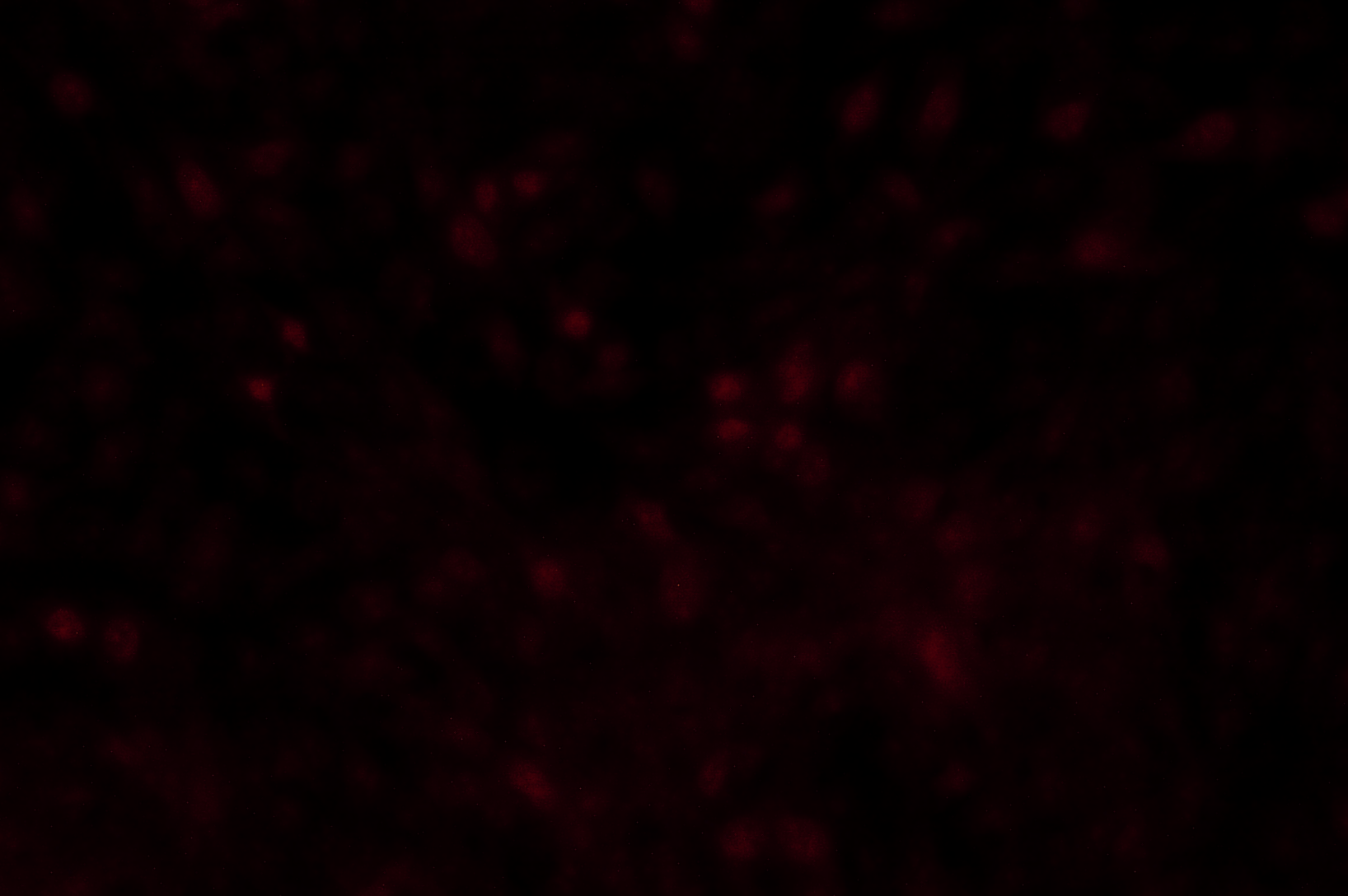

Supplement: Supplementary file 12 [file DataSheet2.ZIP › Fig2/Fig2C,F/Control.tif]

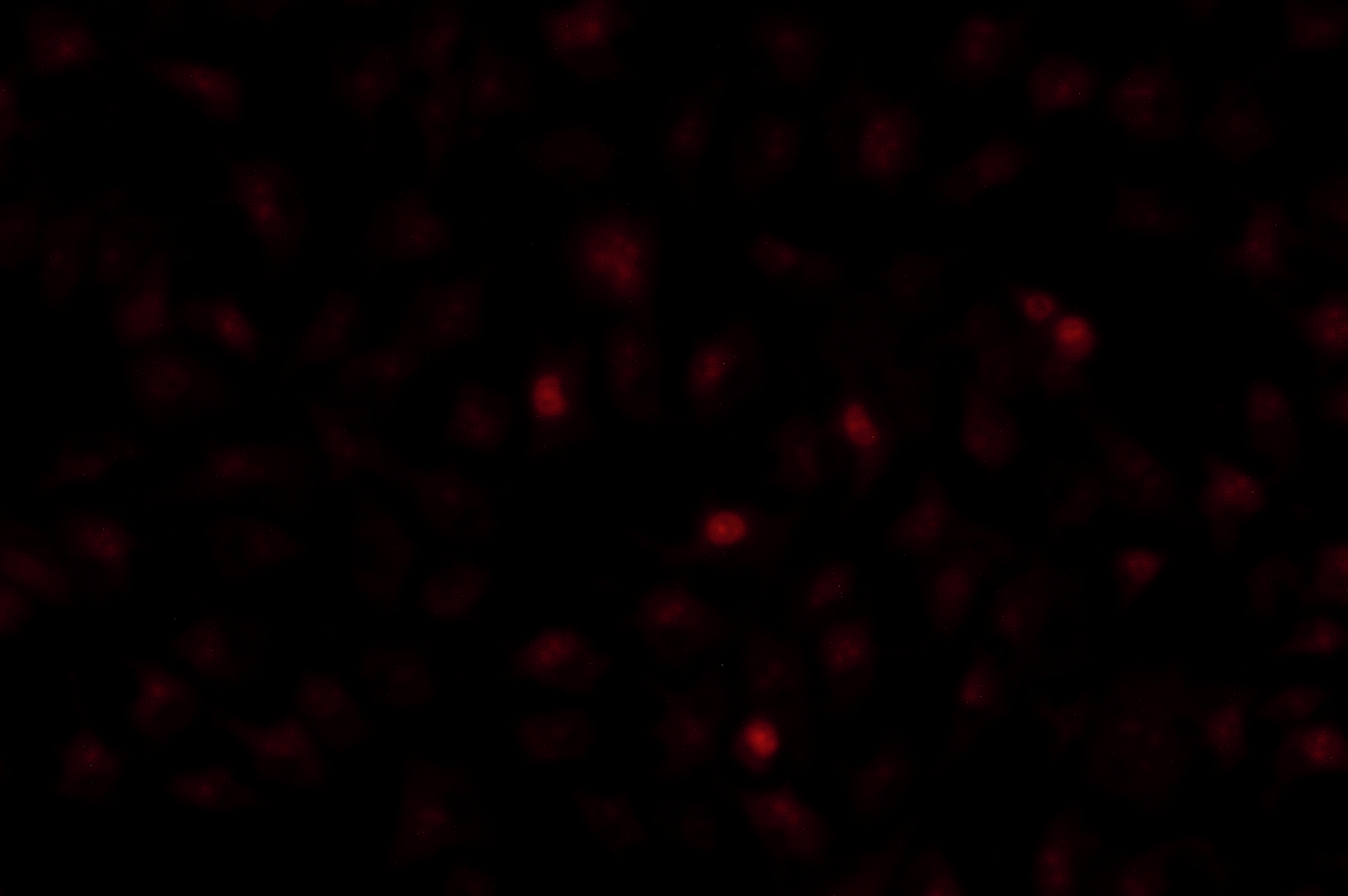

Supplement: Supplementary file 12 [file DataSheet2.ZIP › Fig2/Fig2C,F/E+P.tif]

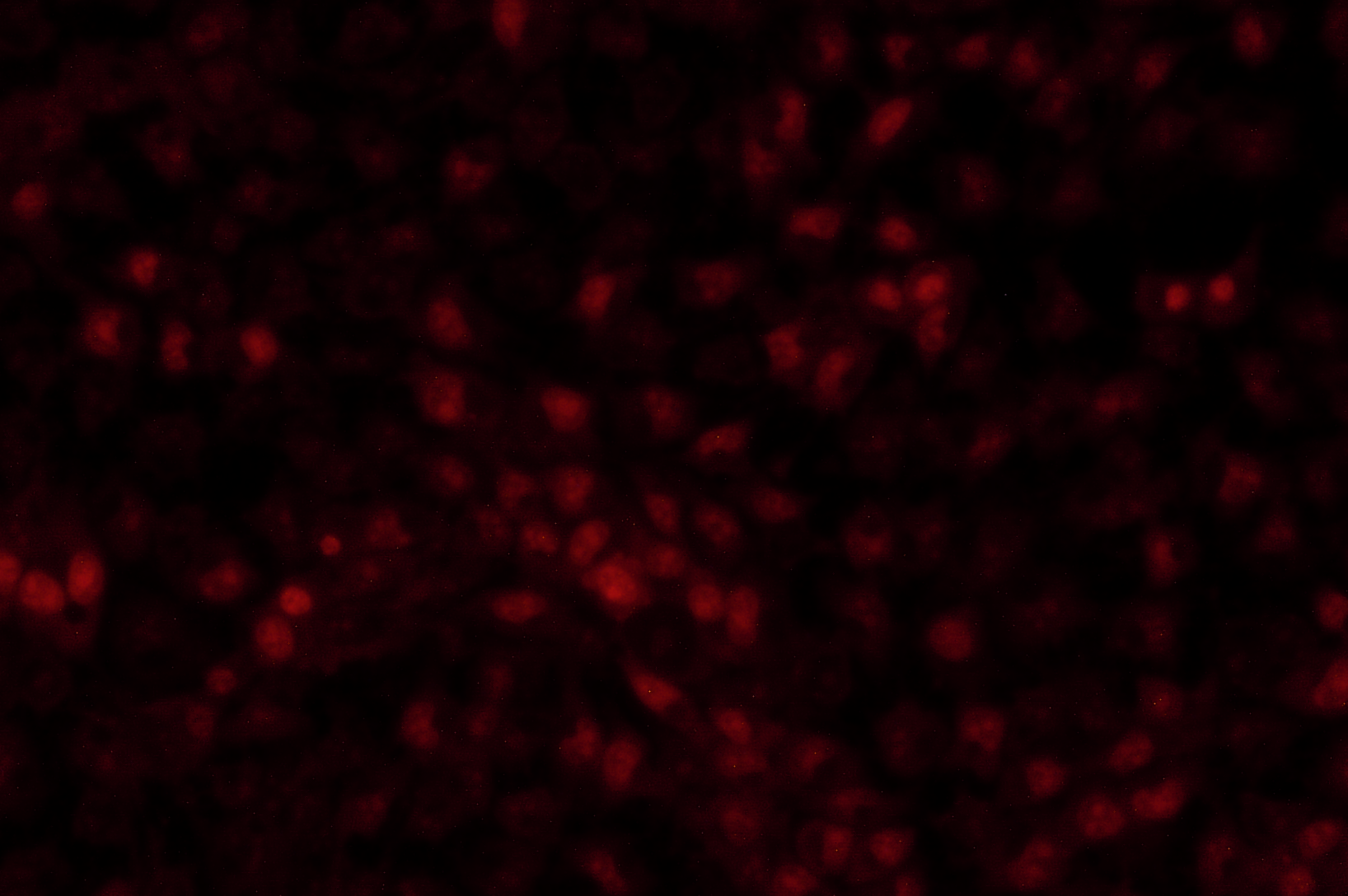

Supplement: Supplementary file 12 [file DataSheet2.ZIP › Fig2/Fig2C,F/E.tif]

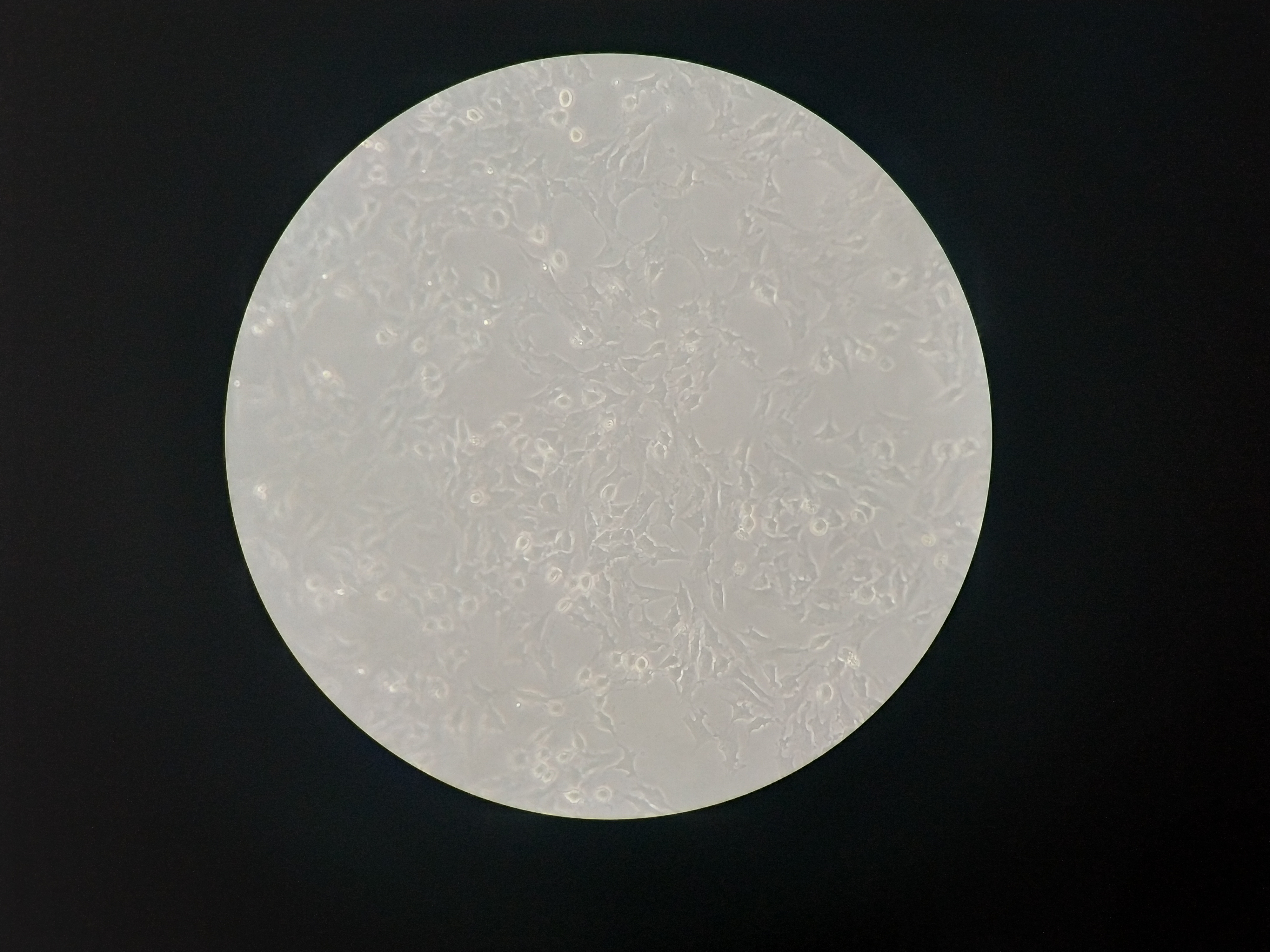

Supplement: Supplementary file 12 [file DataSheet2.ZIP › Fig2/Fig2D/Control.jpg]

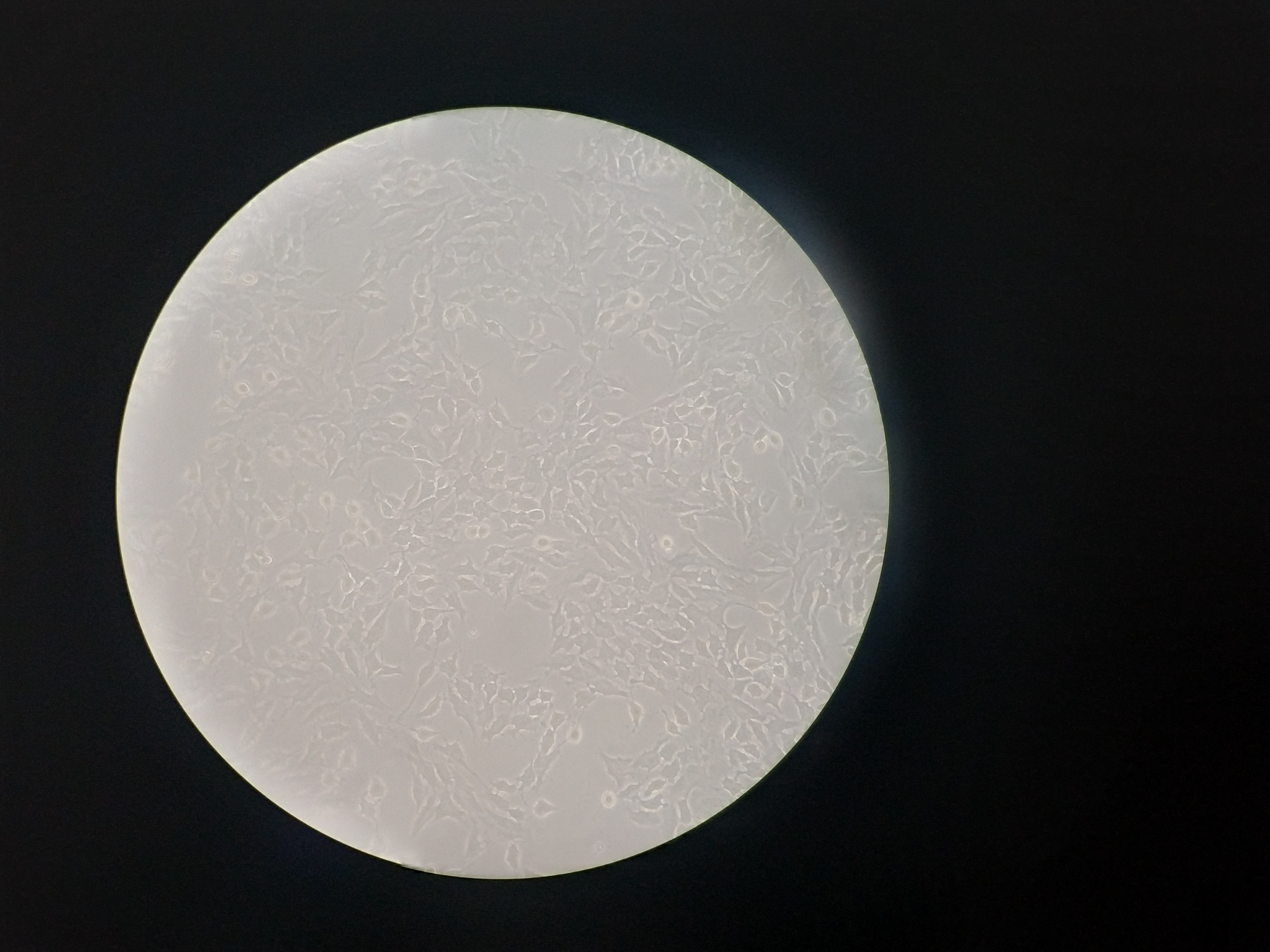

Supplement: Supplementary file 12 [file DataSheet2.ZIP › Fig2/Fig2D/E+P.jpg]

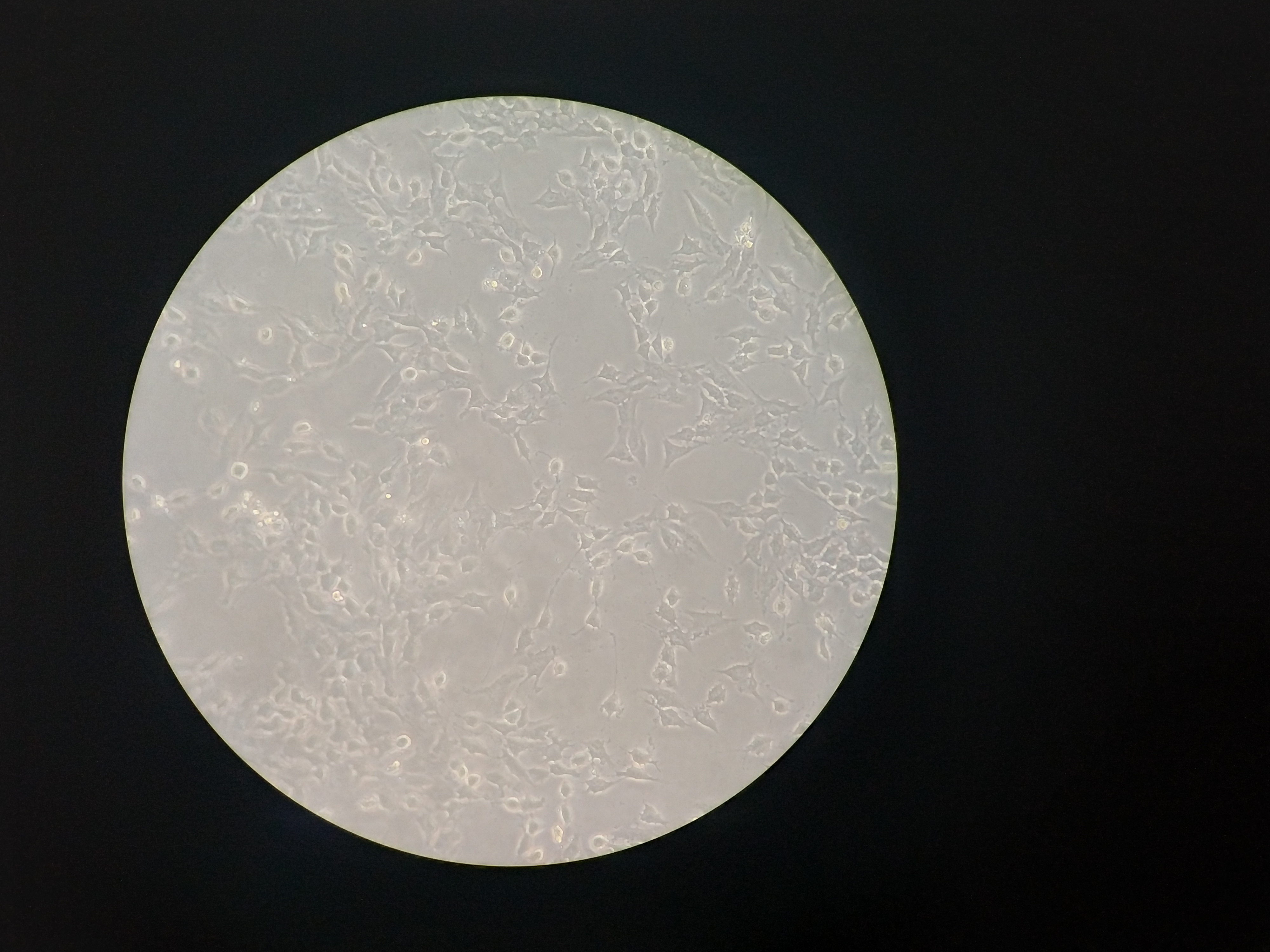

Supplement: Supplementary file 12 [file DataSheet2.ZIP › Fig2/Fig2D/E.jpg]

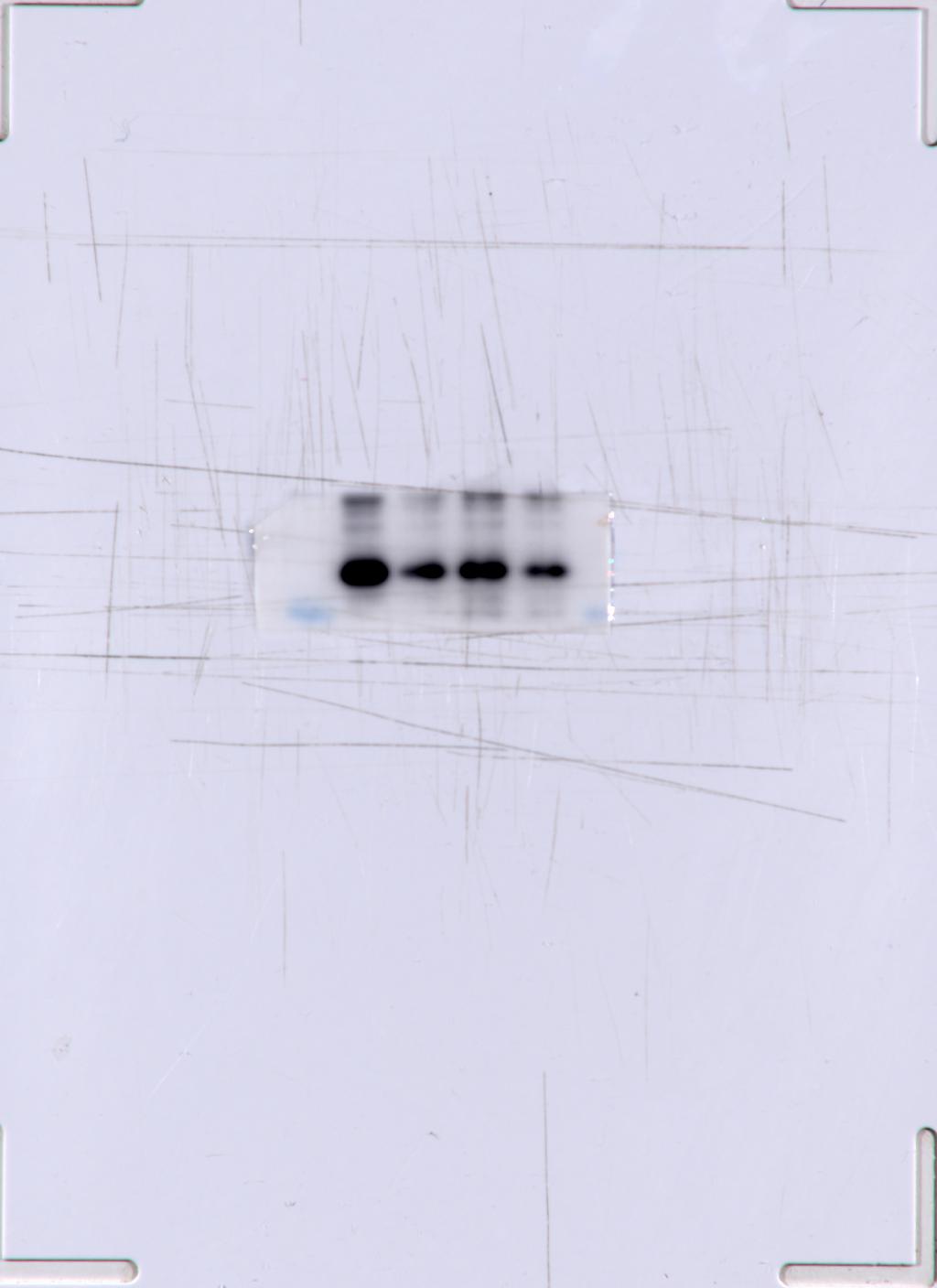

Supplement: Supplementary file 12 [file DataSheet2.ZIP › Fig2/Fig2I-L/FTH1.jpg]

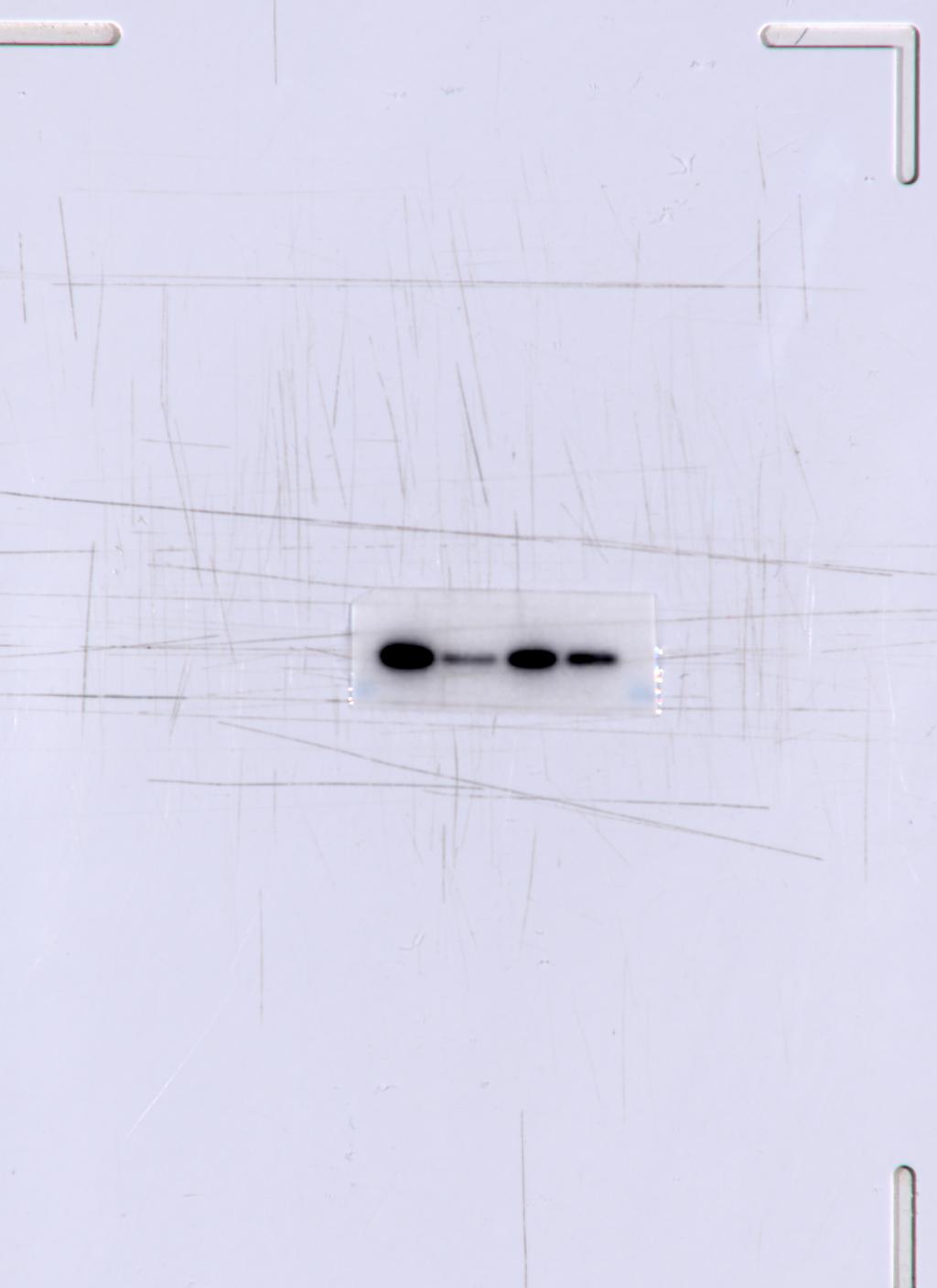

Supplement: Supplementary file 12 [file DataSheet2.ZIP › Fig2/Fig2I-L/GPX4.jpg]

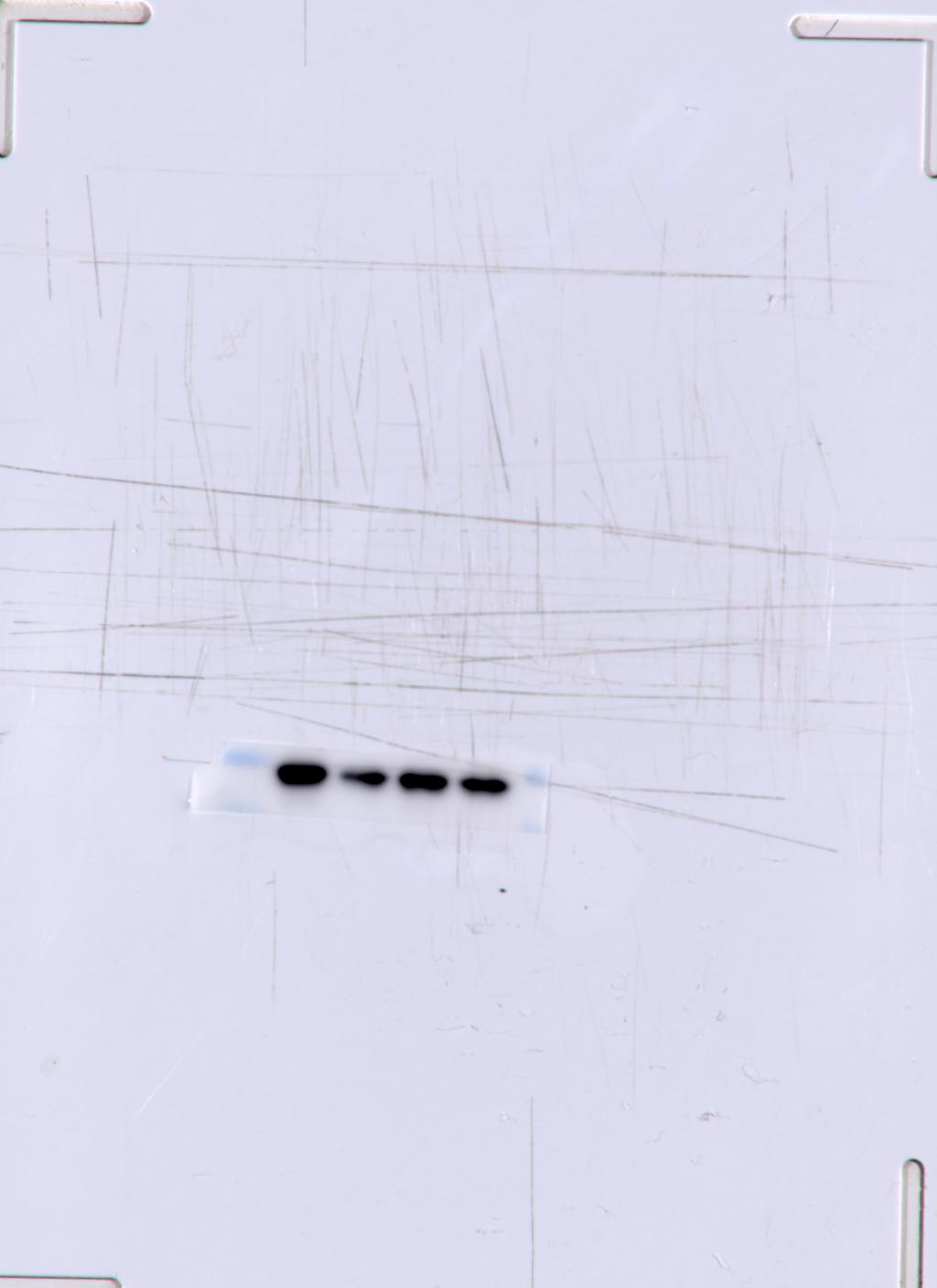

Supplement: Supplementary file 12 [file DataSheet2.ZIP › Fig2/Fig2I-L/SOD-2.jpg]

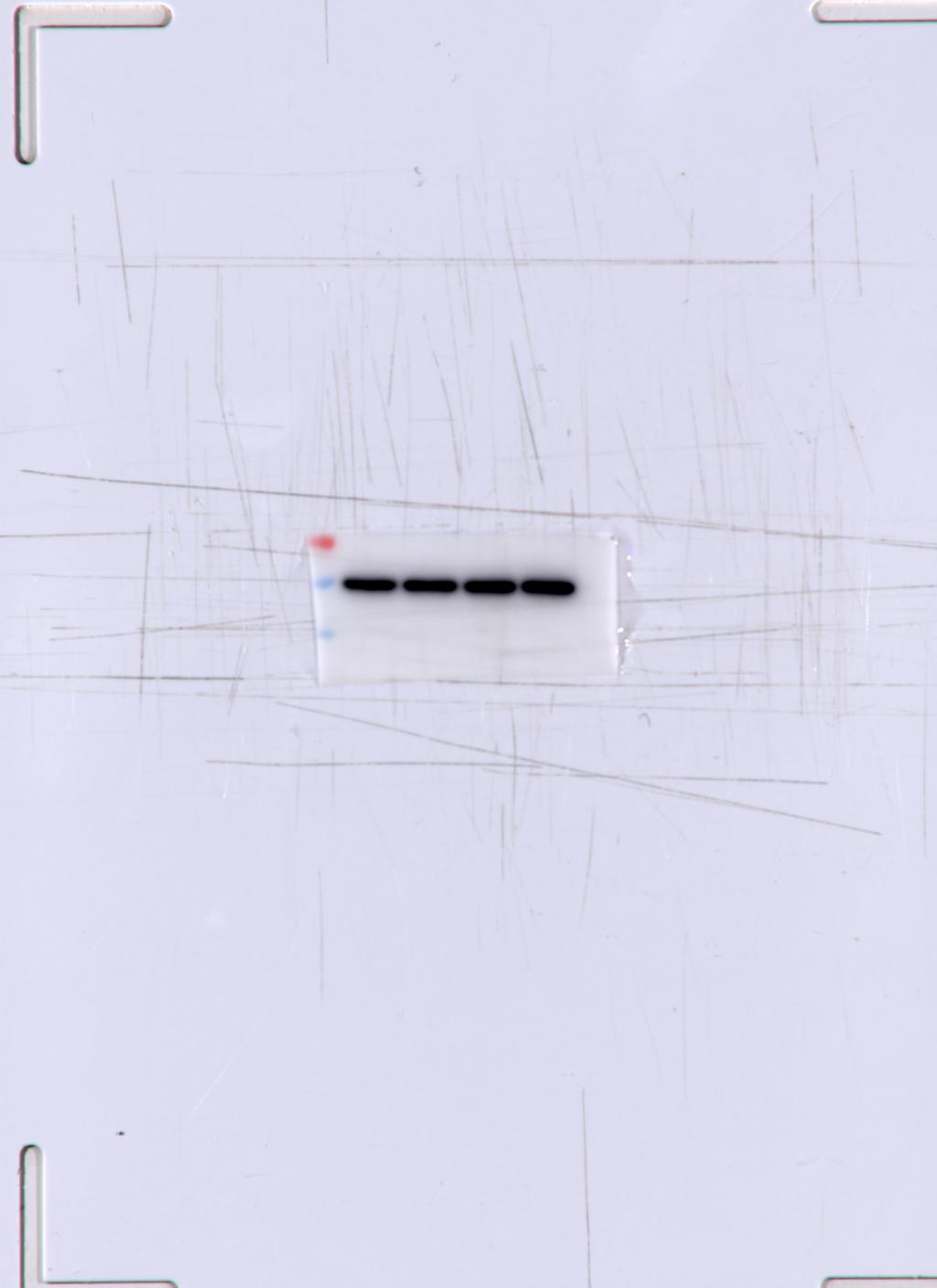

Supplement: Supplementary file 12 [file DataSheet2.ZIP › Fig2/Fig2I-L/α-tubulin.jpg]

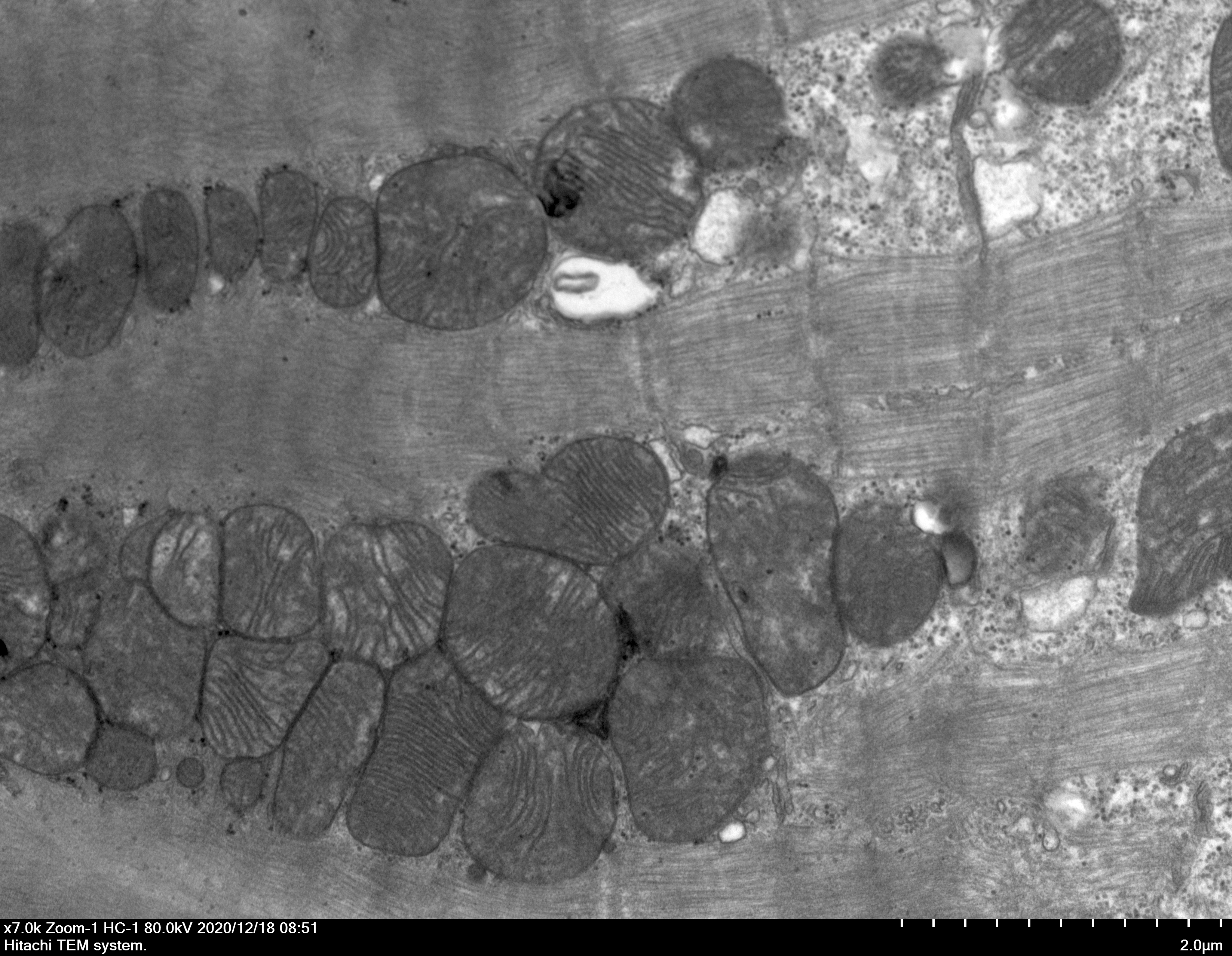

Supplement: Supplementary file 14 [file DataSheet5.ZIP › Control.tif]

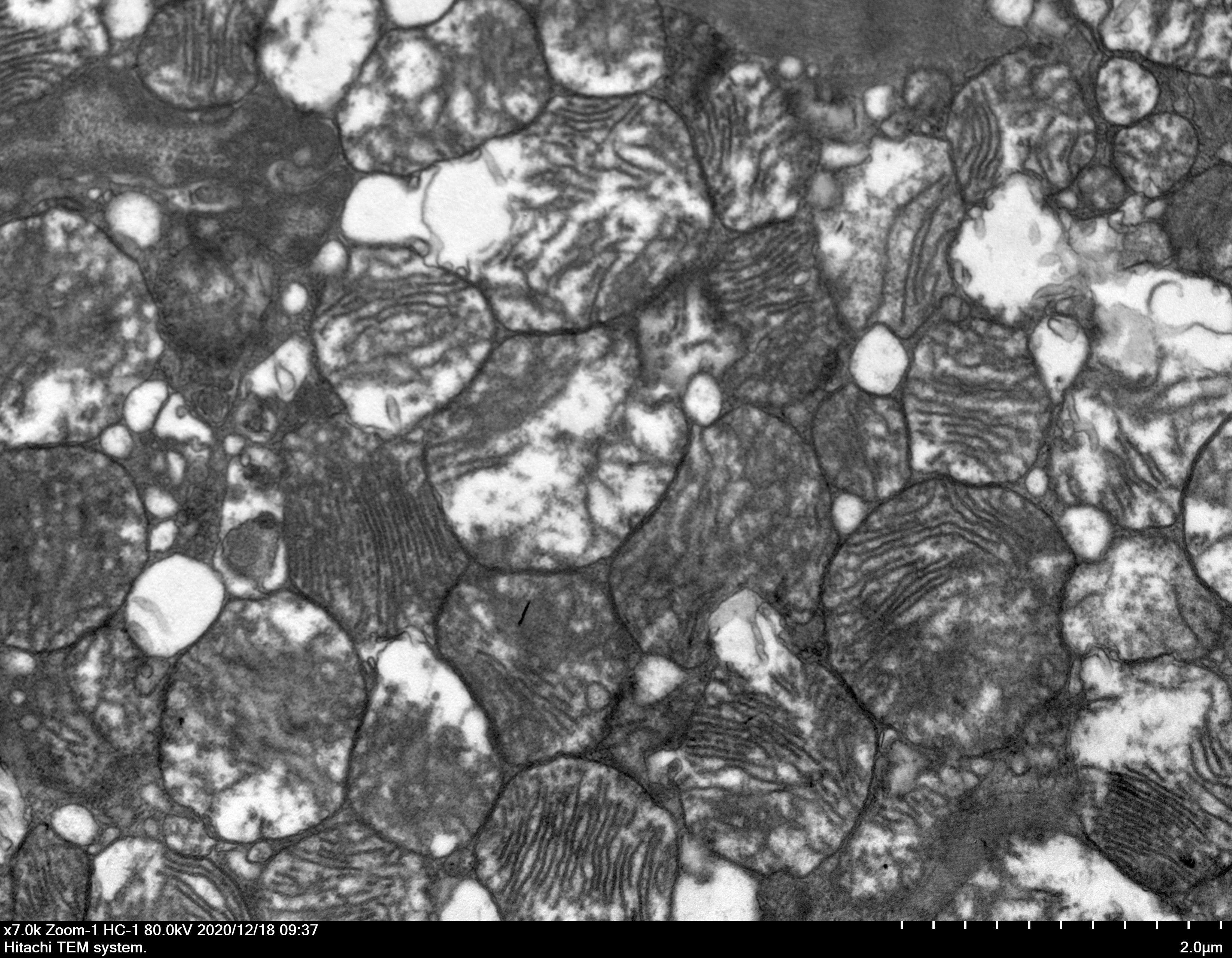

Supplement: Supplementary file 15 [file DataSheet7.ZIP › IR+MK.tif]
