# Supplementary material for: Propofol Protects Myocardium From Ischemia/Reperfusion Injury by Inhibiting Ferroptosis Through the AKT/p53 Signaling Pathway
Source: Front Pharmacol. 2022 Mar 16;13:841410. doi: 10.3389/fphar.2022.841410 (PMC8966655; doi:10.3389/fphar.2022.841410)

**Supplemental figure S1**: Full scan of the original blots of cropped images shown in Figure 4 C.

Lane1 and lane5: marker. Lane2:C. Lane3: I/R. Lane4: I/R+P.

FTH1 XCT


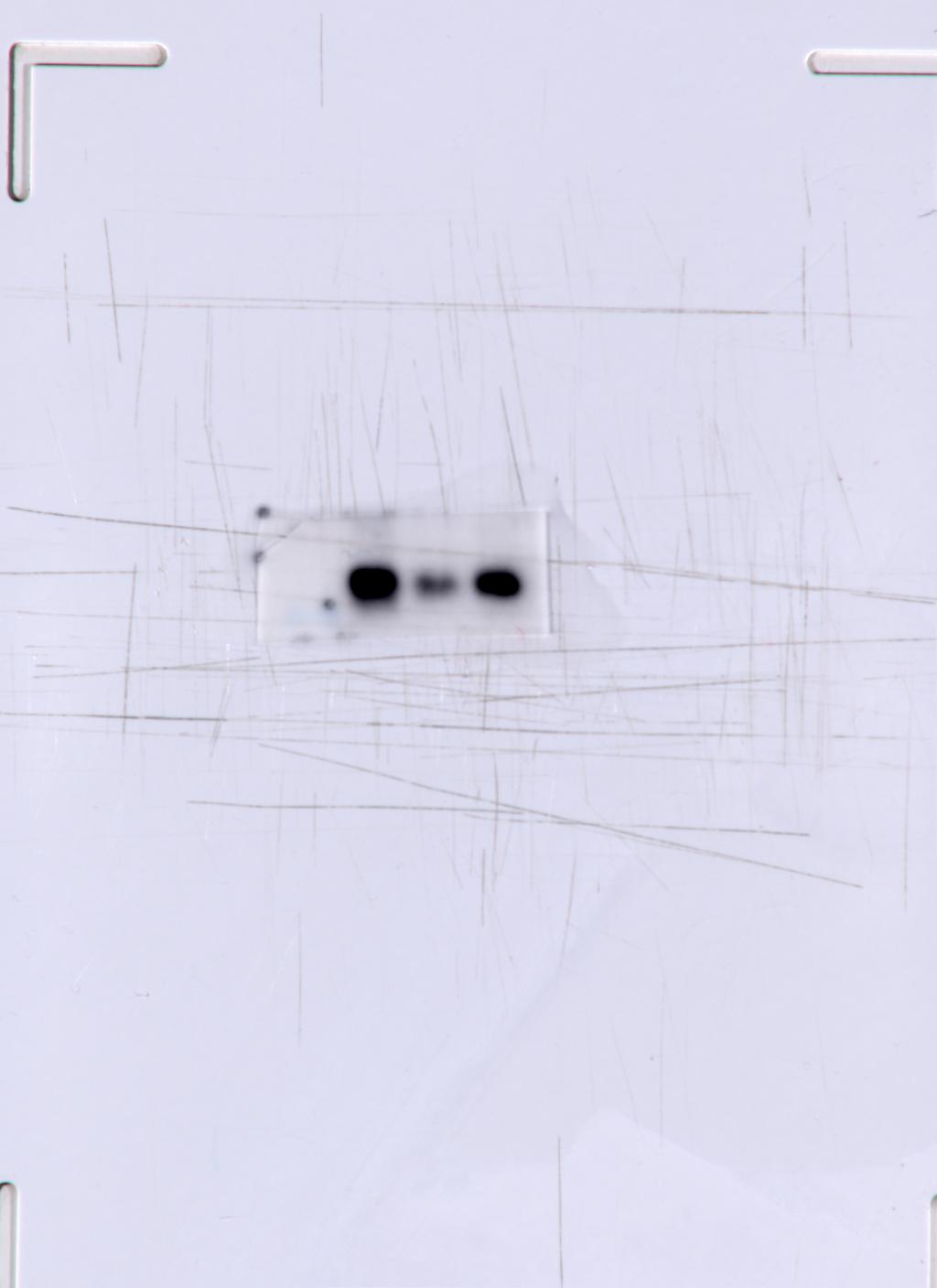

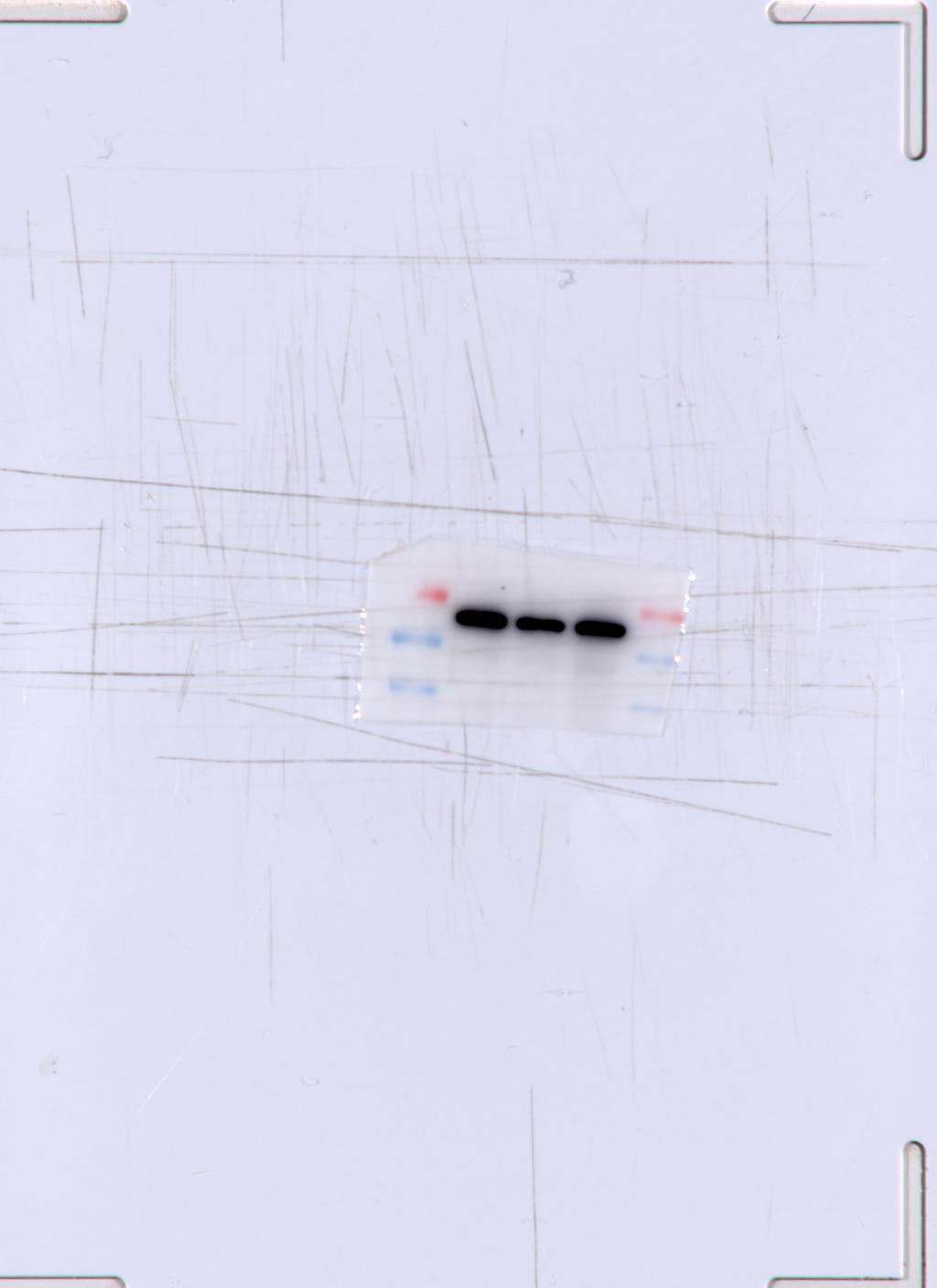


SOD-2 GPX4


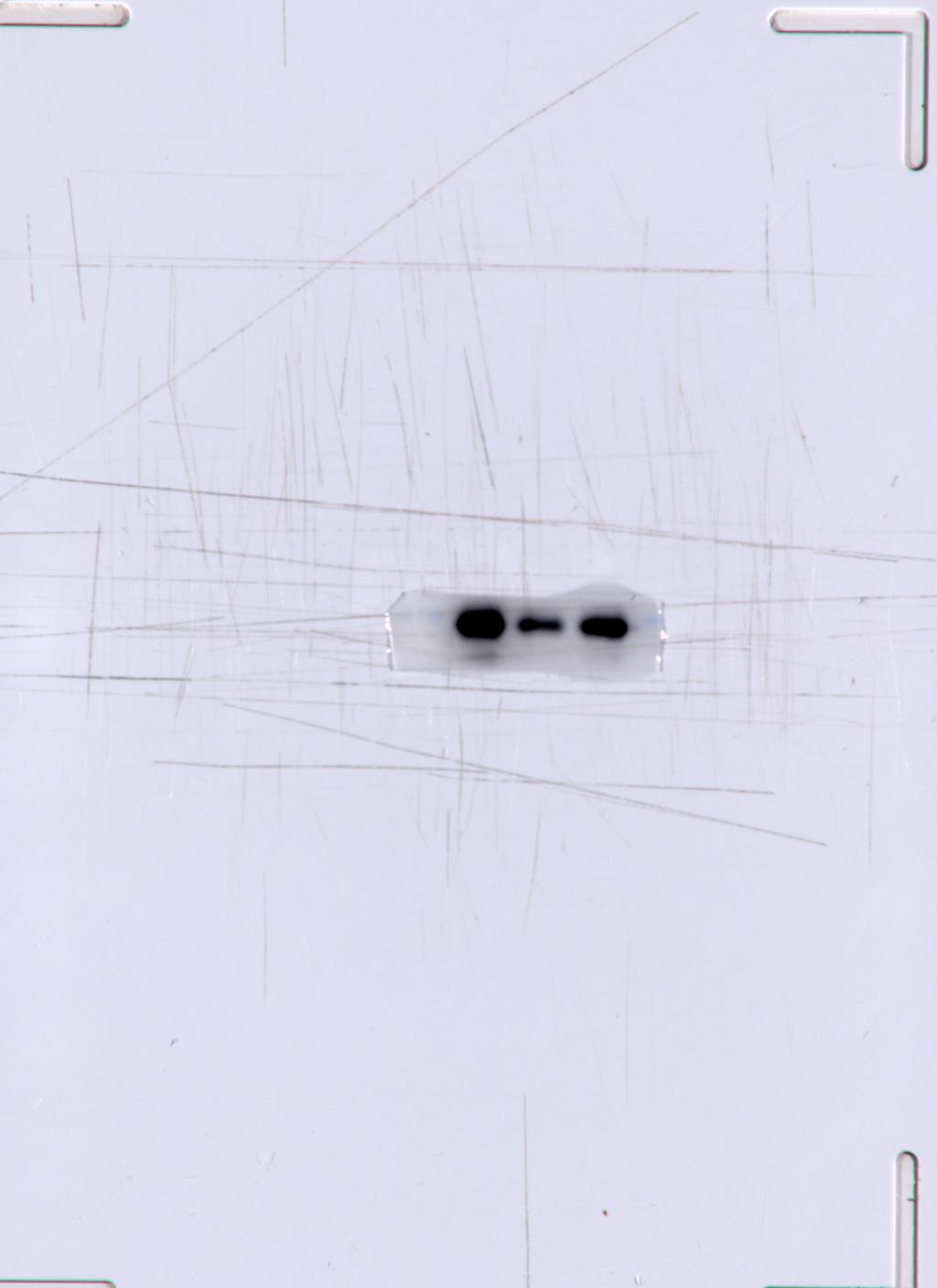

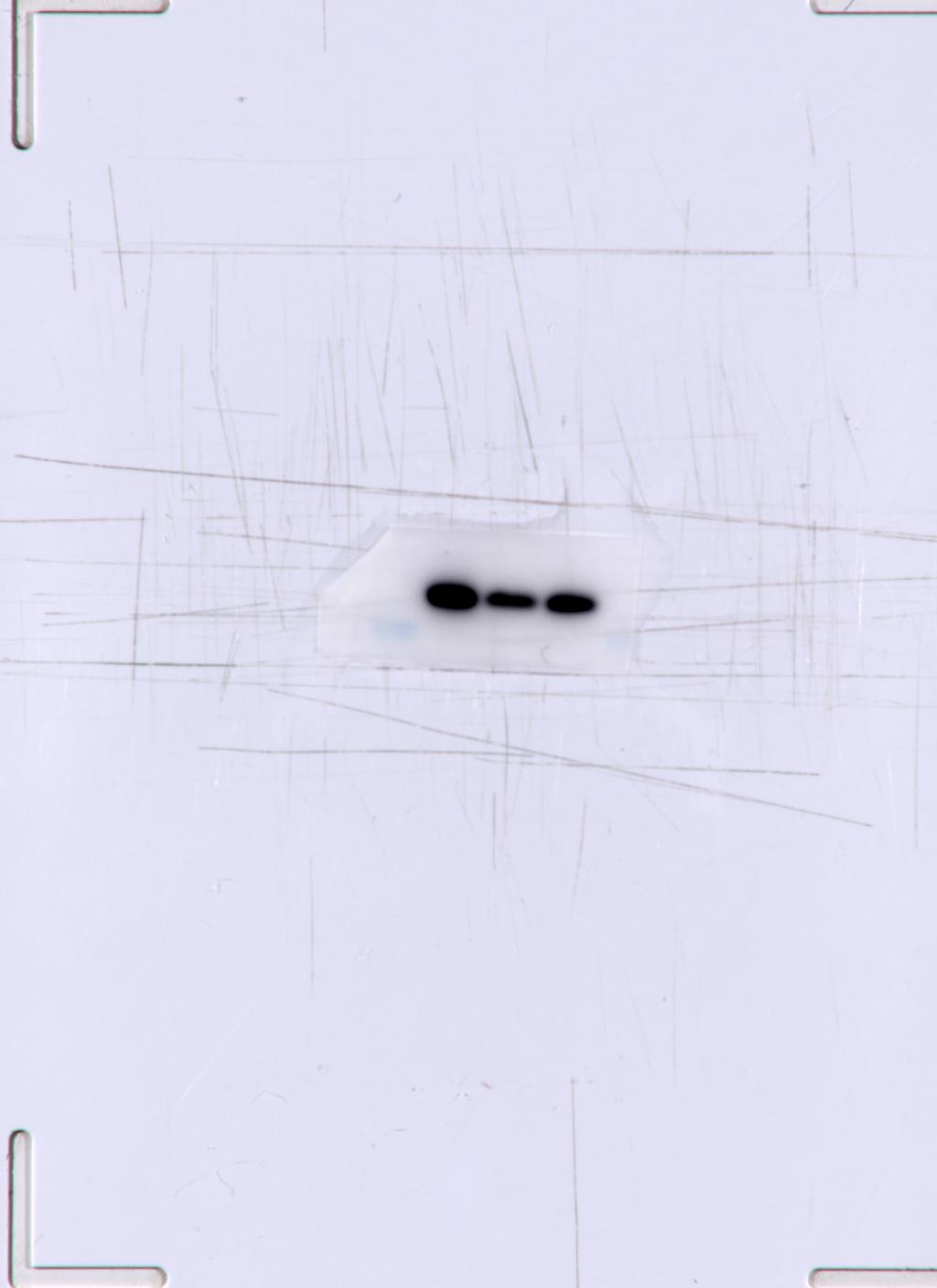


α-tubulin


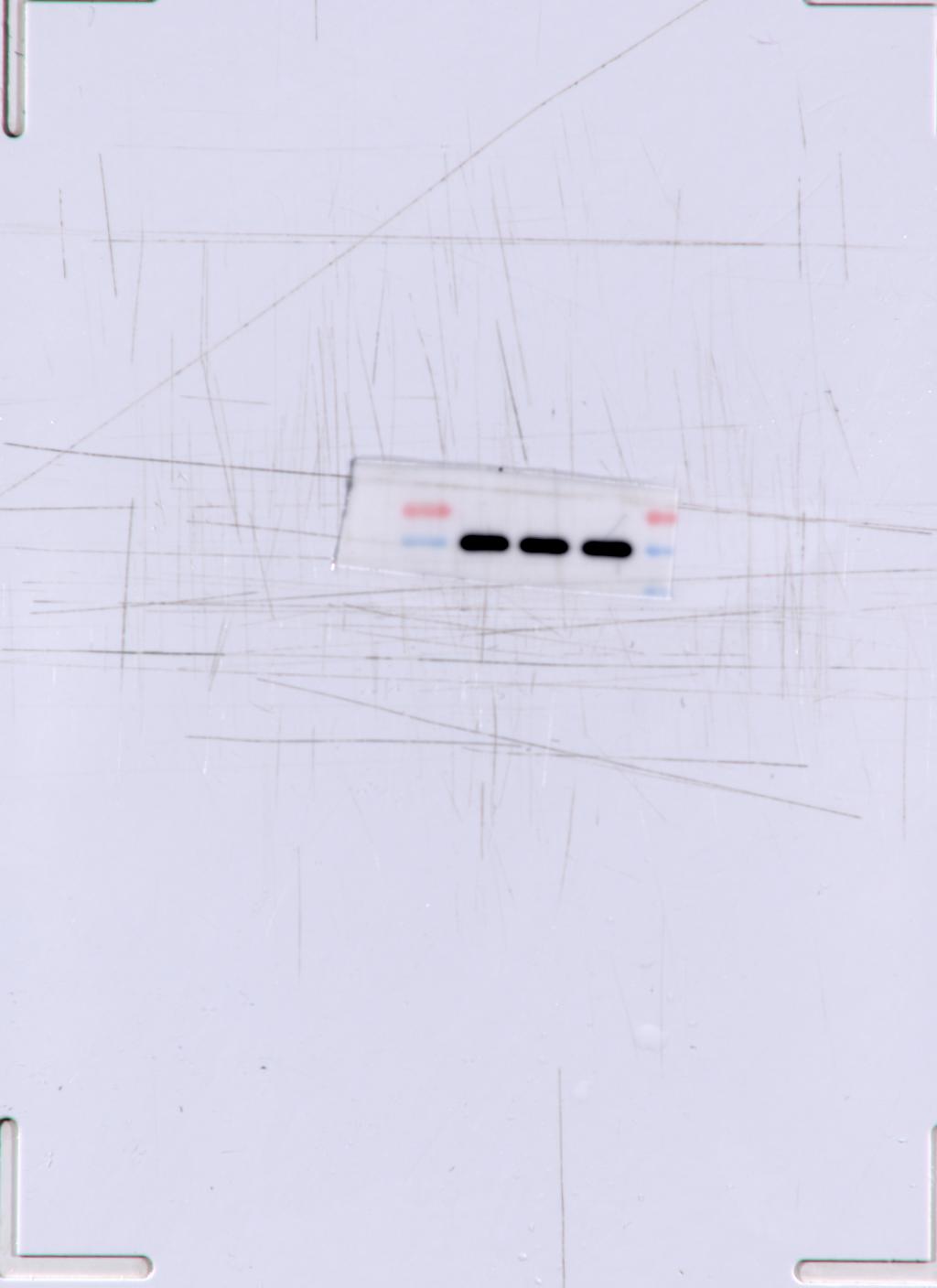


**Supplemental figure S2**: Full scan of the original blots of cropped images shown in Figure 4 L.

Lane1,7: marker. Lane2: C. Lane3: I/R. Lane4: I/R+P. Lane5: I/R+P+MK. Lane6: I/R+MK.

p-p53 p53


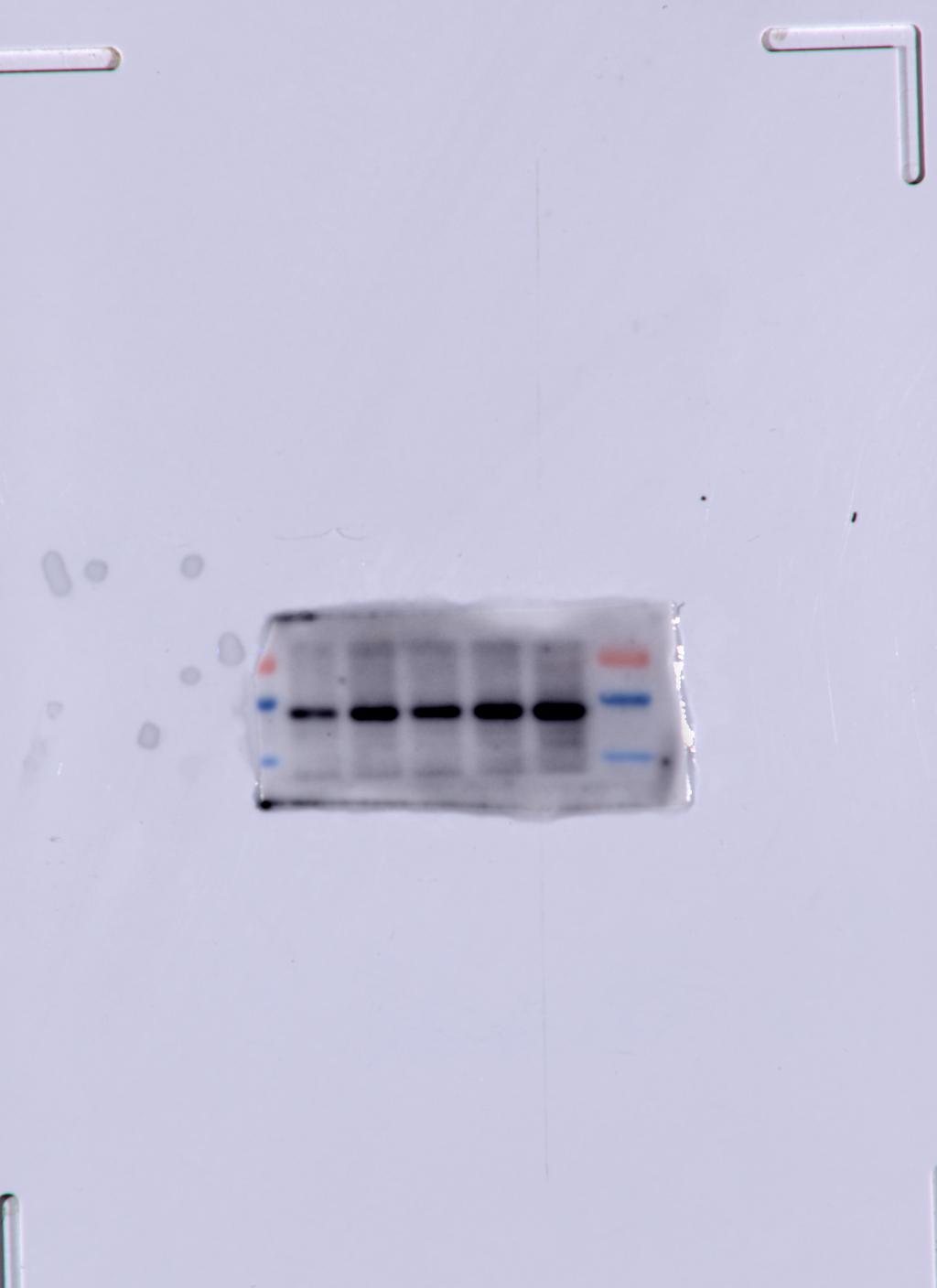

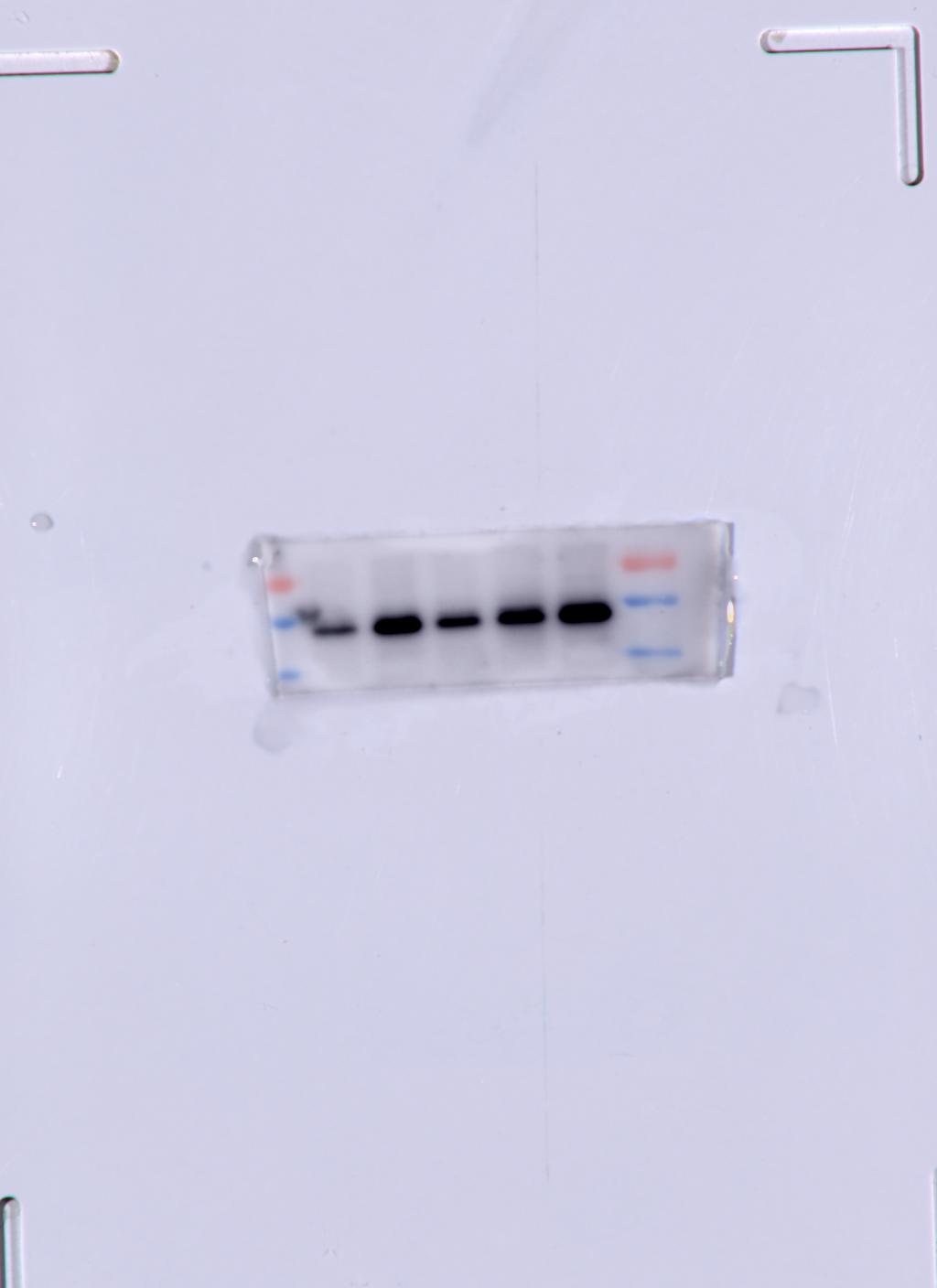


α-tubulin


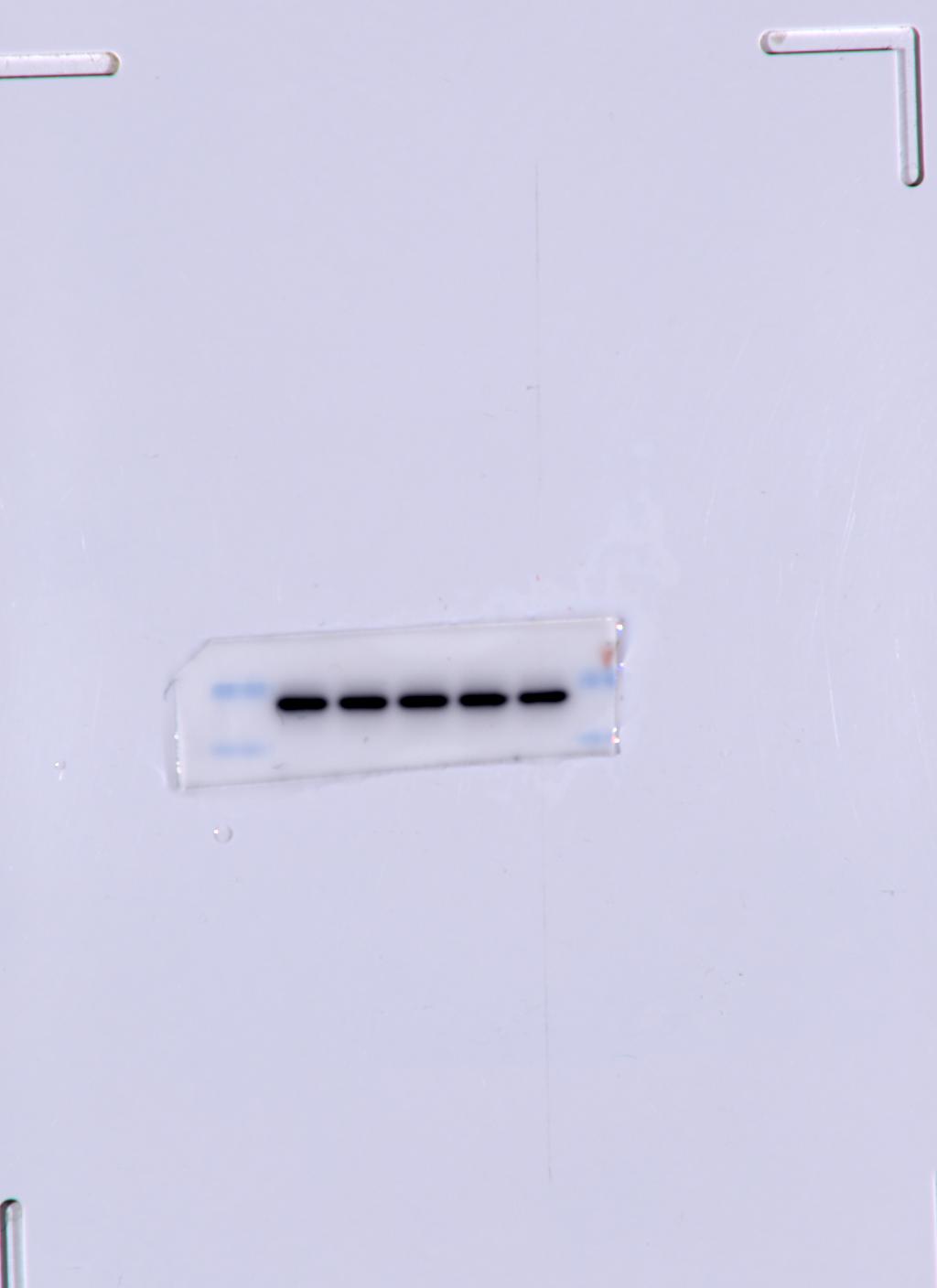

Supplement: Supplementary file 6 [file DataSheet4.ZIP › Fig4一/Fig4C,L,WB.docx]
