# Supplementary material for: Propofol Protects Myocardium From Ischemia/Reperfusion Injury by Inhibiting Ferroptosis Through the AKT/p53 Signaling Pathway
Source: Front Pharmacol. 2022 Mar 16;13:841410. doi: 10.3389/fphar.2022.841410 (PMC8966655; doi:10.3389/fphar.2022.841410)

**Supplemental figure S1**: Full scan of the original blots of cropped images shown in Figure5A.

Lane1 and lane7: marker. Lane2: C. Lane3: I/R. Lane4: I/R+P. Lane5: I/R+P+MK. Lane6:I/R+MK.

FTH1  XCT


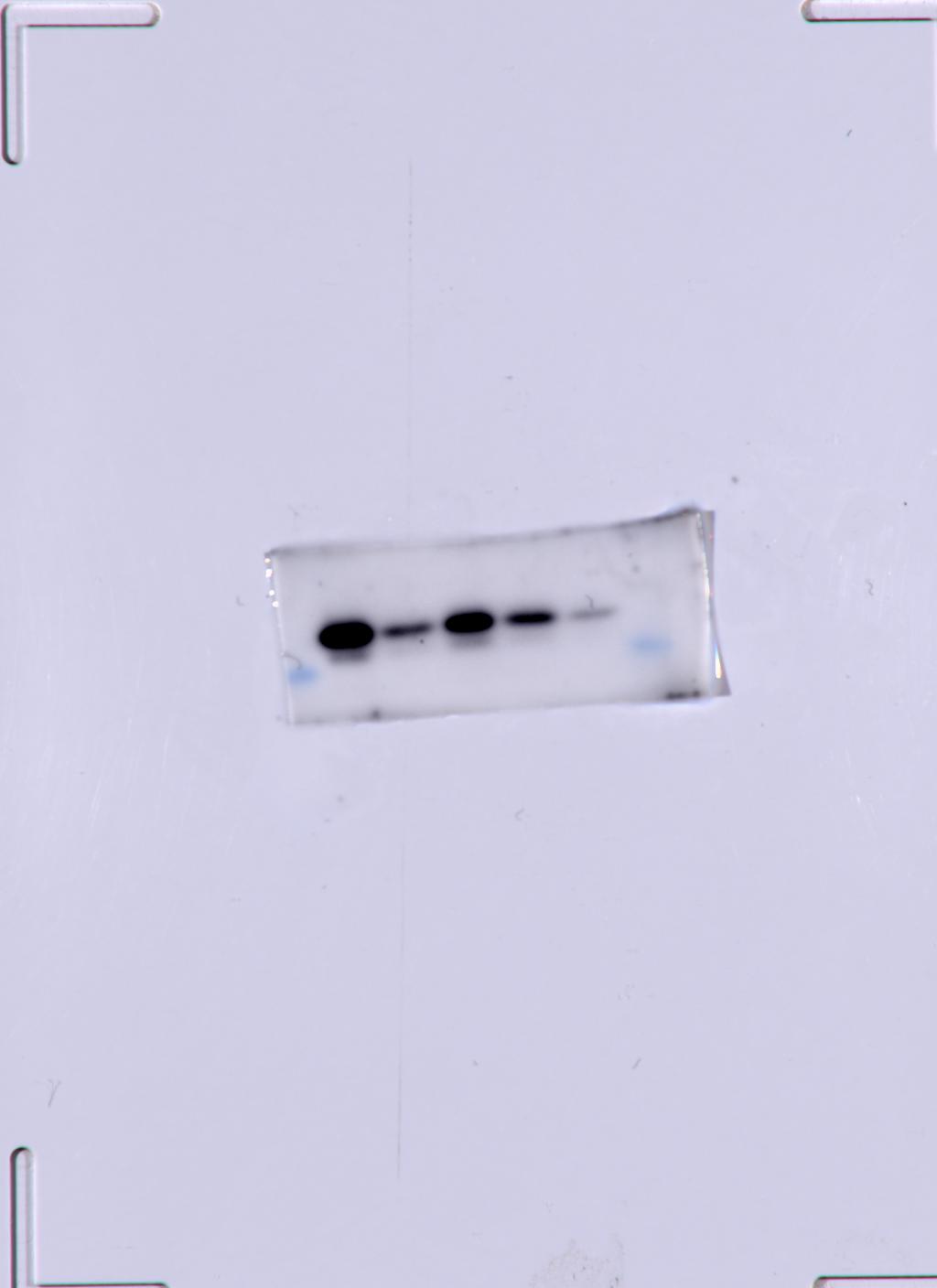

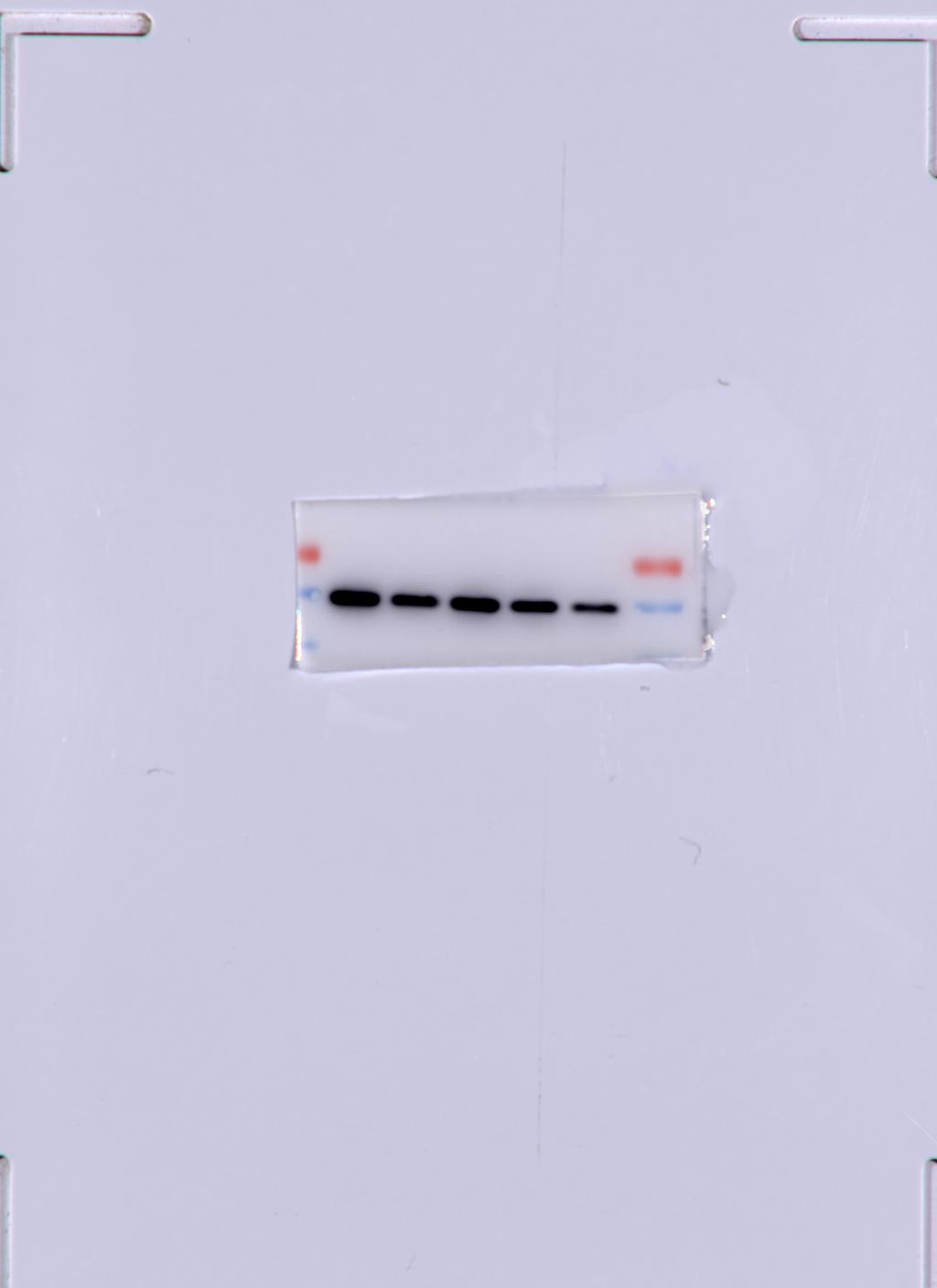


**SOD-2 GPX4**


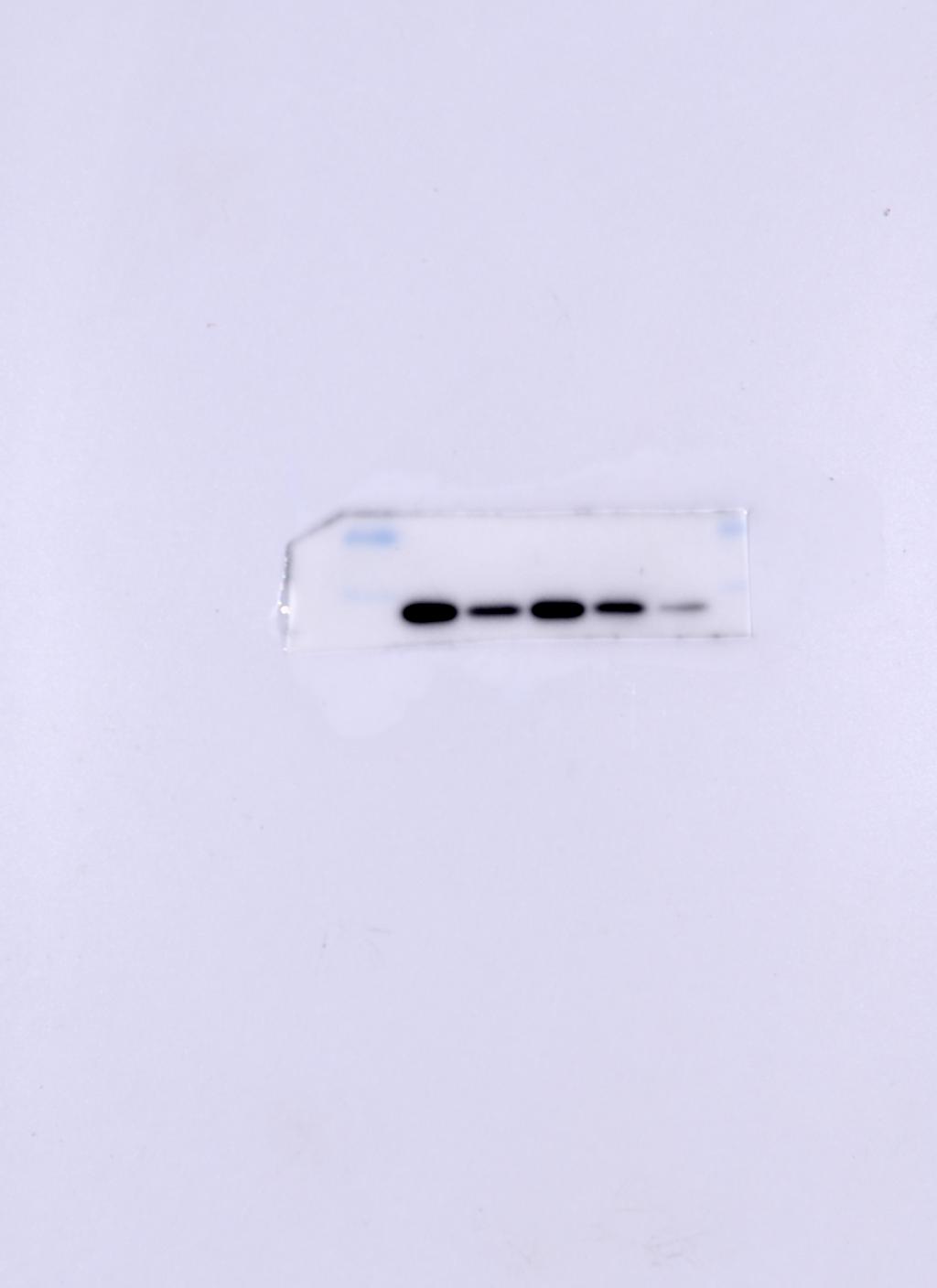

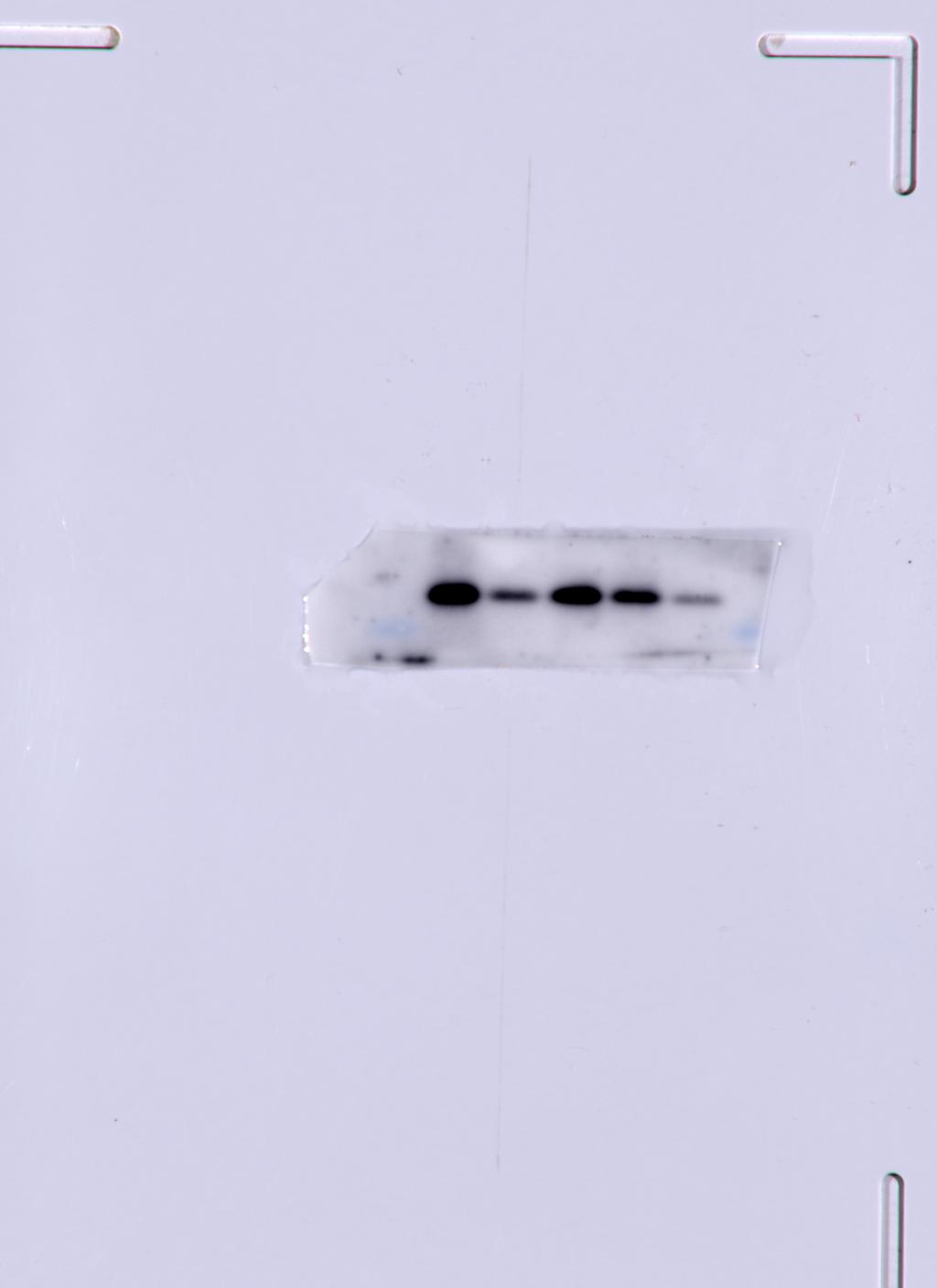


α-tubulin


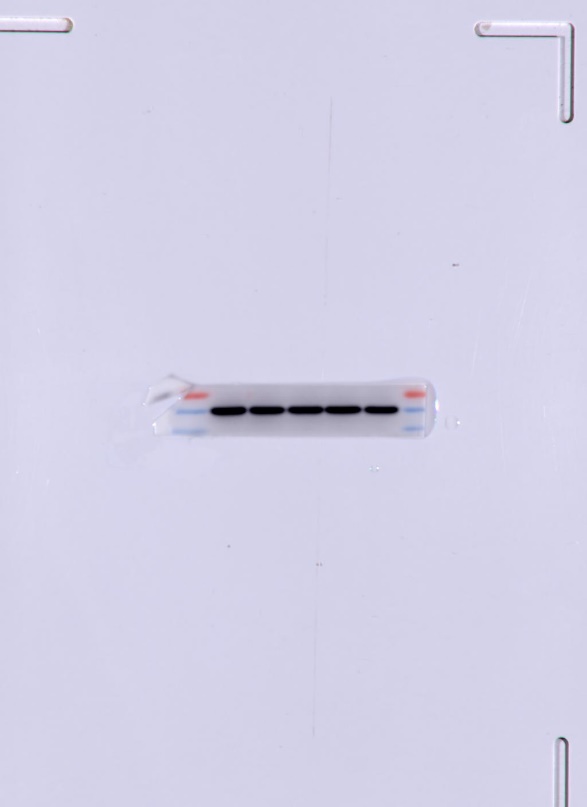


**Supplemental figure S2**: Full scan of the original blots of cropped images shown in Figure5G.

Lane1 and lane7: marker. Lane2: C. Lane3: I/R. Lane4: I/R+P. Lane5: I/R+P+MK. Lane6:I/R+MK.

P-AKT AKT


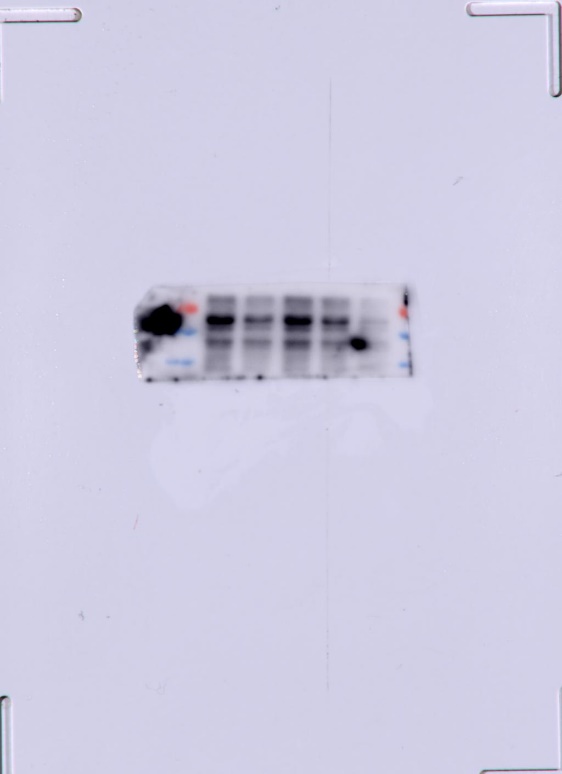

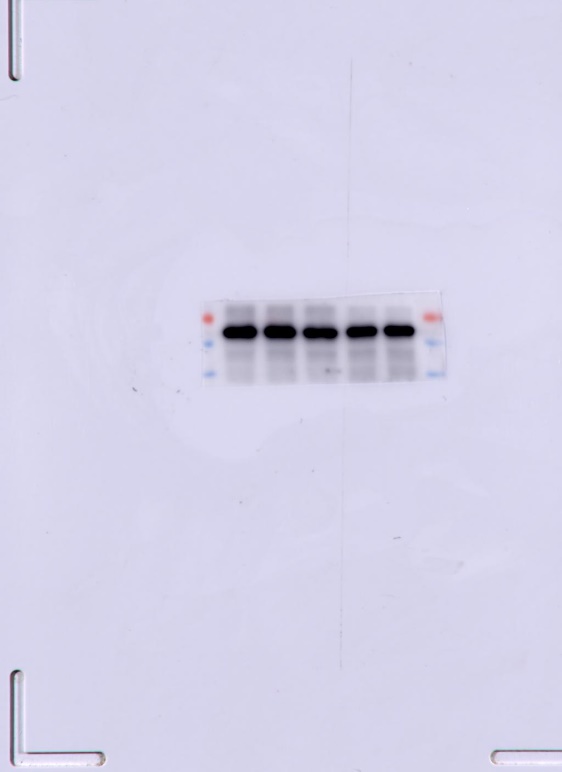


α-tubulin


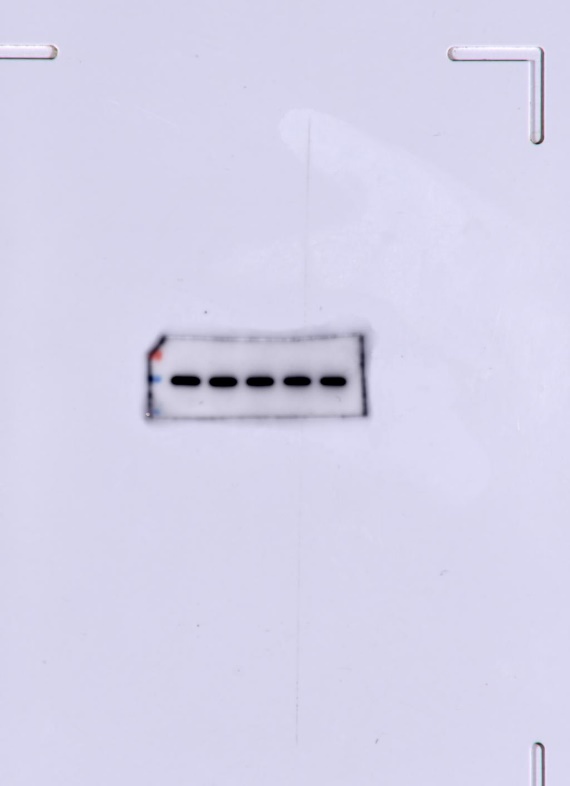

Supplement: Supplementary file 11 [file DataSheet12.ZIP › Fig5一/Fig5A,G,WB.docx]
