# Supplementary material for: Propofol Protects Myocardium From Ischemia/Reperfusion Injury by Inhibiting Ferroptosis Through the AKT/p53 Signaling Pathway
Source: Front Pharmacol. 2022 Mar 16;13:841410. doi: 10.3389/fphar.2022.841410 (PMC8966655; doi:10.3389/fphar.2022.841410)
Supplement: Supplementary file 12 [file DataSheet2.ZIP › Fig2/Fig2B,E/E.pdf]

Std Size

样品ID:	2 2	用户ID:	user
采样ID:	1	测量时间:	2020/11/17 17:49:01
产品编号:		稀释比例:	1:1
总细胞浓度:	3.55*10^6/ml	活细胞浓度:	2.40*10^6/ml
总细胞数:		细胞活率:	67.64%
平均直径:	15.30μm	平均圆度:	0.81
结团率:	28.84%		

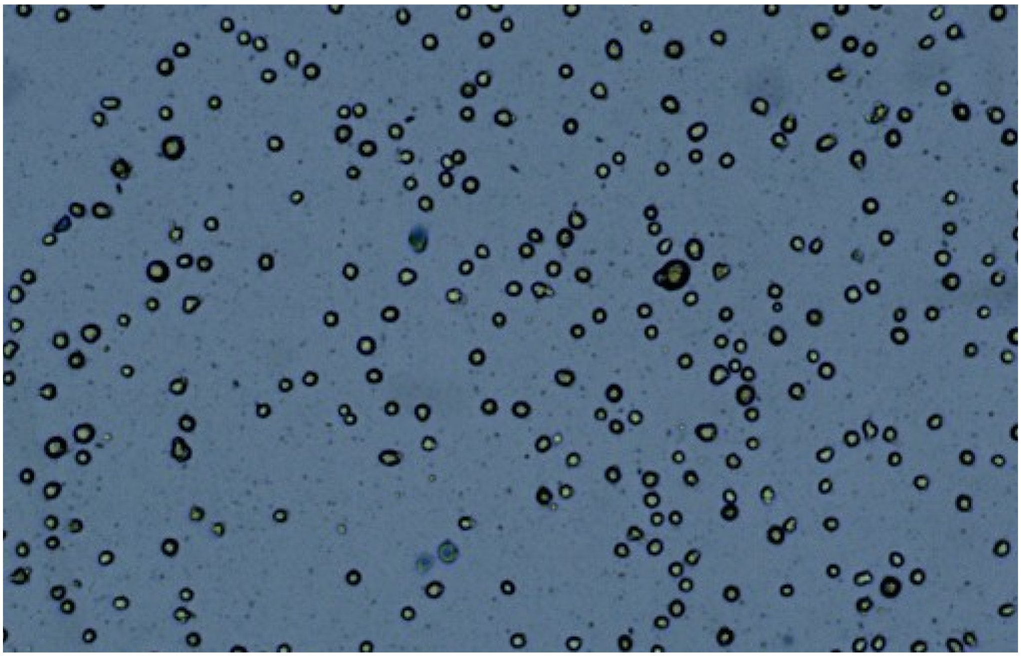

采样图

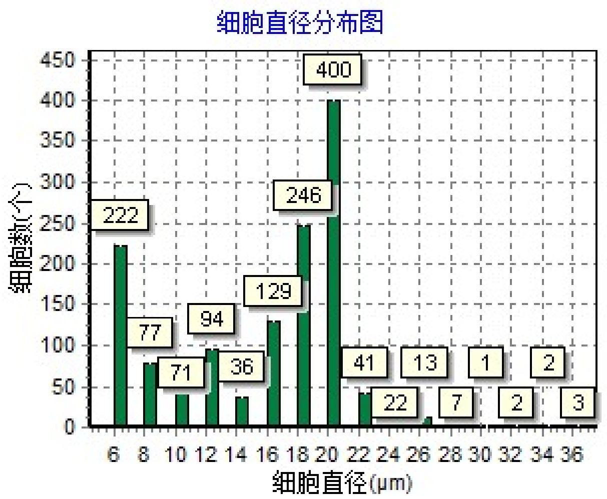

细胞直径分布图

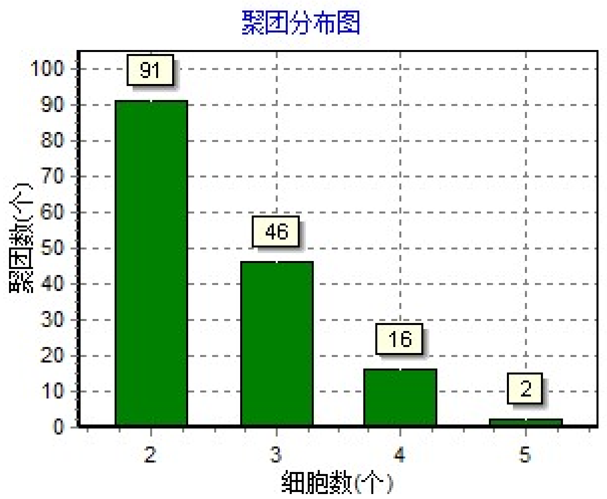

聚团分布图
