# Supplementary material for: Propofol Protects Myocardium From Ischemia/Reperfusion Injury by Inhibiting Ferroptosis Through the AKT/p53 Signaling Pathway
Source: Front Pharmacol. 2022 Mar 16;13:841410. doi: 10.3389/fphar.2022.841410 (PMC8966655; doi:10.3389/fphar.2022.841410)

**Supplemental figure S1**: Full scan of the original blots of cropped images shown in Figure2 I.

Lane1,6: marker. Lane2: C. Lane3: E. Lane4: E+P.Lane5:E+DMSO.

FTH1 SOD-2


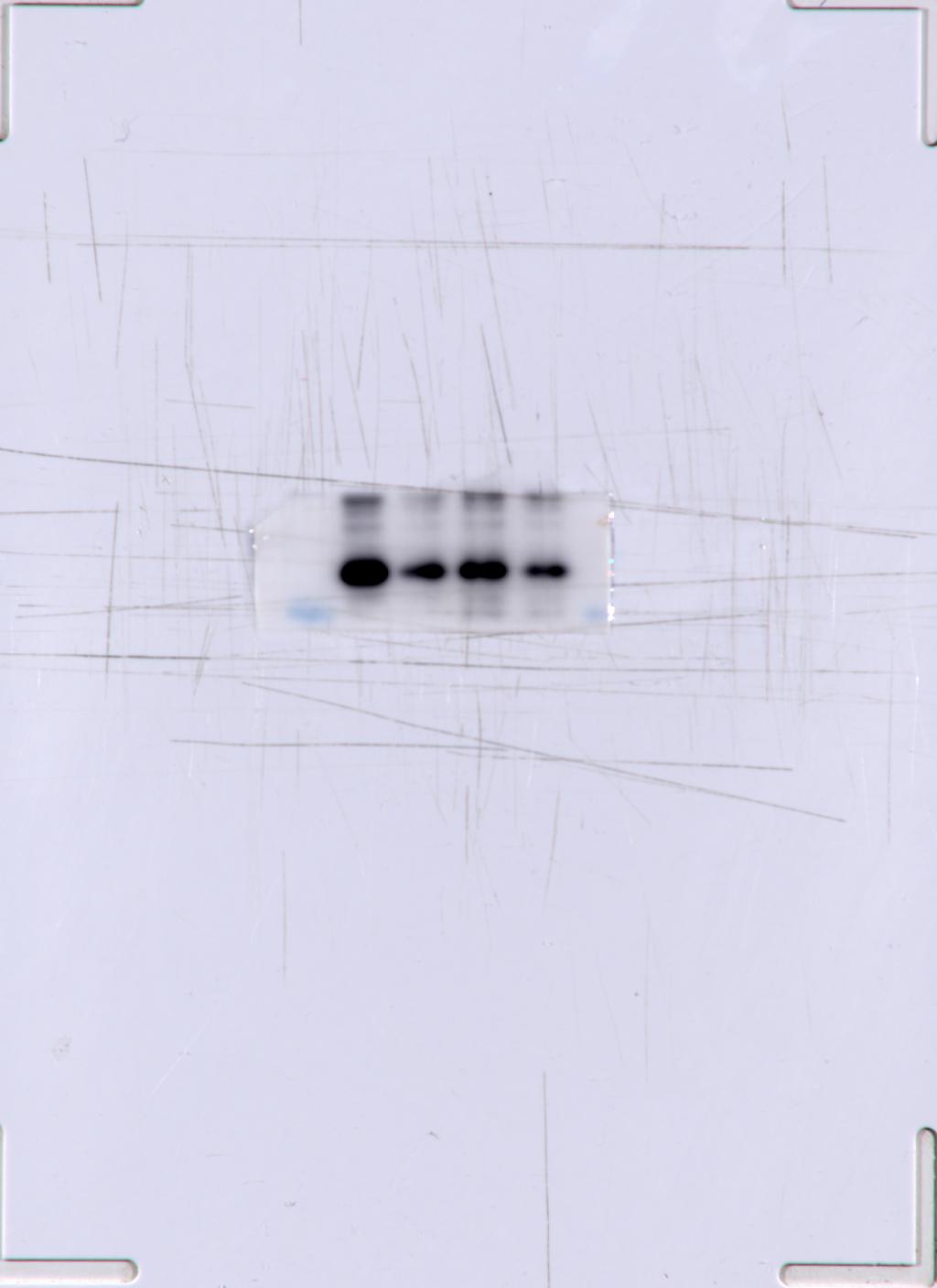

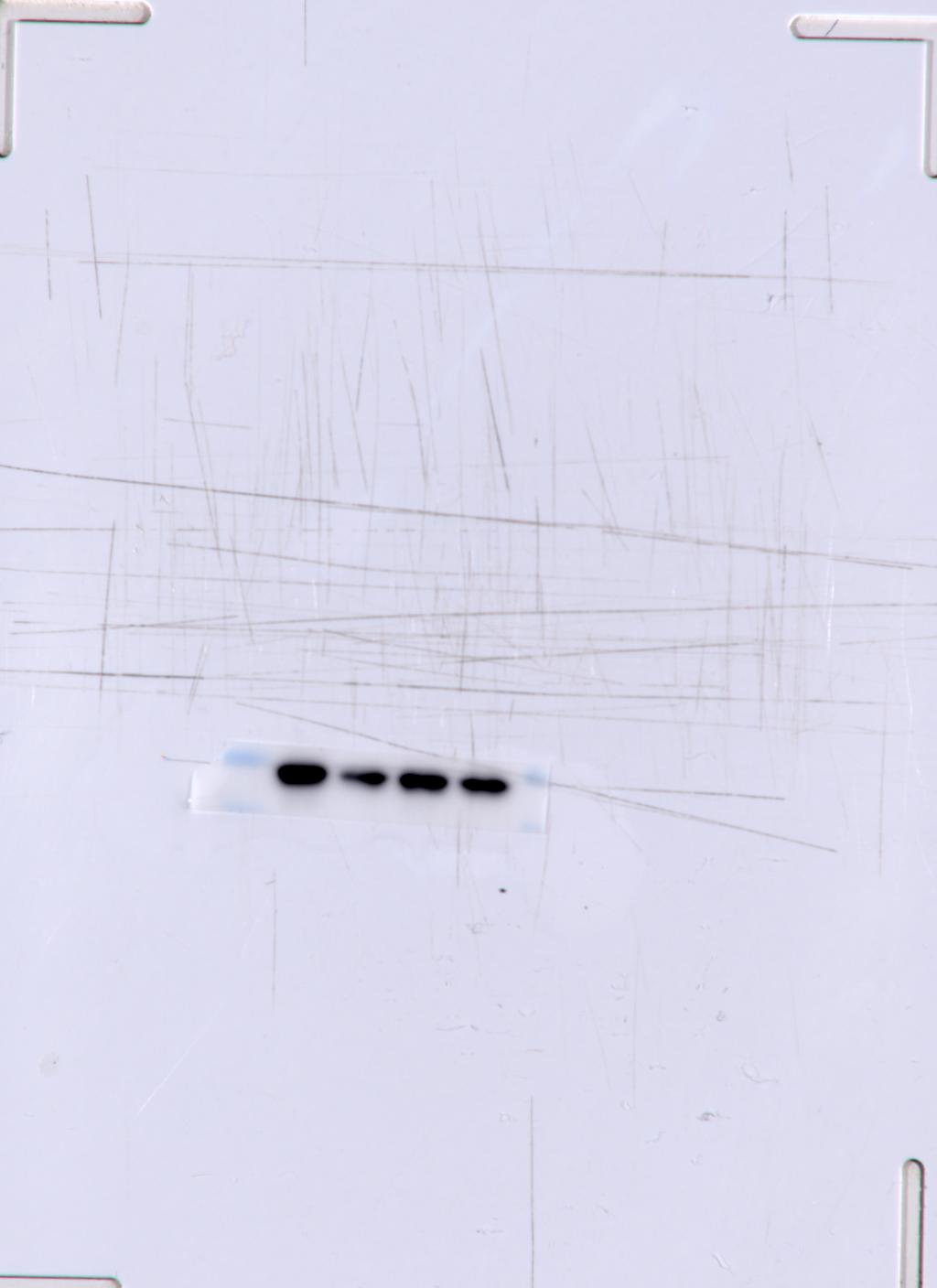


GPX4 α-tubulin


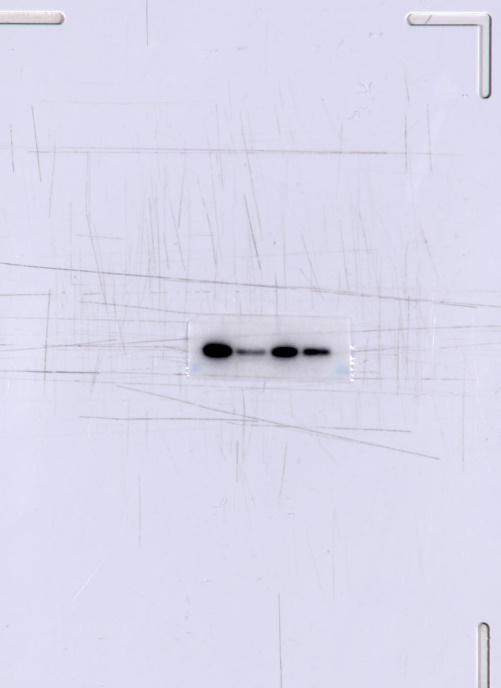

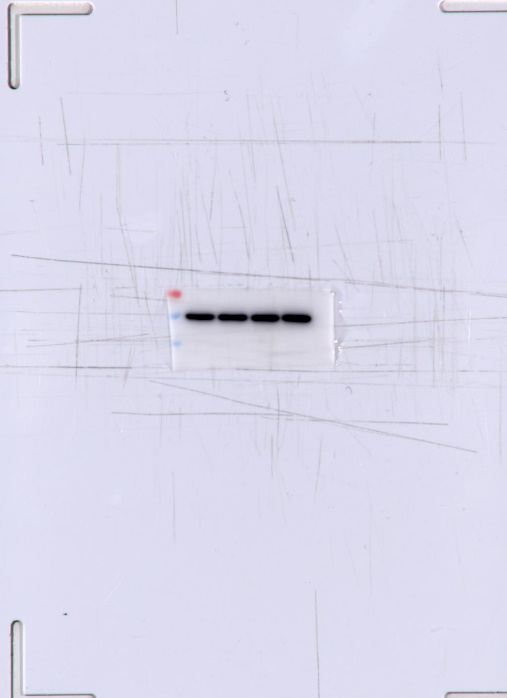

Supplement: Supplementary file 12 [file DataSheet2.ZIP › Fig2/Fig2I-L/Fig2.WB, DataSheet.docx]
